# The importance of genes does not predict their mutation rate

**DOI:** 10.1371/journal.pbio.3004089

**Published:** 2026-11-12

**Authors:** Sofia Radrizzani, Laurence D. Hurst

**Affiliations:** Centre for Evolution, Department of Life Sciences, University of Bath, Bath, United Kingdom; National Centre for Biological Sciences, INDIA

## Abstract

The convention that mutation is a random process has been challenged by suggestions that selection has acted on between-gene variation in the mutation rate, with claims that more important genes are protected from mutation in part, in prokaryotes, by being on the leading strand, while those required for rapid adaptive evolution have higher mutation rates, in part by being on the lagging strand. The original selectionist claims have proven contentious with several replication failures. However, none of the replication attempts fully allow for covariates and many employ data from mismatch repair (MMR) knockout strains. To address these limitations, we consider amassed mutation accumulation data for wild-type *Escherichia coli*, the focal species for adaptive claims, and *Saccharomyces cerevisiae*, in a multiple parameter-controlled analysis to predict between-gene variation in mutational density (mutations per base pair). Along with covariates, we consider putative selectively relevant parameters: protein level and essentiality to capture genic “importance”, and, in *E. coli*, strand with respect to DNA replication. We consider deviation of correlation and partial correlation values from those expected under trinucleotide-controlled simulations. While in *E. coli* more highly transcribed genes may have slightly lower mutation rates, potentially consistent with a selectionist model, the same is not seen in yeast, and in neither species do genes for more abundant or essential proteins have lower mutation rates. In *E. coli* there is no difference in mutation rate between genes on different strands, a lack of difference replicated in other prokaryotes. The degree of purifying selection is also not predictive of mutation density. We additionally highlight a strong artefact seen using the standard off-the-shelf statistical approach and note that results from MMR-deficient strains are discordant. We conclude that as trends are nearly uniformly unsupportive, not consistent between species and of miniscule effect size, they present no challenge to convention.

## Abstract 2

Is mutation a random process, or are more important genes protected from mutation, as has been claimed? This study uses high-quality mutation accumulation data to show that there is no evidence that genic importance predicts mutation rate variation.

## Introduction

While the canonical view of the evolutionary process is often claimed to consider mutation to be a “random” process and selection to be a biasing filter acting on those mutations, there have been numerous suggestions that mutation may not be as random as assumed [1–5], although what random and non-random mean is not always clear (for review see [6]). Despite such ambiguities, claims of non-randomness of mutation are commonly presented in a manner that suggests that classical evolutionary theory is somehow importantly wrong. On presenting evidence that essential genes have lower mutation rates, it was for example concluded that such a result challenges *“the prevailing paradigm that mutation is a directionless force in evolution”* [2]. On claiming to find that “*bacteria can choose which mutations they should produce*”, Cairns and colleagues surmised that “*almost anything seems possible*” [4].

There have been two important related debates concerning the non-random nature of mutation. On the one hand are issues as to whether, especially in a microbiological context, the association between stress and mutation is adaptive [7]. The second, and the focus of the present analysis, is the broader possibility that variation in mutation rate between genes in a genome is the result of selection (e.g., [1–3]). That within a genome genes differ in their mutation rate is not in question [8,9]. Leaving aside effects of being on sex chromosomes [10], numerous explanations have been provided for such variation [8,9]. While many attempts to understand such variation are mechanistic (see, e.g., [11]), since Cox [12] hypothesised that it would be selectively advantageous for more important genes to have a low mutation rate, there have been (numerous) reinventions of the notion that some variation is the product of selection and argued to challenge paradigms of random mutagenesis (e.g., [1–3]).

Broadly, these adaptive models all argue that selection affects gene-level mutation rates in a manner that amplifies the direction of selection on mutations in those genes. On the one hand this means that more “important” genes (defined as highly expressed and/or knockout lethals, i.e., essential genes), that are presumed to be under stronger purifying (negative) selection, are claimed to have lower mutation rates, thereby reducing the number of deleterious mutations, claims made for bacteria [1] and eukaryotes [2,13]. Conversely, genes in which mutations may promote adaptation, those under positive selection, are conjectured to have a high mutation rate [3,14,15]. In bacteria, where the direction of replication is fixed, selection may favour genes for which a higher mutation rate may be beneficial to be on the replicatory lagging strand, under the presumption that error-prone repair following DNA-RNA polymerase collisions causes mutation [3,14] (for review see [16]). This same effect is argued to be part of the mechanism by which essential genes might be relatively protected from mutation [16], as in many bacteria they are more commonly on the leading strand [17,18]. These effects are sometimes claimed to be easy to identify because they are unsubtle. There is, for example, claimed to be a 2.5 times higher rate on the lagging strand than seen for comparable genes on the leading strand [16]. Monroe and colleagues [2] similarly report, in *Arabidopsis*, that essential genes have a mutation rate per base pair (bp) up to half that of other classes of gene.

While adaptive models can be questioned on the basis of whether selection can ever be efficient enough to modify mutation rates on a limited sample of genes [8,19], and whether an increase in rate need be beneficial as many mutations will be deleterious, the more acute problem is whether the claimed results are robust. There are multiple issues.

First, even within the original claims, the data are not obviously consistent with the hypothesis in question. For the strand effect, for example, there is claimed to be a difference in the rate of protein evolution for genes on the two strands, but there was no effect seen when considering the synonymous rate of evolution [3], the more obvious proxy to the mutation rate. Similarly, Monroe and colleagues’ claims of lower mutation rates in essential genes [2] is argued to be an artefact of questionable mutation calling and data handling [20,21], the same effect not seen when their higher-quality data is considered [21], nor replicated in other species (yeast and humans) [20].

Second, many studies, especially older ones, employ proxies such as the synonymous substitution rate [22] (that assumes synonymous mutations are neutral) or circulating single-nucleotide polymorphism (SNP) rates (e.g., [1,13]). Both proxies have the problem that selection may well have acted prior to sampling, thus the profile will be biased. This problem is especially acute for determining whether essential genes have a low mutation rate [23], as mutations affecting essential genes are the first to be eliminated by selection [24], likely because absence of the functional gene product is, by definition of essentiality, cell lethal. This problem may be particularly acute in multicellular organisms in which within-organism cell selection can lead to undetectable mutations, especially when in essential genes [23]. That processes such as biased gene conversion [25] also act on circulating SNPs and affects substitution profiles adds to the difficulty in interpreting results, it being notable that most species have a GC content above that predicted from mutational profiles [26].

Third, some of the original analyses made questionable inferences given their statistical tests. Notably, Monroe and colleagues [2] applied Tajima’s *D* test on population genomic data to claim that this supported the hypothesis that more important sequences have lower mutation rates. Charlesworth and Jensen [27], however, showed that this application employed numerous dubious assumptions and that the results could also be explained by expected differential degrees of purifying selection.

Fourth, there are multiple predictors of the mutation rate that covary, potentially obscuring interpretation of results even if mutation calling is accurate. GC content, especially CpG content, is a determinant of mutation rates on a nucleotide-by-nucleotide basis in many species [8,9,26,28–31]. When a genic property, such as essentiality, covaries with GC content, this same property may then correlate with variation in the mutation rate while not itself causative of the variation. Further, in eukaryotes, genes that replicate late in the cell cycle may have a higher mutation rate [32–39]. A diversity of possible mechanisms has been suggested including a switch in the repair machinery [34,39,40], nucleotide exhaustion [41] (cf. [42]), or altered methylation [33]. Importantly, in bacteria highly expressed genes tend to be closer to the origin of replication (so replicate early) [43], most likely owing to selection on protein dosage, domains near the origin being effectively diploid for longer. This position-expression covariance then has the potential to confound interpretation of gene expression-mutation rate correlations. Whether the mutation rate is lower closer to bacterial origin of replication is also in question as others report a fluctuating pattern between origin and terminus [42,44]. In turn, whether more “important” genes, assayed as highly expressed genes, have a lower mutation rate is confounded by immediate effects of transcription on mutation, as opposed to selection operating on genes for more abundant proteins. Whether transcription is associated with a high versus low mutation rate is, however, unclear with mixed results (see, e.g., [1,19,45–52]). The balance between transcription coupled mutation and repair is here of central importance. For example, genes expressed during spermatogenesis display lower mutation rates on the template strand, attributed to transcription-coupled repair scanning the germline genome [51] with the transcription-coupled repair-associated strand asymmetry extending from the 5′ end through the gene body [52].

Fifth, even if predicted effects are seen, the question remains whether the “adaptive” mutation rate inference is correct. For example, any bias as regards strand may be an artefact of a few highly mutagenically-predisposed genes being on one strand [53], and not a general pattern.

Given all of these issues, it is possibly no surprise that the original adaptive claims have proven contentious with numerous failures to replicate key results, notably effects of expression level/essentiality (see, e.g., [19–21,48,51,53]) and strand [53–55]. There are, however, multiple issues with the reanalyses and the original claims remain defended [16,56,57]. For example, while Monroe and colleagues [2] claimed that essential genes have a lower mutation rate using a massive number of mutations of uncertain validity, reanalysis [21] using only well-resolved mutations had so few mutations that the analysis was greatly underpowered. To overcome a similar problem in bacteria, wherein wild-type (WT) lines have such low mutation rates that mutation accumulation (MA) lines report few mutations, it has been commonplace to analyse data from MA lines in which mismatch repair (MMR) machinery has been deleted, this causing a substantial increase in the mutation rate [19,55,58]. On this basis, for example, it was argued that, in *E. coli*, more highly transcribed genes have a higher, not lower, mutation rate [19] and that there is no difference between strands in their mutation rates [55]. Others also report no strand effect but also no correlation between expression level and mutation rate [53]. In other species, there may be no correlation between mutation and expression when MMR is missing [59]. While analyses of WT lines are typically underpowered, the employment of MMR-deficient lines may be biasing. Their use to deduce the mutation-transcription correlation is, for example, questionable as prior evidence has found that MMR is more active in transcribed genes [60], causing lower mutation rates [61]. Although transcription can induce some mutations, notably C to U mutations, these are mostly repaired by uracil-DNA glycosylase [62]. That the mutation bias in MMR-deficient strains (AT → GC bias) reverses that seen in WT strains (GC → AT) [58] questions further whether WT trends should be extracted from such data. As expression levels also differ between genes on leading and lagging strands (see below), usage of MMR-deficient strains could similarly interfere with attempts to determine whether the two strands have different mutation rates.

This difference in expression level of genes on the different strands points to a further general issue, this being that, even if handling well-called WT mutations, showing whether a given parameter is relevant is non-trivial as it requires exclusion of the many covariates affecting a gene’s mutation rate. For example, essential genes tend to be highly expressed [63], on the leading strand [18] and near the origin of replication [63], all of which may contribute to mutation rate variation. We are unaware of any analysis attempting to control for all putative variables.

Here then we return to the issue of whether gene-level mutation rate variation is predicted by genic importance and, in prokaryotes, strand. In the first instance, we focus on *E. coli*. To overcome the problem of limited numbers of mutations, we assemble a concatenated set of mutation calls from WT MA lines [58,64,65], the focal species for adaptive mutation rate claims [1,3,19,57] and one with a large effective population size, and hence most likely to have adaptive mutation rate variation [8]. As done previously [64], we also include mutations from lines that, despite having genes knocked out, have a rate and spectrum equivalent to that of wild types in the stricter sense (we also check results for resilience to exclusion of mutations from such lines, see below). These non-WT non-MMR knockout (KO) types we refer to as non-MMR KO lines. This results in a data set of over 3,500 well-resolved spontaneous mutations, over 2,500 in the coding sequence (CDS), in WT strains that all have approximately the same mutation rate (Fig 1A). The first data sets [58,64] we refer to as Foster data, the later [65] as Wei. While there are further experiments that provide a few extra WT mutations [66–68], the numbers are too limited to perform robust tests of selection on. We therefore include these to check for robustness of results alone, and not as the focal data set.

**Fig 1:**
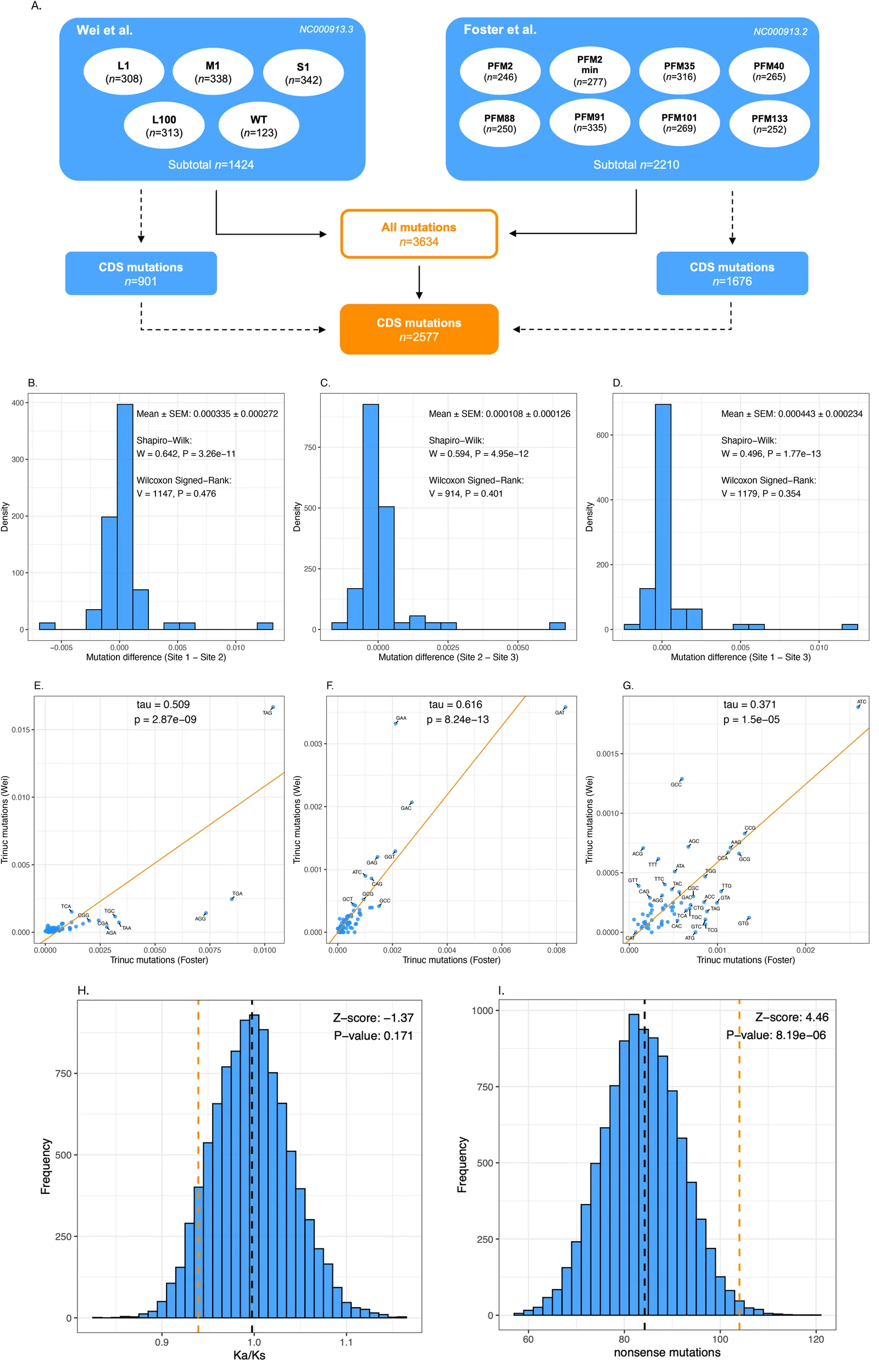
Mutation accumulation data merging and mutation profiles. **(A)** Breakdown of mutation accumulation data combined from WT strains generated by Foster and colleagues [64] and Wei and colleagues [65]. Mutations are assembled as those occurring in the whole-genome sequence and then filtered to include only those in the coding sequence (CDS). The proportion of CDS mutations contributed by each data set to the full CDS set are Foster:Wei 0.65:0.35. For Foster data, mutations are tracked back to *E. coli* genome NC_000913.2 (with 4321 genes and 4073876 total CDS bp), for Wei data NC_000913.3 (with 4308 genes and 4005730 total CDS bp). See S1 Table for mutation annotations per gene. **(B–D)** Distribution of trinucleotide mutation differences for each pairwise codon site comparison. Shapiro-Wilk normality test results displayed on the plot, as well as those of a paired Wilcoxon Signed-Rank test. Trinucleotides were formed such that the middle focal base is the mutating one and can occur at within-codon site 1, 2, or 3 (as labelled). Despite non-normality, we include mean and standard error of the mean (SEM) for information. **(E–G)** Scatter plots of trinucleotide mutations recorded by Foster and colleagues [64] and Wei and colleagues [65]. Line represents orthogonal regression between the two data sets, and a Kendall’s tau and respective *P*-value are displayed in each plot. Trinucleotides were formed such that the middle focal base is the mutating one and can occur at within-codon site 1 (panel D), 2 (panel E), or 3 (panel F). For panels B-G, the trinucleotide mutations are determined per occurrence of the trinucleotide in the respective protein-coding genome: for Foster data genome accession NC_000913.2 and for Wei data NC_000913.3. **(H)** Ka/Ks estimation for observed and simulated mutation data. **I.** Number of nonsense mutations for observed and simulated data. For H–I, mutation data merged from Foster and Wei data, see S3 Fig for separate results for each genome version. The values found for each of the 10,000 simulated mutation densities are shown in the blue histograms and their mean by the black line, whilst the value from the observed mutation density by the orange line. The *Z* score displayed at the top right of each plot represents the deviation of observed from the simulated null calculated by subtracting the mean simulated value from the observed value and dividing it by the standard deviation of the simulated values. The *P-*value displayed is inferred from the *Z* score via R’s pnorm function under a two-tailed assumption. The data and code needed to reproduce this Figure can be found in https://doi.org/10.5281/zenodo.23263169.

For such MA lines, comparison of synonymous and non-synonymous mutation rates commonly report no deviation from null [64] and absolute rates are higher than for less direct analyses [58], suggesting a closer proxy to the true underlying rate. To more closely examine whether MA data provides a good proxy to the true mutational profile, we start by testing for evidence that purifying selection may have acted on these mutations by considering three tests: whether given trinucleotide context codons sites 1, 2 and 3 have the same mutation rate; whether the ratio of non-synonymous to synonymous substitutions (Ka/Ks) is different from trinucleotide-controlled simulations; whether the observed frequency of premature stop (nonsense) mutations is lower than expected under trinucleotide-controlled simulations.

To address the central problem of whether genic importance predicts mutation rate, we start by considering the number of mutations in each gene normalised by gene size, giving a per-base measure (as done by prior analyses: S1 Text). This metric we call mutational density to avoid any possible confusion with usage of the term mutation rate as the latter may, but need not, additionally imply a temporal dimension, i.e., mutational density per unit time/generation (note the above usages of the term mutation rate are equivalent to our mutational density as most also have no temporal dimension). Neither the predictor variable data nor the mutational density data accord with assumptions of parametric approaches with or without transformation, commonly owing to an abundance of zeros. Thus, to obviate the covariation problem, and to provide an analytical approach that accords with prior analyses (S1 Text), we consider non-parametric partial correlations between a series of putative predictors (cf. [19,20]). However, as Spearman’s rho, the more commonly applied statistic, misestimates when there are multiple ties, and Kendall’s tau is more robust, we employ the latter. We find that application of such off-the-shelf pairwise and partial correlation analysis nonetheless results in strong artefacts. To overcome this, we employ a simulation approach to derive null expectations and consider deviation from the null in Z units as the reporting statistic. This Monte Carlo approach uniquely controls for the trinucleotide context of all mutations, a determinant we find to be especially profound with an order of magnitude of difference in mutability of the least and most mutable trinucleotides. This being said, as a resilience check, we also consider, without trinucleotide control either Poisson regression on mutation counts per gene in yeast (where there is no over-dispersion) or negative binomial regression in *E. coli* where count data is over-dispersed (but with no zero excess).

As covariates, we consider GC content, CpG deviation from that expected by mononucleotide frequencies, transcript abundance, relative distance from the origin of replication (in two modes) and gene size. We consider the latter since the most 5′ end of genes (first 10 codons in CDS) have a mutation rate around a half that of the gene body, only in part because in *E. coli* 5′ ends are AT-rich and A and T are less mutagenic [28]. Whether more “important” genes, assayed as highly “expressed” genes, have a lower mutation rate is confounded by immediate effects of transcription on mutation, as opposed to selection operating on genes for more abundant proteins. This motivates the choice of RNA level as a covariate, with protein level being the “adaptive” parameter and measure of genic importance. This contrasts with the analysis of Martincorena and colleagues [1] who employ RNA level as a measure of a gene’s importance, finding a lower mutation rate (using circulating SNPs) in more highly expressed genes. Our assumption is that it is protein titre that would mediate any activity of selection on the mutation rate as it is properties of the proteins that are under selection the most. In support of this, a gene’s expression level (assayed either by protein level or indirectly via RNA level) is the strongest predictor of a protein’s rate of evolution [69], largely mediated by effects that act on the proteins, not the RNAs [70]. Similarly, in mammals, the rate of evolution of long non-coding RNA is correlated with expression level, but the strength of the correlation is extremely weak, an order of magnitude lower than that of protein-coding genes [71], again reinforcing the relative importance of protein-level properties to the action of expression-mediated selection. As protein titre and RNA level covary, if selection is crafting the mutation rate variation, we expect both parameters to predict mutational density.

Our results fail to support selectionist claims but also disagree with prior results derived using MMR-deficient lines. Might this be owing to the use of MMR-deficient lines per se or other differences? While loss of transcription-coupled repair appears not to affect mutation rates in MA lines [64], the effect of MMR knockout on predictors of the mutation rate—and how these differ from WT—is not well resolved. Here then we additionally ask whether analysis of MMR knockout strains and WT strains (the ones more relevant to claims of adaptive mutation rate variation) qualitatively differ when all other variables are the same. We find they do in ways important for the debate. Notably, the direction of the transcription-mutation density effect can change, a direction allowed to reject an adaptive hypothesis [19] (see also [47]). This cautions against using MMR knockout strains as a proxy to mutation patterns in WT strains.

To consider the generality of these results, we ask in other prokaryotes whether we see any evidence of any effect of strand—we don’t. In addition, we consider *S. cerevisiae* for which there also exists broad knockout and proteomic coverage. We see the same statistical artefacts as reported for *E. coli*, so again consider trinucleotide-based simulations. We again see no evidence that genes for more abundant proteins nor essential genes have lower mutation rates, while we confirm that genes that replicate late in the cell cycle have higher mutation rates [34,39]. In neither focal species do we see the extent of purifying selection on a gene to predict its mutation density. There may be weak correlation trends with respect to transcription that are of opposite directions in yeast and *E. coli.* Overall, we see no clear evidence that genic importance is a readily detectable predictor of a gene’s mutation rate: if there are such effects, they are so weak as to explain almost none of the variation in mutation rates between genes.

## Results

### Mutation accumulation data is (probably) free of the influence of selection

We start by focussing on *E. coli*. Given that a problem with many proxies of the mutation rate is that they are likely to be importantly affected by purifying selection, hence not appropriate to answer questions relating genic importance to the mutation rate [23], we commence by testing for the influence of purifying selection. While MA lines are constructed to be largely unaffected by selection, this need not be the case [72,73]. We consider three tests for the activity of purifying selection on the reported mutations.

In bacteria, MMR is focussed on gene bodies, hence intergenic sequences have a higher mutation rate than CDSs [28,58,64], an effect that is eliminated when MMR genes are deleted [64]. Thus, to consider whether selection operates on the mutations in WT MA lines, it is best to compare mutations likely to be under differential selection in CDS. We first test for deviation from null rates in a manner that respects the sequence context of mutations and permits analysis of all CDS mutations (i.e., nonsense mutations, as well as synonymous and missense ones). As the mutation rate of a nucleotide is conditioned by its flanking nucleotides in *E. coli* (see, e.g., [28] and review [9]), we consider for each of the 64 possible trinucleotides the mutation rate per occurrence of that trinucleotide within CDS, with the central base being at within-codon sites 1, 2, or 3. This has the caveat that they may be even longer-range effects [74]. If purifying selection is operating, we expect observed “mutation” rates to run in the order 3 > 1 > 2, as site 1 and 2 mutations are all (site 2) or nearly all (site 1) non-synonymous while many mutations at site 3 are synonymous. That site 2 mutations more profoundly affect chemical properties of the amino acids [75] reinforces this expected order. If, by contrast, we are recovering mutations in an unbiased manner, we expect all sites to have comparable mutation rates (1 ≈ 2 ≈ 3).

There is however a complexity, namely that, if the three sites differ in their nucleotide content and nucleotides are differently mutable (both of which are true), then average rates of mutation at the three sites may differ but not because of the codon location per se but because of the difference in nucleotide content. To overcome this problem, we consider the trinucleotide context of all mutations and consider, for each codon site (1, 2 or 3), the mutation rate of the central base of the trinucleotide centred on the given codon position, per occurrence of that trinucleotide at that position across all CDS. We can then perform paired tests considering the differences between the 64 trinucleotides at different sites. Rather than asking about a single absolute rate at each of the three sites, we thus instead consider the distribution of the differences between the two paired 64-element vectors.

We thus determined for each codon site, and for the Foster and Wei data separately (see Fig 1A for data set breakdown and S1 Table for mutation annotations per gene), 64-element vectors for mutation rates of the central base of the trinucleotides. We then consider, for each experiment, the difference in mutation rate of the same trinucleotide between the codon sites (1 v 2, 1 v 3 and 2 v 3), each analysis generating a 64-element vector of differences. As the distribution of these fails normality testing (Fig 1B–1D), we evaluate significance by a paired Wilcoxon Signed-Rank test. In no experiment (Foster or Wei) do we detect deviation from null, even without multiple test correction (S1 Fig). When Foster and Wei data are merged, we again see no evidence for deviation from a null of all three codon positions having the same mutation rate (Fig 1B–1D: paired Wilcoxon Signed-Rank test site 1 v site 2 *P*-value = 0.476, 2 v 3 *P*-value = 0.401, 1 v 3 *P*-value = 0.354). We conclude that we see no evidence of deviation from a purely mutational null in either experiment. We note too that for all three codon sites the per trinucleotide rates strongly correlate between the Wei and Foster data (Fig 1E–1G), indicating strong replicability of mutational propensities. This holds true also when comparing trinucleotide mutation profiles without distinguishing by codon site (S2 Fig: Kendall’s tau = 0.516, *P*-value = 1.69 × 10^−9^).

The above, while permitting analysis of all CDS mutations, is however underpowered in permitting only 64 data points. An alternative is to estimate the ratio of non-synonymous to synonymous substitution rates (Ka/Ks) for the observed mutations and compare that to a simulated set (cf [2,58]). To determine the observed Ka/Ks, we produce a multigene CDS concatenation and compare the reference CDS to that incorporating all non-synonymous and synonymous mutations. By necessity, we exclude nonsense mutations. For the simulants, we determine the trinucleotide context of the real mutations and for each qualifying mutation (i.e., not nonsense), randomly select one instance of the same trinucleotide from the CDSs and replace that with the mutated version. Should this result in a nonsense mutation, we reject the mutation and repeat the process until a randomly selected qualifying mutation is found. The simulations thus have approximately the same number of mutations as observed in the real data and the same trinucleotide context profile (exceptions occur when mutations hit the same site more than once). We again concatenate all CDSs and compare mutated to reference sequences to estimate Ka/Ks. We repeat the simulation 10,000 times to derive the null distribution. We compare this null distribution to the observed ratio to determine the significance of deviation from null. Our expectation under the null of no selection is that observed and simulated will be the same and, if the Ka/Ks estimate is perfectly accurate, to be equal to one. If selection is operating, we expect the observed to be significantly lower than the null, reflecting greater elimination of non-synonymous mutations.

We find that the observed Ka/Ks ratio is not significantly lower than the null (Figs 1H and S3A, which is close to one). We thus again find no evidence that the observed mutations are any different from those expected under null, under an assumption of an absence of selection. However, when separated into Foster and Wei data sets, we observe that the Foster data resemble null very closely (S3B Fig) but the Wei data are marginally significantly lower than null (S3C Fig, *P*-value = 0.032, not significant on multi-test correction).

Our third test considers the number of nonsense mutations observed compared with simulations based on trinucleotide mutation context. The null expectation is no difference, while purifying selection predicts fewer nonsense mutations in the observed mutation data than expected by chance as these are the most severe of mutations, under the assumption that these will be removed prior to sampling. In neither the merged experiments (S3D Fig) nor each individual data set (S3E and S3F Fig) do we observe fewer nonsense mutations than expected. Unexpectedly, we find more nonsense mutations than expected by chance, with a significant difference in the merged data (S3D Fig) and the Wei data alone (S3F Fig), potentially indicative of positive selection on nonsense mutations (note we are not so concerned about positive selection as, unlike purifying/negative selection, it need not cause mutations to go unreported). The Wei data set thus appears to underreport non-synonymous mutations (S3C Fig) and over-report nonsense mutations (S3F Fig). Nonetheless, all three tests suggest that we can make the assumption for the merged data that purifying selection is not importantly operating. However, given the unexpected properties of the Wei data, we also check that all results are resilient to analysis of the Foster data alone. We do not know why there is an apparent difference between the Wei and Foster data sets. One possibility is that, if an MA line is run for longer, the opportunity for selection might be higher. However, the Foster data comes from experiments that permitted between 3 and 6 thousand generations [64], while the Wei data comes from MA lines of about 1,500 generations [65].

### Partial correlation analysis finds no evidence for adaptive mutation rates

What then determines the between-gene variation in mutational density? To consider this in the first instance, we employ Kendall’s tau and partial tau, these being unusually robust to tied data (abundant in the data set) and unaffected by non-normality in the data, which is profuse [76].

We consider three “adaptive” parameters (protein level, essentiality, strand with respect to replication) as predictors of mutational density and numerous covariates. Many of the parameters correlate (Table 1), underscoring the need for covariate control. For example, non-essential genes tend to be lowly expressed (at protein and RNA level), short, have low GC (and have fewer CpGs than expected), and lie closer to the terminus and on the lagging strand (Table 1). Likewise, leading strand genes are more highly expressed, more likely to be essential, longer, GC- and CpG-richer (Table 1).

**Table 1:** Full (top) and partial (bottom) Kendall correlation analysis between mutation density and all annotated gene features. Mutation density (“mut_density”) determined as mutation count per gene normalised by gene length. Features include gene length (“gene_len”), GC content (“GC”), deviation of CpG content from that expected by mononucleotide content (“CpG_dev”), relative position from origin (“rel_pos”), gene essentiality classification from the PEC database [77] (“nonessentiality”, scored as 2 for non-essential and 1 for essential), strand orientation relative to the origin (“strand”, scored as 0 for lagging strand, 1 for leading strand), protein abundance from PaxDB [78] (“prot_abundance”), and median transcript abundance from a compendium of RNA-seq data [79] (“RNA_abundance”). Mutation data combined from Foster and colleagues [64] and Wei and colleagues [65] for a total of 2,577 CDS mutations annotated respectively to the reference *E. coli* genomes NC_000913.2 and NC_000913.3. For GC content, CpG deviation, gene length and relative position from the origin, weighted means were used taking into account the proportion of CDS mutations contributed by each data set to the merged CDS mutation data (Foster: 0.65, Wei: 0.35). Stars represent *P*-value (*P* < 0.0001 **** *P* < 0.001 *** *P* < 0.01 ** *P* < 0.05 * *P* > 0.05 no stars). The data and code needed to reproduce this Table can be found in https://doi.org/10.5281/zenodo.23263169.

	mut_density	gene_len	GC	CpG_dev	rel_pos	nonessentiality	strand	prot_abundance	RNA_abundance
**mut_density**		0.127****	0.027*	0.023*	−0.005	0.007	0.013	−0.004	−0.064****
**gene_len**	0.110****		0.228****	0.087****	−0.013	−0.027*	0.029*	0.040***	−0.070****
**GC**	−0.005	0.204****		0.123****	−0.067****	−0.058****	0.054****	0.065****	0.055****
**CpG_dev**	0.018	0.056****	0.093****		−0.016	−0.095****	0.030*	0.159****	0.104****
**rel_pos**	−0.004	0.003	−0.059****	−0.001		0.063****	−0.013	−0.029**	−0.039***
**nonessentiality**	0	−0.028**	−0.022*	−0.052****	0.052****		−0.077****	−0.211****	−0.264****
**strand**	0.004	0.005	0.042****	0.018	−0.005	−0.063****		0.043***	0.039**
**prot_abundance**	0.021*	0.071****	0.012	0.107****	−0.004	−0.078****	0.02		0.507****
**RNA_abundance**	−0.058****	−0.124****	0.033**	0.026*	−0.016	−0.186****	0.005	0.478****	

We can then ask, on controlling for the other variables, which variables predict mutational density. None of the three “adaptive” parameters are predictive before or after covariate control (Table 1 and Fig 2). Gene size is, by some distance, the strongest correlate to mutational density, with longer genes having higher mutational densities (Table 1: full Kendall’s tau = 0.127, *P*-value = 1.71 × 10^−28^; partial tau = 0.110, *P*-value = 5.60 × 10^−25^). On partial correlation, protein and RNA expression level are the only other predictive variables, though, unexpectedly, opposite in direction (Table 1). We draw the same qualitative conclusions when considering Foster data alone (S2 Table).

**Fig 2:**
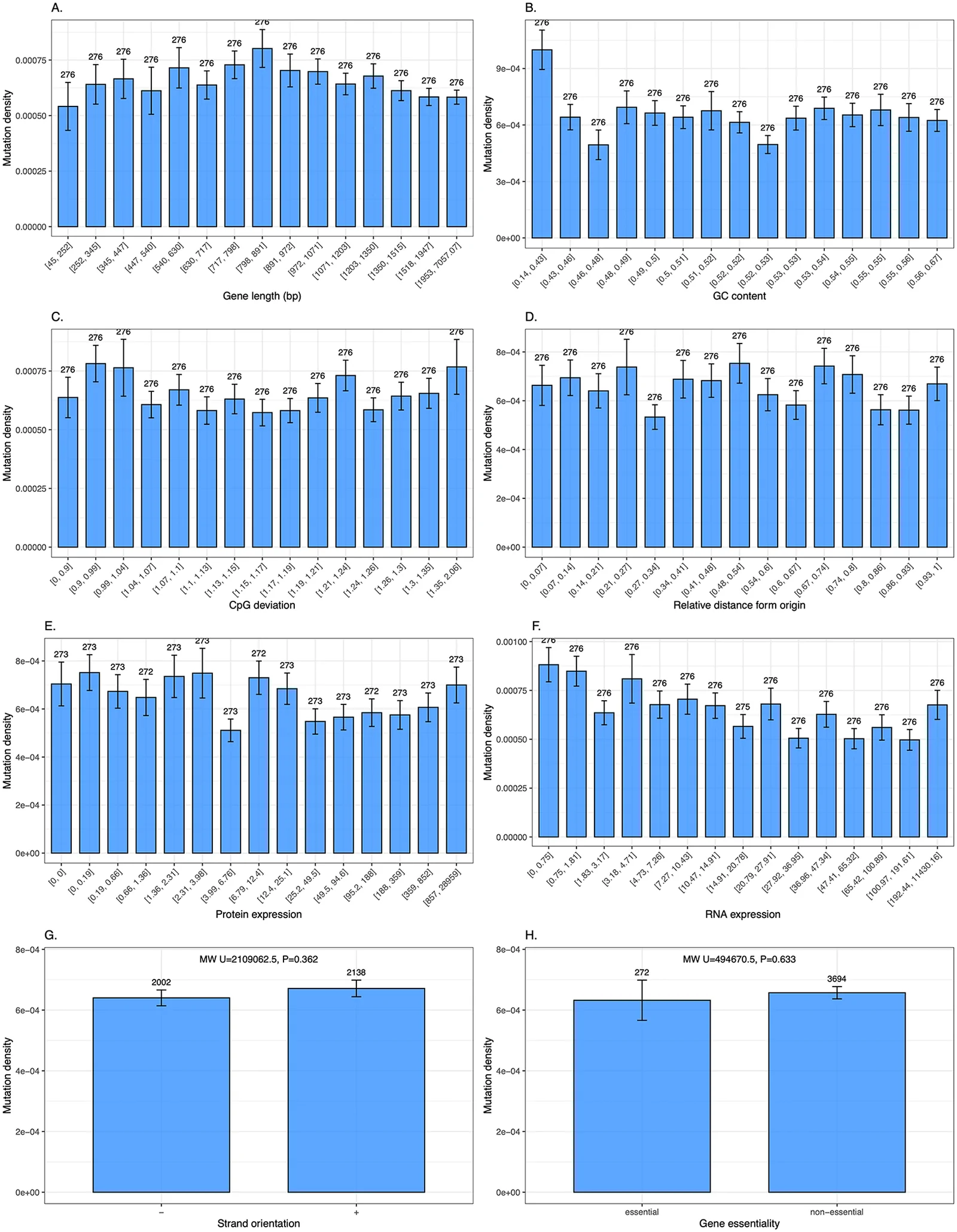
Average mutation density per sequence feature. Mutation density determined as mutation count per gene normalised by gene length. Features include gene length **(A)**, GC content **(B)**, deviation of CpG content from that expected by mononucleotide content **(C)**, relative position from origin **(D)**, protein abundance from PaxDB [78] **(E)**, and median transcript abundance from a compendium of RNA-seq data [79] **(F)**, strand orientation relative to the origin of replication (**G**, scored as 0 for lagging (−) strand, 1 for leading (+) strand), gene essentiality classification from the PEC database [77] (**H**, scored as 2 for non-essential and 1 for essential). For all panels, genes binned according to ranging sequence feature in order to produce similarly sized bins (number of genes in each bin is reported on top of bars). Error bars represent standard error of the mean (SEM). For panels G and H, a Mann–Whitney *U* test was performed and the *U* statistic with respective *P*-value displayed in the plot. For panels A–D, data merged using a weighted mean between Foster and colleagues [64] (with genome accession NC_000913.2) and Wei and colleagues [65] (NC_000913.3), considering the proportion of CDS mutations contributed by each (Foster:Wei 0.65:0.35). The data and code needed to reproduce this Figure can be found in https://doi.org/10.5281/zenodo.23263169.

The above analyses used amassed transcriptomic data and proteomic data. However, gene expression varies with growth medium in a manner that can impact mutation rates, especially if low-nutrient, stressful conditions are considered [80], and indeed within the compendium of RNAseq data we employ, 152 of 3,376 studies mention the word “stress” in their titles (see S3 Table). To allow for this, we restrict the proteomic data to that derived under LB conditions (the conditions of the experiments) and apply the transcript expression data from LB conditions that were employed to support the original claim that higher expression is associated with lower mutation rates [1]. The RNA levels in the amassed data [79] and this LB-only data set [1] are well correlated (S4 Fig: rho = 0.71, *P*-value = 2 × 10^−16^). We find transcript abundance to again be negatively correlated with mutational density prior to partial correlation. It is weakly significant on partial correlation if gene length is permitted as a parameter (S4A Table) and when it isn’t (S4B Table). The protein level is never a significant predictor.

As it has been suggested that mutation rates could show a fluctuating rather than a monotonic pattern as a function of distance from the origin of replication (see, e.g., [42]), we also consider relative distance from the closest midway point between the origin and the terminus. We find that this makes no meaningful difference, as, upon both full and partial analysis, the correlation to mutation density remains non-significant (S5 Table).

### Gene size is not a predictor of mutational density

While at first sight these results provide no support for the adaptive hypotheses, we were concerned by the strength of the gene size effect (Table 1), not least because a plot of gene size against mutational density of binned data shows no obvious trend (Fig 2A). As there are more genes than mutations, many genes have no mutations. Short genes are expected to be especially prevalent in the set of genes with no mutations, simply because they are short, as indeed we see (S5 and S6 Figs). In between-gene analyses (Table 1), all genes are, however, treated equally. Could it be that having so many short genes with no mutations forces a strong positive correlation between gene size and mutational density?

To consider this possibility, instead of simply applying an off-the-shelf statistical method alone, we considered the behaviour of Kendall’s tau when considering randomly simulated data. To this end, we consider the total number of CDS mutations in our sample and reallocate them at random to the same set of bases (defined by the trinucleotide) as in the candidate set of CDSs. This generates one simulated set of mutational densities that respects the effect of gene size on mutational density. We then consider the correlation between gene size and mutation density in this simulated set of genes. We repeat this 10,000 times to define a distribution of correlation scores that would be expected under a null model in which gene size is not causative of variation in mutational density. As the observed mean correlation statistic (Fig 3A) is almost exactly as strong as the correlation observed in the real data (Table 1), we conclude that the gene size effect seen in the real data using the off-the-shelf statistical method is misleading and can instead be explained entirely as a statistical artefact.

**Fig 3:**
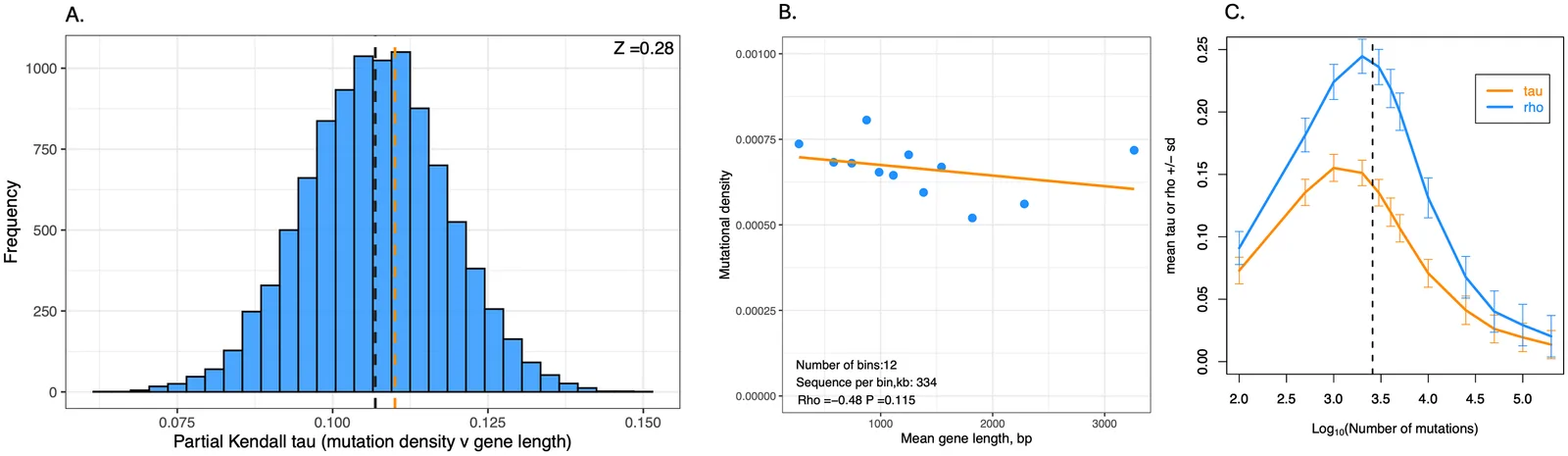
Lack of effect of gene size on mutation density. **A.** Mutation density by gene length for observed and simulated data (partial Kendall correlations). The partial tau values found for each of the 10,000 simulated mutation densities are shown in the blue histograms and their mean by the black line, whilst the value from the observed mutation density by the orange line. The *Z* score displayed in top right represents the deviation in observed to the simulated null calculated by subtracting the mean simulated value from the observed value and dividing it by the standard deviation of the simulated values. **B**. Average mutation density per gene length controlling for total gene sequence. To assess mutational density across gene sizes, genes were ranked by length and divided into bins of approximately constant total sequence length such that for a total sequence of *N* bases and *M* bins, each bin contained approximately *N*/*M* bases, resulting in fewer genes in bins with larger gene sizes. Mutational density was calculated for each bin by dividing the total count of observed mutations by the total sequence length within that bin. To control for potential artefacts arising from arbitrary bin selections, the analysis was repeated using multiple combinations of bin sizes and numbers ranging from 4 to 30 bins (S7 Fig). Here we show a representative plot, selecting a middle bin value. **C.** Simulation of rho or tau value reported between mutational density and gene size when mutations are randomly allocated to genes. To this end, we consider the genes of *E. coli* and ascribe each base pair a unique number. We then randomly select *N* such numbers, this mimicking mutation, and add one to the “mutation” count of the gene associated with each mutation. We divide the count per gene by the gene size to generate the simplest null distribution of mutational densities. We then correlate the densities against gene size using either Spearman rank correlation or Kendall’s tau. For each value of *N*, we repeat the simulation 1,000 times to generate a mean and standard deviation of the reporting statistic (tau or rho). We vary *N* from 100 to 200,000. The *x* axis is the log 10 of *N*. The vertical line indicates the number of mutations in our data set. Note that this simulation avoids complications of gene size by nucleotide content effects and just reflects the consequences of having many genes with zero density. The data and code needed to reproduce this Figure can be found in https://doi.org/10.5281/zenodo.23263169.

We confirm this a second way. Here, we rank-order all genes by gene size and then create bins of genes where the total sequence featuring in each bin is approximately constant. If the total amount of CDS is *N* bases, then if we have *M* bins, all bins contain approximately *N*/*M* bases. Where the bins differ is in the mean sizes and numbers of genes in each bin: there will be fewer genes in the bins of longer genes and the most genes in the bins of the smaller genes. We then ask about the total number of mutations in each bin and divide that by the total sequence length in the bin to derive a mutational density for each bin. If longer genes really do have a higher mutational density, this method should reveal that the bins containing the longer genes should have a higher mutational density, although this doesn’t control for other covariates. To overcome the fact that the number/size of the bins is arbitrary, we repeat the analysis using multiple size/number combinations. In no instance is there a positive correlation (S7 Fig; a representative example shown in Fig 3B). Indeed, if anything, the effect may be negative (bins of longer genes may have slightly lower mutational densities), but the effect is weak and typically not significant (S7 Fig). The negative trend is more commonly significant when Foster data alone is considered (S8 Fig). This strongly supports the hypothesis that the strong positive gene size versus mutational density correlation is an artefact, likely owing to the preponderance of small genes with no mutations (S5A Fig).

We also ask via simulation whether we might expect both Kendall’s tau and Spearman’s rank correlation to mislead and how this might vary with the number of mutations (Methods and Fig 3C). We find that, as we expected, Spearman’s rank correlation is more profoundly affected by the artefact and that in both cases, by unfortunate coincidence, the peak over-estimation occurs when the mutation numbers (*N*) is around the numbers we here employ (a few thousand). The effect nonetheless persists weakly even with 200,000 mutations. It also diminishes at very low numbers as, when we approach the case where only very few genes have any mutations, we approach what in regression would be a flat line (at the limit all genes have the same mutational density of zero). We conclude that prior gene length-mutational density correlations are likely to have been affected by this artefact, not least because rho was the preferred statistic [19].

### No evidence of adaptive mutation rate variation, also when controlling for artefacts

The above questions whether the correlation results (Table 1) are also artefacts of gene size versus mutational density effects seen under null. They also question prior results that employed Spearman rank correlations (e.g., [20]) as we would expect that any variable that covaries with gene size could be affected. To address this, we consider again the 10,000 simulants and again determine the Kendall correlation between each variable and simulated mutational density. We can then characterise the effect in terms of *Z* scores, where we consider the deviation between the observed tau and the mean null simulated tau, divided by the standard deviation of tau in the simulants. A *Z* of zero or close thereto (as seen for the gene size effect: Table 2 and Fig 3A) then implies that the magnitude of the observed correlation is as expected from the null random mutation model. We consider this for each variable in isolation (Table 2 and S9 Fig) and after partial analysis (Table 2; Figs 4 and S10). In the latter, we take each simulated mutational density across our genes and perform partial correlation analysis using the above variables as covariates. For each simulation, we extract the partial tau and then derive the deviation between the observed partial tau and the mean simulated partial tau and thence a *Z* score with *P* inferred from *Z*.

**Table 2:** Kendall correlation and partial correlation analysis between mutation density and all annotated gene features, for observed and simulated data. “observed_cor” refers to the full Kendall’s tau between observed mutation density and each feature, whilst “observed_pcor” refers to the Kendall partial tau. The *P*-values for both are represented with stars such that *P* < 0.0001 **** *P* < 0.001 *** *P* < 0.01 ** *P* < 0.05 * *P* > 0.05 no stars. “sim_mean_cor” and “sim_mean_pcor” respectively represent the mean of full and partial Kendall’s tau values across 10,000 simulations of mutational density. For each, standard deviations are also included. “Zscore_cor” and “Zscore_pcor” represent the deviation in observed tau to the simulated null calculated by subtracting the mean simulated tau from the observed tau and dividing it by the standard deviation of the simulated tau values (respectively for full and partial tau values). Considered features include gene length, GC content, deviation of CpG content from that expected by mononucleotide content (“CpG deviation”), relative position from origin of replication (“relative position”), gene essentiality classification from the PEC database [77] (“nonessentiality”, scored as 2 for non-essential and 1 for essential), strand orientation relative to the origin (“strand”, scored as 0 for lagging strand, 1 for leading strand), protein abundance from PaxDB [78] (“protein”), and median transcript abundance from a compendium of RNA-seq data [79] (“RNA”). Mutation data was merged from Foster and colleagues [64] (annotated to genome accession NC_000913.2) and Wei and colleagues [65] (NC_000913.3). For GC content, CpG deviation and relative position from the origin, weighted means across the two genomes were used taking into account the proportion of CDS mutations contributed by each data set to the merged CDS mutation data (Foster: 0.65, Wei: 0.35). For simulated data *P*-value is inferred from the *Z* score via R’s pnorm function under two-tailed assumptions and also included in the table such that *P* < 0.0001 **** *P* < 0.001 *** *P* < 0.01 ** *P* < 0.05 * *P* > 0.05 no stars. The data and code needed to reproduce this Table can be found in https://doi.org/10.5281/zenodo.23263169.

feature	observed_cor	observed_pcor	sim_mean_cor(±sd)	sim_mean_pcor(±sd)	Zscore_cor	Zscore_pcor
gene length	0.1271****	0.11****	0.1262 ± 0.0113	0.1069 ± 0.0114	0.0806	0.2784
GC content	0.0265*	−0.0046	0.0596 ± 0.0113	0.0266 ± 0.0114	−2.9319**	−2.7375**
CpG deviation	0.0231*	0.0185	0.0208 ± 0.0115	0.0056 ± 0.0118	0.2	1.0872
relative position	−0.0045	−0.0045	−0.0046 ± 0.0111	−0.0002 ± 0.0114	0.0063	−0.3697
protein	−0.0038	0.0211*	0.0124 ± 0.0114	0.0118 ± 0.0101	−1.4203	0.9213
RNA	−0.0644****	−0.0582****	−0.0147 ± 0.0113	−0.0169 ± 0.01	−4.4014****	−4.1334****
strand	0.0128	0.0036	0.0081 ± 0.0135	0.0013 ± 0.0141	0.3444	0.1666
nonessentiality	0.0069	−1.00E − 04	−0.0085 ± 0.0138	−0.0043 ± 0.0139	1.1123	0.3044

**Fig 4:**
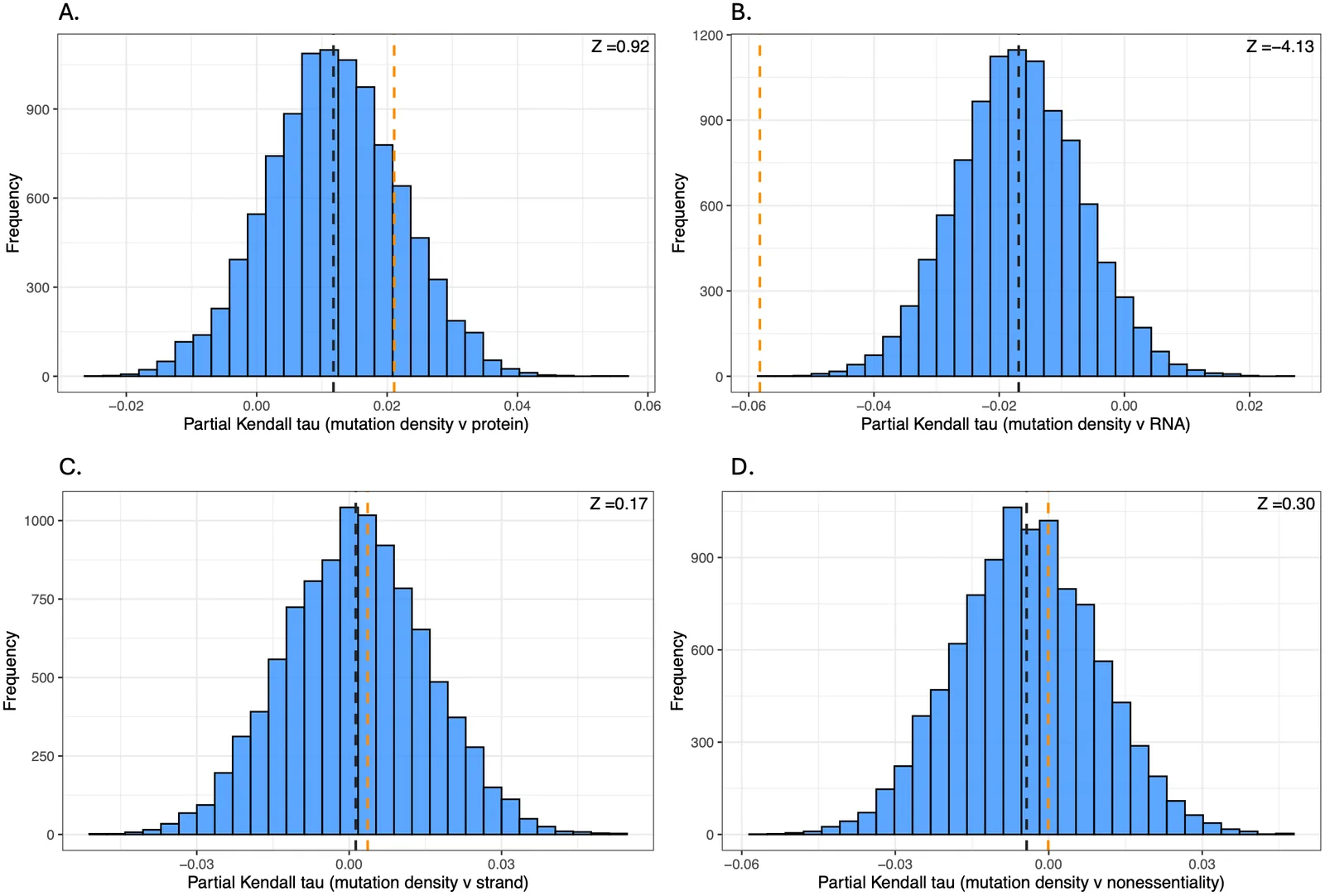
Mutation density by gene feature for observed and simulated data (partial Kendall correlations). Correlations determined between mutation density and gene features suggested to be “adaptive”. Features include protein abundance from PaxDB [78] **(A)**, median transcript abundance from a compendium of RNA-seq data [79] **(B)**, strand orientation relative to the origin (**C**, scored as 0 for lagging strand, 1 for leading strand), gene essentiality classification from the PEC database [77] (**D**, therein scored as 2 for non-essential and 1 for essential). For all panels, the values found for each of the 10,000 simulated mutation densities are shown in the blue histograms and their mean by the black line, whilst the value from the observed mutation density by the orange line. The *Z* score displayed at the top right of each plot represents the deviation of observed from the simulated null calculated by subtracting the mean simulated value from the observed value and dividing it by the standard deviation of the simulated values. The data and code needed to reproduce this Figure can be found in https://doi.org/10.5281/zenodo.23263169.

We find that the RNA transcript level observed tau is significantly more negative than that expected under null (Table 2 and S9 Fig), confirming that this is indeed a predictor: genes with more abundant transcripts tend to have a lower mutational density, even allowing for gene size versus transcript abundance effects. The protein level effect disappears (Table 2 and Fig 4A). We also observe that the reported positive correlation between GC parameters and mutational density (Table 1) is lower than expected under the null (Table 2 and S9 Fig). The strength of the GC effect thus appears to be an artefact of GC versus gene size effects. None of the adaptive parameters are significant before this control (Table 1) or on simulated *Z* scores (Table 2 and S9 Fig). In considering Foster data only, we make the same observations (S6 Table). When we restrict the data to the proteomics and transcriptomics from LB conditions, the full correlation analyses present the same qualitative conclusions, but none of the correlations remain significant in the partial correlation simulations (S7 Table).

As in the simulation approach, the correlation method is used to simply define the null and its distribution, *Z* being the important result, the trends then should be largely independent of the method to calculate the correlations. To determine whether this is true, we re-ran the focal analysis but this time using Spearman’s rho as the correlation method. As expected, the *Z* calculations recovered the same results as above, i.e., significance of the RNA level on full and partial correlation (S8 Table). We conclude that the simulation approach is a largely assumption-free, robust methodology.

These results indicate that the lack of effect seen for the three “adaptive” variables (Table 1) isn’t an artefact of gene length covariation. However, as a correlation analysis is not the most appropriate test for nominal data, we consider an alternative approach for strand and essentiality. We find the mean difference in mutation density between one group and the other, i.e., lagging v leading strand or essential v non-essential genes and compare to simulated expectations. This test confirms no significant deviation from null simulations, both when considering merged (S9 Table and S11 Fig) or only Foster data (S10 Table).

Since RNA-level gene expression is the only significant predictor (Table 2), and since essential genes and leading strand genes tend to be highly expressed (Table 1), we further consider a null simulation in which we allow for mutation by RNA-level effects. For this, we repeat the null simulations but accept a randomly allocated mutation conditional on the RNA expression level of the gene containing the mutation. If the gene is in the least expressed class, we allow the mutation into the simulated set. Otherwise, we include the mutation conditional on the relative mutational density, compared to the least expressed genes, of genes with that expression level. These simulants replicate the mutation density versus gene expression correlation (observed Kendall’s tau = −0.064; mean simulated Kendall’s tau = −0.058 ± 0.0114 standard deviation).

We can then ask, controlling for gene size statistical issues and RNA-level versus mutation density covariation, whether genes on the leading strand or essential genes have lower mutation rates than expected by null. We find that they do not (S11 Table). If anything, trends may be weakly in the opposite direction. When considering the difference in mutation densities between leading and lagging strand in essential genes, we see a significant deviation suggesting higher mutation densities on leading strands (S12C Fig, *Z* = 2.01, two-tailed normalised *P*-value = 0.046, not significant on multi-test correction). We also see this when considering Foster data only (S12 Table, *Z* = 2.91, two-tailed normalised *P*-value = 0.004), as well as a significant difference between essential and non-essential leading strand genes, with higher mutation density in essential genes (S12 Table, *Z* = 2.78, two-tailed normalised *P*-value = 0.006). As regards protein expression, similarly to the simulations controlling for gene size above (Table 2), we see no significant deviation from null in full or partial correlation analysis when considering this new set of simulants controlling for RNA levels as well (S13 Table). We thus confirm that there is no protection from mutation of more important (essential/high protein level) or leading strand genes, even when controlling for gene size or expression effects.

### Further indication that higher transcription is anti-mutagenic in *E. coli*

The negative relationship between transcript level and the mutational density need not reflect mutational effects related to transcription, as variation in RNA decay rates also contributes to mRNA levels. To address this, we consider the association between mutation density and the underlying trends in transcription propensity across the *E. coli* genome. By using a reporter insertion data set of over 144,000 reporters [81], we calculate an average value for RNA per DNA levels ± 1000 bp from the midpoint of each gene. We find transcriptional propensity to be, as expected, positively correlated to transcript abundance (S13A Fig: Kendall’s tau = 0.12, *P*-value <2 × 10^−16^) and negatively correlated to mutational density (S13B Fig: Kendall’s tau = −0.03, *P*-value = 0.048), and for this to be significant when considering deviation from random simulated mutation densities (S13C Fig: Z-score = −3.67, *P*-value = 0.0008). This is consistent with transcription-associated repair mechanisms [82] reducing the mutational load in highly expressed regions, and contrary to models suggesting transcription is mutagenic in WT *E. coli* [19,45,47,62,83].

### Analysis of data from MMR-deficient lines provides qualitatively different results

Some prior attempts to test adaptive hypotheses have relied on data from MMR knockout lines. Indeed, the prior claim [1], that we replicate above, that higher transcription is antimutagenic, was argued to not be robust following analysis of mutations in MMR-deficient lines which did not replicate the effect seen when analysing population-derived rare synonymous SNPs [19]. Might employment of MMR-deficient strains have affected results?

To address this, we rerun all the above analysis, but this time employ a merged set of mutations from MMR-deficient strains [64,65]. We firstly report a positive correlation between gene size and mutational density, though it is not as strong an effect as in WT (Tables 3 and 4). Given the significantly larger set of mutation data from MMR-deficient lines compared to WT (see S14 Fig for MMR knockout mutation data summary and S14 Table for mutation annotations per gene), we find it unlikely for this correlation to be an artefact owing to a preponderance of short genes with no mutations, this supported by observing deviation from a correlation expected by random simulations (Table 4) and by testing correlations after binning genes based on their length (S15 Fig). This suggests real differences between WT and MMR-deficient lines in the profile of mutations.

**Table 3:** Full (top) and partial (bottom) Kendall correlation analysis between mutation density and all annotated gene features, for MMR-deficient strains. Mutation density (“mut_density”) determined as mutation count per gene normalised by gene length. Features include gene length (“gene_len”), GC content (“GC”), deviation of CpG content from that expected by mononucleotide content (“CpG_dev”), relative position from origin (“rel_pos”), gene essentiality classification from the PEC database [77] (“nonessentiality”, scored as 2 for non-essential and 1 for essential), strand orientation relative to the origin (“strand”, scored as 0 for lagging strand, 1 for leading strand), protein abundance from PaxDB [78] (“prot_abundance”), and median transcript abundance from a compendium of RNA-seq data [79] (“RNA_abundance”). Mutation data combined from Foster and colleagues [64] and Wei and colleagues [65] for a total of 74,007 CDS mutations annotated respectively to the reference *E. coli* genomes NC_000913.2 and NC_000913.3. For GC content, CpG deviation, gene length and relative position from the origin, weighted means were used taking into account the proportion of CDS mutations contributed by each data set to the merged CDS mutation data (Foster: 0.08, Wei: 0.92). Stars represent *P*-value (*P* < 0.0001 **** *P* < 0.001 *** *P* < 0.01 ** *P* < 0.05 * *P* > 0.05 no stars). In bold those tests in which correlation direction and/or significance at *P* < 0.05 differs between WT and MMR-deficient strains. The data and code needed to reproduce this Table can be found in https://doi.org/10.5281/zenodo.23263169.

	mut_density	gene_len	GC	CpG_dev	rel_pos	nonessentiality	strand	prot_abundance	RNA_abundance
**mut_density**		0.054****	0.132****	−**0.006**	0.021*	0.029*	0.021	−0.006	**0.012**
**gene_len**	0.025*		0.230****	0.092****	−0.013	−0.027*	0.029*	0.040***	−0.070****
**GC**	0.126****	0.201****		0.124****	−0.068****	−0.058****	0.054****	0.064****	0.053****
**CpG_dev**	−0.02	0.063****	0.095****		−0.015	−0.096****	0.030*	0.159****	0.104****
**rel_pos**	0.025*	0.002	−0.063****	0		0.064****	−0.013	−0.029**	−0.039***
**nonessentiality**	0.038***	−0.029**	−0.027*	−0.052****	0.051****		−0.077****	−0.211****	−0.264****
**strand**	0.02	0.005	0.040***	0.018	−0.006	−0.064****		0.043***	0.039**
**prot_abundance**	**−0.017**	0.074****	0.013	0.107****	−0.004	−0.078****	0.021		0.507****
**RNA_abundance**	0.025*	−0.132****	0.029**	0.026*	−0.016	−0.187****	0.004	0.478****	

**Table 4:** Kendall correlation and partial correlation analysis between mutation density and all annotated gene features, for observed and simulated data from MMR-deficient strains. “observed_cor” refers to the full Kendall’s tau between observed mutation density and each feature, whilst “observed_pcor” refers to the Kendall partial tau. The *P*-values for both are represented with stars such that *P* < 0.0001 **** *P* < 0.001 *** *P* < 0.01 ** *P* < 0.05 * *P* > 0.05 no stars. “sim_mean_cor” and “sim_mean_pcor” respectively represent the mean of full and partial Kendall’s tau values across 10,000 simulations of mutational density. For each, standard deviations are also included. “Zscore_cor” and “Zscore_pcor” represent the deviation in observed tau to the simulated null calculated by subtracting the mean simulated tau from the observed tau and dividing it by the standard deviation of the simulated tau values (respectively for full and partial tau values). Considered features include gene length, GC content, deviation of CpG content from that expected by mononucleotide content (“CpG deviation”), relative position from origin of replication (“relative position”), gene essentiality classification from the PEC database [77] (“nonessentiality”, scored as 2 for non-essential and 1 for essential), strand orientation relative to the origin (“strand”, scored as 0 for lagging strand, 1 for leading strand), protein abundance from PaxDB [78] (“protein”), median transcript abundance from a compendium of RNA-seq data [79] (“RNA”). Mutation data was merged from Foster and colleagues [64] (annotated to genome accession NC_000913.2) and Wei and colleagues [65] (NC_000913.3). For GC content, CpG deviation and relative position from the origin, weighted means across the two genomes were used taking into account the proportion of CDS mutations contributed by each data set to the merged CDS mutation data (Foster: 0.08, Wei: 0.92). For simulated data *P*-value is inferred from the *Z* score via R’s pnorm function under two-tailed assumptions and also included in the table such that *P* < 0.0001 **** *P* < 0.001 *** *P* < 0.01 ** *P* < 0.05 * *P* > 0.05 no stars. In bold, those tests in which correlation or *Z* score direction and/or significance at *P* < 0.05 differs between WT and MMR-deficient strains. The data and code needed to reproduce this Table can be found in https://doi.org/10.5281/zenodo.23263169.

feature	observed_cor	observed_pcor	sim_mean_cor(±sd)	sim_mean_pcor(±sd)	Zscore_cor	Zscore_pcor
gene length	0.0539****	0.0246**	0.0192 ± 0.0112	0.0164 ± 0.0109	3.1045**	0.7557
GC content	0.1324****	0.1257****	0.0078 ± 0.0107	0.0025 ± 0.0106	11.6028****	11.6396****
CpG deviation	**−0.0059**	−0.0204	0.0028 ± 0.0111	0.0006 ± 0.0112	**−0.7836**	**−1.8787**
relative position	0.0214*	0.0254*	−0.0007 ± 0.0101	0 ± 0.0104	2.18*	2.4448*
protein	−0.0063	**−0.0168**	0.0018 ± 0.0110	0.0014 ± 0.0099	−0.7251	−1.8448
RNA	**0.012**	0.0247*	−0.0013 ± 0.0107	−0.0015 ± 0.0095	**1.2476**	2.757**
strand	0.0212	0.0204	0.001 ± 0.0128	0 ± 0.0131	1.5874	1.5575
nonessentiality	0.0286*	0.0382***	−0.001 ± 0.0127	−0.0002 ± 0.0127	2.3387*	3.0295**

Importantly, we cannot replicate the significant negative correlation between transcript abundance and mutational density, as we observe a positive significant correlation when considering the merged data (Tables 3, 4) and a negative but not significant one in MMR KO Foster data only (S15 Table). The significant positive correlation is also seen when considering transcriptional propensity (S16 Fig). Thus, the prior rejection of the hypothesis that more transcribed genes have a lower mutation rate could be an artefact of choice of mutational data. Additionally, we find GC content to be the strongest predictor (Table 3). To some degree, this is expected as, while the mutation bias reverses (GC → AT in WT, AT → GC in MMR-deficient strains [58]), the more mutagenic A/T residues tend to be those flanked by G/C (S17 Fig and also suggested in Foster and colleagues [84]).

As regards the adaptive mutation claims, MMR knockout data supports a lower mutation rate in essential genes (Tables 3 and 4). It also supports variation with respect to the origin-terminus location, especially so if considering a more sinusoidal function peaking at the midpoints between origin and terminus (S16 Table).

### Results are not contingent on inclusion of non-MMR modified knockout lines

In our analysis of WT data, we followed Foster in employing non-MMR KO lines with the same mutation rate and spectra as WT, assuming them to be WT equivalents. This is not a trivial assumption not least because a high proportion of the Foster data that we treated as effectively WT are deficient in other DNA repair pathways. Given that we observe qualitatively different results for MMR-deficient lines, we should ask whether the decision to use all of Foster’s non-MMR KO lines with the same mutation rate was a safe assumption. We find the inclusion of all Foster data defendable and to not affect results.

We first sought to confirm that the mutational spectra seen in the non-MMR KO lines did, as claimed [64], reflect the mutational spectrum seen in WT lines. To provide better resolution of WT data, we augmented the data set with three smaller samples, samples that were unfortunately too small to test for an absence of selection. In addition to Foster’s PFM2 normal sample (*N* = 192 mutations in CDS) and Wei WT data (*N* = 901), we augment the mutation data set with that from Sane and colleagues [67] (*N* = 188), Long and colleagues [66] (*N* = 55) and Tincher and colleagues [68] (*N* = 63), for a total of 1,399 CDS mutations from “pure” WT *E. coli* strains (S18A Fig). While we cannot guarantee an absence of selection in each data set, the sample as a whole is tolerable, passing the by-site tests and stop codon depletion test, although Ka/Ks is low and on the edge of significance (S18 Fig). The mutation profile of the rest of Foster’s samples (i.e., not WT and non-MMR knockouts) correlates well from this “pure” WT set (Fig 5A: Kendall’s tau = 0.56, *P*-value = 8 × 10^−11^; Spearman rho = 0.75). A large part of the reason that this isn’t a higher figure is because of the sample size-associated noise. From bootstrap sampling of the WT data to produce a data set of the size of the Foster non-MMR, non-WT data (*N* = 1,342), the expected tau is on average 0.64, this being the correlation of WT observed against the same WT data sampled with replacement and representing a mean upper bound.

**Fig 5:**
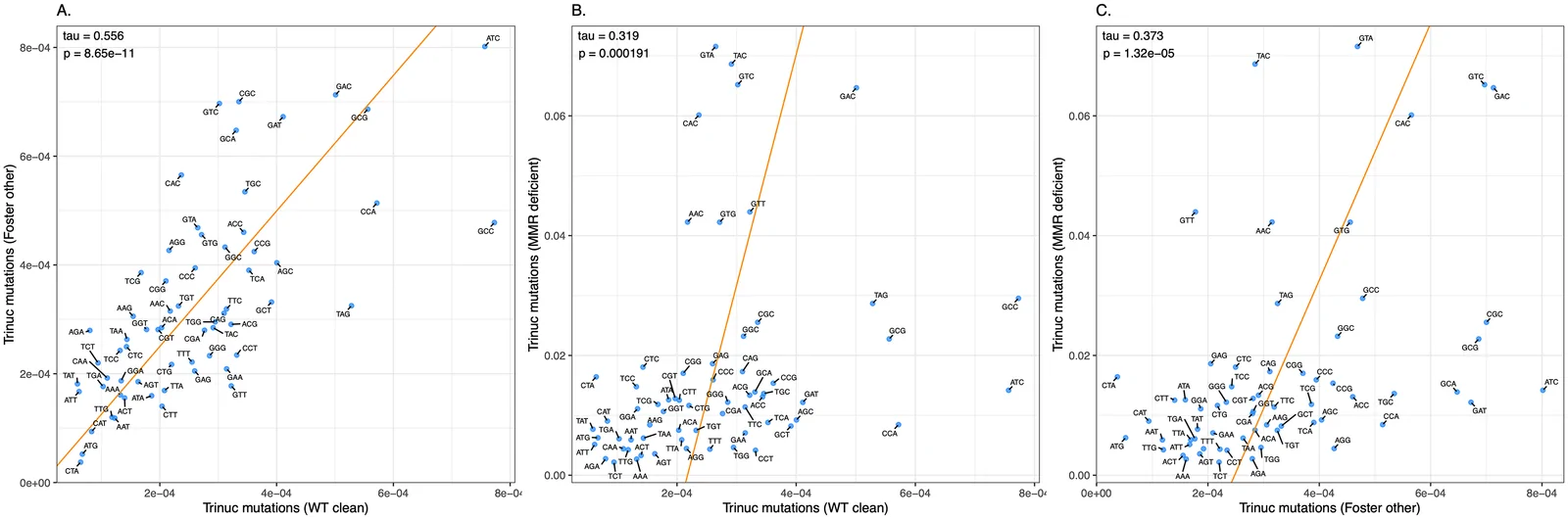
Mutation profiles in “pure” WT compared to mutant lines. Scatter plots of trinucleotide mutations rates for (**A**) an assemblage of strictly WT samples from Foster and colleagues [64], Wei and colleagues [65], Sane and colleagues [67], Long and colleagues [66] and Tincher and colleagues [68] compared to that of other mutants from Foster data with equivalent mutation rate as WT, and (**B**) the same WT assemblage compared with that from MMR-deficient lines from Foster and Wei data. (**C**) that from MMR-deficient lines from Foster and Wei data against the Foster data with equivalent mutation rate as WT. For all panels, line represents orthogonal regression between the two data sets, and a Kendall’s tau and the respective *P*-value are displayed in each plot. Trinucleotides were formed such that the middle focal base is the mutating one and can occur at any codon site. Trinucleotide mutations are determined per occurrence of the trinucleotide in the respective protein-coding genome: for Foster and colleagues data genome accession NC_000913.2 and for all others NC_000913.3. The data and code needed to reproduce this Figure can be found in https://doi.org/10.5281/zenodo.23263169.

While then the Foster non-MMR, non-WT data is near this viable upper bound, by contrast, when comparing the mutation profile of MMR-deficient lines to the WT set, the correlation is much reduced (Fig 5B: Kendall’s tau = 0.32, *P*-value = 0.0002) with numerous odd outliers distorting the orthogonal regression line (Fig 5B). To ask whether the correlations (MMR v WT, Foster non-MMR KOs v WT) are different allowing for sample size differences, we performed a subsampling test. With 1342 mutations in the Foster non-MMR non-WT data, we randomly sampled this number from the MMR data set and recalculated per trinucleotide mutational rates. We correlated per trinucleotide rates for each subsample against the rates seen in WT data. In no case out of 10,000 simulations was the correlation for the MMR data as strong as that seen for the Foster non-MMR KO data (mean tau in simulations = 0.33, ± 0.032 sd), hence *P* < 0.001 [85]. We conclude that the trinucleotide mutation rates of Foster non-MMR KO data significantly better resembles the WT data than does the MMR knockout data allowing for sample size differences. As expected, then, the Foster non-MMR KO data is about as well correlated with the MMR KO data as the latter is correlated with the WT data (Fig 5C).

On these grounds, there is a case that inclusion of WT equivalents will not have misled us. The core question, however, is whether analysis of the pure WT set reports the same predictors of mutation density as above. We find that performing a full and partial correlation analysis on this “pure” set shows no qualitative difference in the results (S17 Table). As expected, the gene length artefact is still present (S17 Table) and is removed when considering the simulated data (S19 Fig). The next strongest predictor of mutation density is transcript abundance (S17 Table: full Kendall’s tau = −0.048, partial Kendall’s tau = −0.036, both significant), this being the only factor that remains significant upon simulation testing (S19 Fig). We again see no effect of strand, protein abundance or essentiality (S17 Table and S19 Fig). Results are unaffected by considering expression data from LB growth conditions alone (S18 Table), except that transcript abundance correlates significantly with mutation density upon full but not partial correlation analysis. We conclude results are not dependent on inclusion of mutation data from *E. coli* lines with modified DNA repair pathways other than MMR.

### No evidence for a strand effect in other bacteria

How do our findings in *E. coli* compare to other bacteria? Unfortunately, no other species has good proteome and systematic knockout data aligned with a good number of well-called mutations, preventing replica analyses. There are, however, three bacterial genomes with relatively good mutational coverage, these being *Mycobacterium smegmatis*, *Streptococcus pneumoniae*, and *Lactobacillus acidophilus* [86]. For the latter, MA experiments were carried out [87] using a genome that is not freely available, and the remaining two species lack high-coverage protein expression data (it being available for only a few hundred genes or for another strain [78]). While not then able to perform replica analyses, we can nonetheless perform a more limited analysis on between-gene variation in mutation density to test the hypothesis that genes on the lagging strand have a higher mutation rate.

We employ MA data for *S. pneumoniae D39* from Jiang and colleagues (total CDS mutations 1,321) and for *M. smegmatis MC2 155* data was merged from Kucukyildirim and colleagues [88] and Castañeda-García and colleagues [89] (757 total CDS mutations). As expected, for both species the gene length artefact is found, remaining the strongest predictor of mutation density (S1A and S2A Data), the observed correlation between density and gene length not deviating significantly from the null simulated expected in *S. pneumoniae* (S20A Fig: observed Kendall’s tau = 0.1178, median simulated Kendall’s tau = 0.1362, *Z* = −1.14) and, although it does in *M. smegmatis*, the observed is weaker than expected (S21A Fig: observed Kendall’s tau = 0.0741, median simulated Kendall’s tau = 0.1075, *Z* = −3.15). Removing gene length as a covariate, we find that strand orientation still does not correlate with mutation density in standard correlation analysis (S1B and S2B Data) nor on pairwise difference analysis (S22A and S22B Fig) and we see no significant difference in mutation densities in leading and lagging strand genes when simulating mutational allocation (S22C and S22D Fig), consistent with our *E. coli* results.

There is sparse transcriptional data that can be added to the analysis. We see no significant correlation between average transcript abundance and mutation density in *S. pneumoniae* (S1B Data: Kendall’s tau = 0.006, *P*-value = 0.730), while we do observe a (marginally) significant weak effect upon partial correlation analysis in *M. smegmatis,* with more highly transcribed genes bearing fewer mutations, as seen in *E. coli* (S2B Data: Kendall’s partial tau = −0.0189, *P*-value = 4.78 × 10^−2^). For both species the RNA effects disappear when comparing to null simulated correlations (S20F and S21F Figs). For *S. pneumoniae* only, mutation density significantly correlates with GC content (S1 Data and S20B Fig).

We conclude that we again fail to detect a difference in mutational profile for genes on different strands and any transcriptionally related effects are weak or non-existent, although higher-quality expression data (transcriptomic and proteomic), as well as knockout data, for these species would be desirable.

### Analysis in yeast reinforces the artefactual gene length effect and the lack of evidence for adaptive mutation rate variation

As a species with large effective population size (*N*_e_), coupled with good quality mutation and expression, for which adaptive claims as regards mutation rates have been suggested, we next consider between-gene variation in mutation rates in the yeast *S. cerevisiae*. We employ mutation data across four sources of WT MA lines [90–93], producing a collection of 4,600 mutations (3,404 of which annotated to the protein-coding sequence). We also employ transcript [94] and protein [78] abundance, as well as gene essentiality [95] and replication timing data [96].

We start, as above, by checking that the mutation data represents results in the absence of selection. We perform the same Ka/Ks test and find that the observed Ka/Ks ratio does not significantly deviate from the null (S23A Fig: *Z*-score = −0.058, *P*-value = 0.954). Upon checking the number of nonsense mutations, we notice that, as with *E. coli*, the observed mutation data set has significantly more nonsense mutations than that expected under null simulation (S23B Fig: *Z*-score = 2.308, *P*-value = 0.021). We conclude we can make the assumption that purifying selection is not importantly operating on *S. cerevisiae* mutation data.

Having established the mutation data is safe to be used, we then consider correlation and partial correlation statistics. This reports a significant correlation between mutation density and transcript or protein abundance (S3 Data: density v RNA full Kendall’s tau = 0.032, *P*-value = 4.13 × 10^−4^, density v protein full Kendall’s tau = 0.050, *P*-value = 9.75 × 10^−8^), much as previously reported for yeast (see [48] though see also [49]). Upon partial correlation analysis, the correlation with transcript abundance reverts to negative as seen with *E. coli* above (S3 Data: partial Kendall’s tau = −0.041, *P*-value = 1.43 × 10^−4^), and protein abundance becomes non-significant (S3 Data: partial Kendall’s tau = 0.004, *P*-value = 0.701). Gene essentiality does not significantly correlate in either case (S3 Data), whilst gene replication timing is significant on both full and partial correlation analysis with later-replicating genes having higher mutation rates (S3 Data: full Kendall’s tau = 0.032, *P*-value = 1.14 × 10^−3^, partial Kendall’s tau = 0.035, *P*-value = 0.0012), as previously suggested (see, e.g., [34,39]). Again, however, these results are dubious as in both full and partial correlation analysis, we again see the strongest predictor of mutation density by far being gene length (S3 Data: full Kendall’s tau = 0.177, *P*-value = 1.008 × 10^−82^, partial Kendall’s tau = 0.103, *P*-value = 9.72 × 10^−22^), suggesting the same potential artefact as described above for *E. coli*.

To allow for the artefact, we thus repeat our analysis generating Monte Carlo simulations comparing the observed Kendall’s tau values to those predicted by null. Upon full correlations (Figs 6 and S24), we see that the gene length effect (again) disappears (S24A Fig: observed Kendall’s tau = 0.176, median simulated Kendall’s tau = 0.1851, *Z* = −1.037) and that the observed correlation with transcript abundance is significantly stronger than that expected under null (Figs 6A and S24D: observed Kendall’s tau = 0.032, median simulated Kendall’s tau = 0.013, *Z* = 2.20), protein abundance being in the same direction (Figs 6C and S24E). Note that, if anything this is contra to the adaptive models suggesting that more important genes (more protein) have a higher mutation rate. The correlation between mutation density and replication time remains robust upon simulation testing, again with later-replicating genes having more mutations (Figs 6G and S24G: observed Kendall’s tau = 0.316, median simulated Kendall’s tau = 0.003, *Z* = 3.10). One other feature shows a significant difference from null, this being CpG content deviation from that expected, with genes with more CpG residues bearing lower mutation densities (S24C Fig: observed Kendall’s tau = −0.0362, median simulated Kendall’s tau = −0.0083, *Z* = −3.11). Essentiality is not a predictor (Figs 6E and S24F).

**Fig 6:**
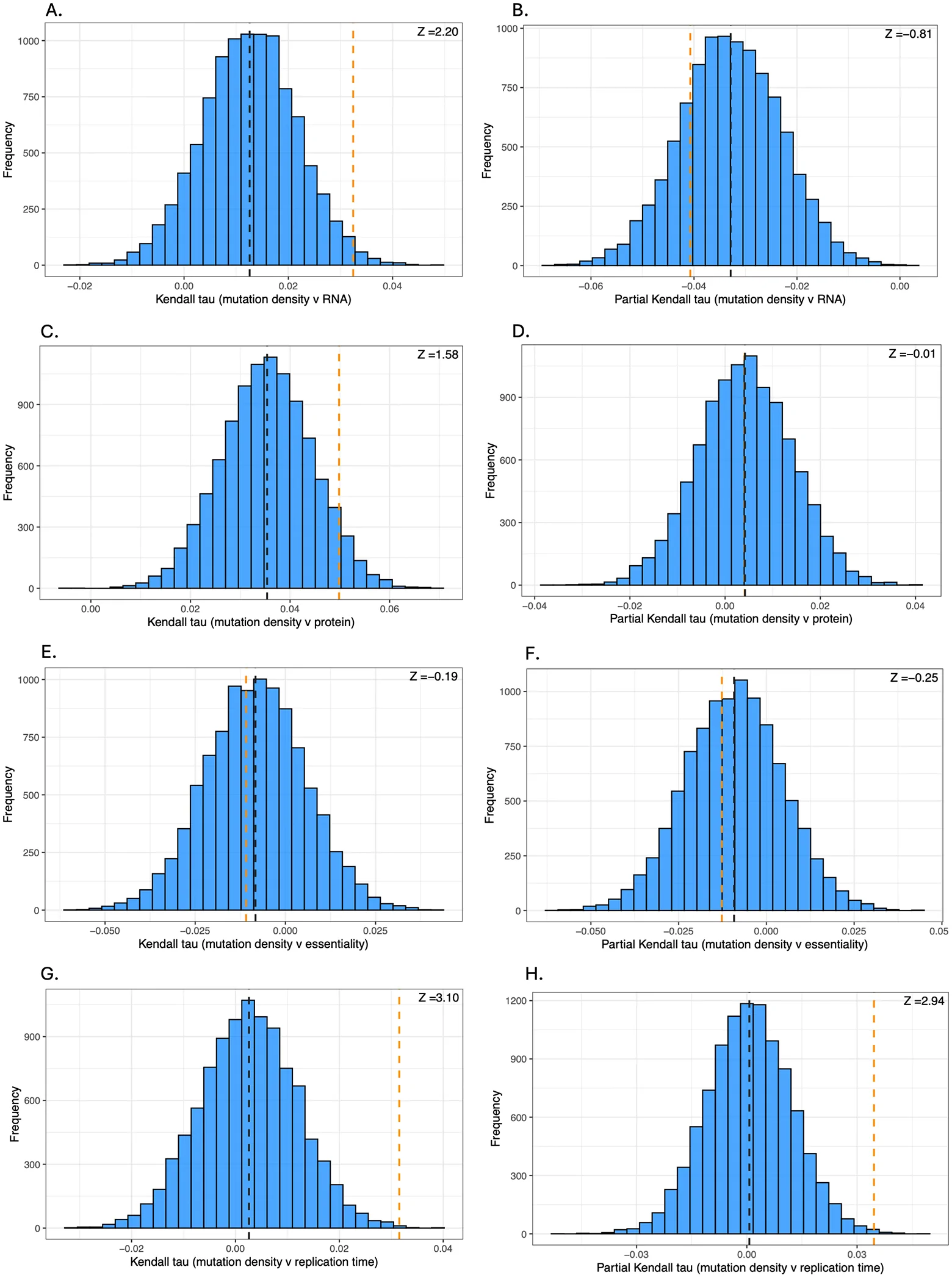
Mutation density by gene feature for observed and simulated data, for *S. cerevisiae.* Correlations determined between mutation density and gene features suggested to be “adaptive”. Features include transcript abundance from the NCBI GEO Database [94] **(A, B)**, protein abundance from PaxDB [78] **(C, D)**, gene essentiality classification from the Yeast Phenome Database [95] (**E, F**, therein scored as 2 for non-essential and 1 for essential), and gene replication timing obtained from Berners-Lee [96] **(G, H)**. As indicated on the *x* axis, the first column shows full Kendall correlations, while the second column is the same but for partial Kendall correlations. For all panels, the values found for each of the 10,000 simulated mutation densities are shown in the blue histograms and their mean by the black line, whilst the value from the observed mutation density by the orange line. The *Z* score displayed at the top right of each plot represents the deviation of observed from the simulated null calculated by subtracting the mean simulated value from the observed value and dividing it by the standard deviation of the simulated values. Mutation data merged from four MA experiments [90–93]. The data and code needed to reproduce this Figure can be found in https://doi.org/10.5281/zenodo.23263169.

Upon simulated partial correlations, we see no significant deviation from null for either transcript or protein abundance, or gene essentiality (Figs 6, S25, and S26). The only features that remain significant are replication timing (Figs 6H and S25G: observed partial Kendall’s tau = 0.0346, median simulated partial Kendall’s tau = 0.0007, *Z* = 2.94) and CpG content deviation from that expected (S25C Fig: observed partial Kendall’s tau = −0.0292, median simulated partial Kendall’s tau = 0.0043, *Z* = −2.77), these being in the same direction as seen for the simulated full correlations. Given this and the significant correlation between transcript abundance and CpG content deviation (S3 Data: Kendall’s tau = −0.124, *P*-value = 2.48 × 10^−51^), we consider the effect of RNA v density (S3 Data) could be an artefact. We conclude that our simulation analysis in yeast reinforces the problem of the artefactual gene length effect and supports that there is no lower mutational density in genes specifying more abundant or more essential proteins.

### Ka/Ks also does not predict mutational density

In neither *E. coli* nor in *S. cerevisiae* do we then see evidence that either essential genes or genes for more abundant proteins have lower mutation rates, and thus we cannot robustly support the hypothesis that selection on a gene’s mutation rate is mediated by its “importance”. While essentiality and expression level have been previously deployed as indicators of importance, the underlying motivation behind such models, however, is that genes subject to stronger, or more common, purifying selection should be those with lower mutation rates while those required for rapid adaptive evolution should have a higher mutation rate. Both genes for more abundant or essential proteins are likely to be genes under common purifying selection, and, as expected, both higher expression level and greater essentiality predict lower Ka/Ks in this data [97], low Ka/Ks being indicative of purifying selection. Models predicting that genes under stronger purifying selection should have lower mutation rates predict a positive correlation between mutational density and Ka/Ks. We see no correlation in either species (*E. coli*: Kendall’s tau = −0.002, *P*-value = 0.935; *S. cerevisiae*: Kendall’s tau = −0.05, *P*-value = 0.67: data from [97]). This argues further against a hypothesis that mutation rate variation is predicted by the effects of such mutation rate variation on the rate of deleterious mutation.

## Discussion

Following early conjecture [12], mutation rate variation between genes within the same genome has often been attributed to natural selection crafting variation, either protecting more “important” genes [1,2,13] or mutating those deemed to be necessary for adaptation [3,15]. In the first instance, we tested these hypotheses by combining MA data from Foster and colleagues [64] and Wei and colleagues [65]. The validity of using MA lines to infer underlying mutation rates rests on the assumption that purifying selection is not operating on the mutations, which we find to be a reasonable assumption, albeit with unusual properties in the Wei data (Figs 1 and S3). Revisiting prominent claims that mutation rates are adaptively lower in more important genes [1,2] (essential genes or those for abundant products) or higher in lagging strand genes due to replication-transcription conflicts [3,16], from simple correlation and partial correlation analysis we find no evidence that being essential, on the leading strand or having a higher protein abundance are predictive of lower mutation rates.

These simple correlation results, however, come with an important caveat as we also identified a methodological complexity of gene-level analysis. We observed that longer genes appear to have a higher mutational density, but, importantly, Monte Carlo simulations are consistent with this correlation being an artefact (Table 2 and Fig 3A). This artefact may have implications for the interpretation of genomic covariates and for any prior analysis employing per-gene mutation density estimates. Any genomic feature that correlates with gene size (S19 Table) may appear to correlate with mutation rate purely as an artefactual byproduct of this size effect. Indeed, we found that the apparent significant positive correlation between GC content and mutation density is not as strong as the one that would be expected under null simulation (Table 2 and S9B Fig). Also, although we observe a negative partial correlation between GC and mutation density when controlling for other gene features (which significantly deviated from null: Table 2 and S10 Fig), we notice the partial correlation reverts to non-significant and weakly positive when gene size is removed as a covariate (S20 Table). The GC effect may be an artefact of GC versus gene size covariance. The strong but false significance of gene size as a predictor serves as a caution that application of standard statistical methods, as previously applied (e.g., [19,20]), has the potential to mislead in such gene-level analyses [64].

Considering deviation from random simulated correlation values to control for this artefact, the only parameter that remains a significant predictor of mutational density, both before and after partial correlation analysis, is transcript abundance (Table 2; S9F and S10F Figs). We observe a negative correlation, indicating that genes with higher transcript levels have lower mutation densities. This effect is specific to RNA levels and is not seen for protein abundance (S9E and S10E Figs), despite correlation between the two (Table 1). If true, this distinction suggests that the mechanism is linked to the act of transcription itself rather than related to the functional importance of the gene product as assayed by protein level. An antimutagenic effect of transcription is reinforced with analysis of transcriptional propensity data (S13 Fig).

The above conclusion, however, comes with important caveats. First, the transcript level effect, while remaining negative, is non-significant if we employ the expression data from LB media under *Z* scored partial correlation (S7, S18 Tables). This may, however, reflect greater noise in the sparser LB media data. Second, while not controlling for trinucleotide content, as the mutation count per gene (i.e., not normalised by gene size) is over-dispersed (*p* < 0.001, in dispersiontest of AER package), we can apply negative binomial regression (from the MASS package in R) with logged gene size as an offset (a doubling of gene length is expected to enable a doubling in mutation count). There is no evidence that a zero excess model is required (check_zeroinflation from R package performance, *P* > 0.05). We can either log the expression data (log_10_(value +1)) or leave it raw and can apply the expression data from the larger set or the LB-specific set, giving four models (S2 Text). In such models, using the full Foster and Wei mutational data, if we employ the full expression data set, no gene expression parameter is significant, although with unlogged expression data a positive effect of RNA level is almost significant (the trend is negative if logged). When employing the LB-derived gene expression data, there is a weak significant positive effect of protein level, if unlogged, but not if logged (the RNA effect is always negative and not significant). Given this diversity of results, and given too that the Z analysis of the partial correlations when LB-derived data is employed are not significant (S7 and S18 Tables), we conclude only that higher RNA transcript levels *may* be associated with lower mutation rates as supported by our simulations when the full, less noisy, expression data is employed. The “fragility” of these effects accords with the notion that these are not robust predictors and, we suggest, may go some way to explain why the expression-mutation correlation is so remarkably variable in sign in prior analyses (see, e.g., [1,19,45–52]).

Were the trend between expression and mutation positive, it would provide no support for any selectionist models relating gene importance to between-gene mutation rate evolution. Conversely, an antimutagenic effect of transcription in *E. coli* accords with prior evidence showing the repair is focussed on coding sequences, hence intergenic sequences have a higher mutation rate [58]. This makes some sense from a selectionist point of view as selection enabling repair coupled to transcription is likely to be relatively strong as so many genes would be affected. Would a possible negative correlation additionally support the hypothesis that between-gene variation in the mutation is also explained by the action of selection?

One prior objection [19] to the claim of lower mutation rates in highly transcribed genes [1] used MMR knockout lines and, as we have shown, this is problematic. There are, however, a series of reasons to suppose that a selectionist explanation is relatively unimportant. One objection is theoretical. While the strength of selection to reduce the mutation rate of all genes must be relatively large (as there are many transcribed genes), selection to specifically lower the rate of just few key genes is by contrast weak [19]. A related second problem, that we discuss more below, and reflected in the fragility of the sign of results, is that the effect size is so miniscule that any adaptationist explanation appears to explain only a trivial amount of between-gene variation. By contrast, intergenic sequences have mutation rates plainly higher than genic sequences ([28]), indeed, not controlling for nucleotide content differences, we estimate that non-CDS in *E. coli* has a per-base mutation rate about 2–3 times higher than CDS, an unsubtle effect. Further, in *E. coli* if we suppose that the net effect of transcription-associated mutation and transcription-associated repair is weakly lower mutation rates in more highly expressed genes, and not a consequence of selection mediated by the abundance of the product, then we can understand why higher RNA level does and higher protein level does not predict lower mutation rates. A simple selectionist model would more obviously predict lower mutation rates for genes that produce more abundant proteins, which we don’t see. Finally, in MA lines in yeast, a species in which selection should also be highly efficient, we find either a weak positive correlation, this being seen on simulation analysis or a positive trend but not significant, seen under partial correlation analysis and, with expression data either logged or not, on Poisson regression (S2 Text). Were the transcription-mutation correlation driven by selection, we would expect it to be an unambiguous negative correlation in all species with a large effective population size. That it isn’t suggests the effects are contingent on mechanistic peculiarities, specific to each taxon. For example, bases found in nucleosome minor grooves experience higher mutation rates [98] and prokaryotes don’t have nucleosomes. Also, co-transcriptional RNA folding is suggested to act as an antimutagenic mechanism that directly mitigates transcription-associated mutagenesis in eukaryotes (see, e.g., [99,100]), but with co-transcriptional translation similar effects are not so obviously expected in prokaryotes. More generally, the predictors of the mutation rate appear to relate to architectural features of genomes: in *E. coli* low GC content commonly seems to be predictive of higher mutation rates (Table 2 and S19 Fig) as do later replicating domains in yeast (S25 Fig). The effect in *E. coli* is consistent with regions of low GC having low thermodynamic stability, thus being more prone to being single-stranded, enabling mutation which in turn attracts the error repair system [101]. Indeed, the greater “breathability” of AT-rich sequence could explain why there is both more mutation in AT-rich areas and a GC->AT bias, processes like cytosine deamination being more likely when DNA is breathing [102].

### Are measures of expression adequate?

Our hesitation concerning selectionist claims is then partly derived from the lack of a negative correlation between mutational density and protein level. This, however, comes with the caveat that our measure of protein level may be inadequate. One might object arguing that the protein data is an integrated data set from numerous growth conditions, conditions that don’t reflect the growth media employed in the MA lines. However, as we have shown, in *E. coli* when we restrict proteomic data to that available collected under LB growth conditions [103,104] (as employed by Foster and Wei), the protein level is neither significant before nor after partial analysis (S21 Table), underscoring that high protein level is not a predictor of low mutational density in *E. coli*. Similarly, the transcript level effect is seen if using data from LB conditions (although not on partial correlation on simulation-derived *Z* score S7 and S18 Tables). There remains the further caveat that, experimental conditions aside, the ability to measure protein level may be noisier than the ability to measure RNA level, leading to weaker correlations. Indeed, comparably, the expression versus rate of evolution correlation, for example, is stronger when more accurate RNA measurements are used [70]. It may then be notable that the correlation between gene dispensability and expression level is stronger for RNA level than for protein level (Table 1). Further, if we correlate expression level against the Ka/Ks values for singleton genes (from [97]) the correlation in *E. coli* is slightly stronger using RNA level than using protein level (Ka/Ks v RNA level Kendall’s tau = −0.147, *P*-value = 1.3 × 10^−10^; Ka/Ks v protein level Kendall’s tau = −0.114, *P*-value = 6.7 × 10^−7^). We see, however, the opposite in yeasts (Ka/Ks v RNA level Kendall’s tau = −0.268, *P*-value = 1.7 × 10^−138^; Ka/Ks v protein level Kendall’s tau = −0.337, *P*-value = 2.21 × 10^−271^, but see also [105]). While this suggests noisier protein level measures in *E. coli*, that the correlation with Ka/Ks is stronger for protein in yeast and, if anything, genes for more abundant proteins have a higher mutation rate in yeasts, suggests that failure to confirm adaptive predictions is not obviously a method artefact.

### MMR knockout data should not be employed to test theories of mutation rate evolution in wild types

The hypothesis that in *E. coli*, repair processes are more effectively active in more transcriptionally active genes, is strengthened by the opposite correlation between RNA level and mutation rate when MMR repair is absent (Tables 3 and 4). That in the MMR data we can see a reverse correlation between transcription and mutation holds a cautionary note as regards employment of proxies to the WT mutation rate. Use of circulating SNPs, synonymous substitutions and mutations in MMR-deficient lines have all been employed as proxies to determine “the” mutation profile. As regards the adaptive hypotheses, the profile in question should be that in WT. While circulating SNPs and synonymous substitutions are problematic as they may be affected by purifying selection, and hence inappropriate to employ when considering questions of gene essentiality, the mutational profile seen in MMR knockouts also appears to be unrepresentative. This is not because of the influence of selection, but rather because the coupling between MMR and transcription, amongst other variables, appears to be modified. We acknowledge a caveat in our analysis of MMR-deficient lines: although we find the Ka/Ks value of the merged data to be 1.02 (S27 Fig), this differs significantly from the Ka/Ks value expected from random simulations (S27A Fig: *Z* = −8.17, *P*-value = 3.22 × 10^−16^), and this difference is likely driven by the Wei data (S27C Fig). Our results nonetheless show a clear difference between WT and MMR-deficient conditions. We thus suggest that it is also inappropriate to employ MMR knockout data to test adaptive hypotheses.

### If gene importance matters, effects sizes must be extremely small

Even if the adaptive parameters were significant predictors, whether they are *important* predictors is instead a question about effect sizes. While in WT *E. coli* transcriptionally mediated effects are the only significant predictors of mutational density (S13C Fig), it is striking that transcript abundance or transcriptional propensity explain less than 0.5% of the variation in mutational density (abundance v mutation density Kendall’s tau = −0.06, *R*^2^ = 0.004; propensity v mutation density Kendall’s tau = −0.03, *R*^2^ = 0.0009), leaving most of the between-gene variation unaccounted for. Indeed, even if one supposes that our measure of protein level is error-prone, if we assume that protein level is imperfectly correlated with RNA level, then the magnitude of any protein level—mutational density effect (that we have been unable to detect) is also going to be extremely small. With the two other adaptive parameters (strand and essentiality) showing much weaker trends than the transcription effects, their effect sizes too are miniscule, as also evidenced in the two further bacteria species.

This weakness is underscored by the results of the negative binomial regressions applied to the *E. coli* mutation count data (S2 Text). While specific results are sensitive to the handling of the data and source of the expression data, all agree that the resulting models have almost no accuracy. The Nagelkerke [106] pseudo *R*^2^ (from R package fmsb), an adjustment of the Cox and Snell [107] pseudo *R*^2^ to scale between 0 (no accuracy) and 1 (perfect accuracy), averages just 0.012 for the full parameter model, and 0.0024 for a model in which just strand, essentiality and protein level are permitted as predictors. In yeast, with essentiality and protein level as predictors in the Poisson regression, the comparable Figure is 0.0009 with the protein level never significant (logged or unlogged). In *E. coli*, if protein level is ever significant, it is in a manner in which more protein is associated with more mutation, not less (e.g., S2 Text). In both yeast and *E. coli* neither strand nor essentiality appear as significant predictors using the large amassed mutation data (Foster + Wei mutation data). We note, however, that in the “pure” WT data there can be a very weak significant trend for essential genes to have a lower “mutation” rate in some negative binomial models when expression data is not logged, but given the evidence of Ka/Ks < 1 in this sample, we may just have detected the early purging of mutations in essential genes and so not have recovered a signal of a true lower mutation rate. We reiterate, however, that, whatever the signs of these effects and whatever their statistical significance (this conditioned on mutation numbers and accuracy of measures), as explainers of between-gene variation in the mutation density, the effect sizes are so small as to appear biologically irrelevant.

These miniscule effects contrast with prior claims. Monroe and colleagues [2] for example claimed that essential genes have a mutation rate per bp up to half that of other classes of genes. Even if we take the numbers uncorrected for covariates, we see a much weaker effect (around 3.5%) that isn’t significant on the trinucleotide-controlled simulations (Fig 2H). Likewise, while the strand effect was claimed to be an unsubtle 2.5-fold difference [16], we see a vastly weaker effect (~4%) with, if anything, the lagging strand having a lower rate (Fig 2G), also not significant. The latter discrepancy can be accounted for by the authors [16] considering the protein rate of evolution not the mutation rate. The former effect is likely owing to contaminated data, the effect not replicating with well-called mutations [20,21]. In contrast to the above, we find the difference in yeast between the earliest and latest replicating sets of genes to be of the order of a ~20% to 30% this being in line with a prior estimate from yeast [39] and congruent with what is seen in humans [35] and flies [37] (although Lang and Murray [34] using artificial inserts in yeast report a much stronger effect, with, on average the later replicating sequences having a mutation rate three times that of the earliest). That replication timing is the most significant predictor in yeast is further underscored by Poisson regression, with later replication associated with higher mutation rates (estimate = 0.015 ± 0.004 sem, *p* = 0.0002). While we can then discern known causative factors as regards variation in mutational density, if selection has shaped the mutational landscape, it has left very hard to discern trends. We thus see no good reason to “*challenge the prevailing paradigm that mutation is a directionless force in evolution* [2].”

## Methods

### Data retrieval

For *E. coli,* the combined data set includes 3,634 total mutations, of which 2,577 occurring in protein-coding sequences, retrieved from Wei and colleagues [65] and Foster and colleagues [64] (which we refer to as Wei and Foster data, respectively). For the latter, we considered data from samples PFM2 in regular and minimal media, as well as all strains the authors report to have a mutation rate comparable to that of WT: PFM35, PFM40, PFM88, PFM91, PFM101, PFM133 [64]. Foster data was retrieved from their supplementary material repository (https://hdl.handle.net/2022/20340). We use the same selection method for Wei data, combining mutations from samples L1, M1, S1, L100 and WT replicates (clones C and D as representative of WT) obtained from S2 Table of that paper [65]. See Fig 1A for a data breakdown. All Foster records were annotated to the reference *E. coli str. K-12 substr. MG1655* genome with accession: NC_000913.2, and Wei records to NC_000913.3. Reference genome sequences and annotations were retrieved on 8th January 2026 from RefSeq NCBI [108]. Mutational data at high resolution is also available from duplex sequencing (see, e.g., [109,110]). However, we previously determined that its properties, such as mutational equilibrium predicted GC content, were so discrepant that the data was likely affected by sequencing artefacts known to bias results [28]. We thus chose not to employ it here.

Mutation data was also retrieved from the same sources [64,65] for *E. coli* MMR-deficient strains. For Foster data, we consider that of samples PFM6, PFM61 and PFM94. For Wei, we consider samples L1, M1, L10 and MMR replicates (clones A and B). See S14 Fig for a data breakdown.

Upon testing for robustness in the results with “pure” WT *E. coli* data only, we consider Foster’s sample PFM2 in normal medium and Wei data, as well as data from Sane and colleagues [67] (from their repository https://doi.org/10.5281/zenodo.22764014), Long and colleagues [66] (from their S2 Table) and Tincher and colleagues [68] (from their S2 Table), the latter three annotated to genome NC_000913.3, for a total of 1,399 CDS mutations (see S18 Fig for data set breakdown). As these latter three data sets are too sparse to perform tests of selection, we exclude them from the main analysis. Note too that in the original Sane paper [67], it was stated that the NC_000913.2 was employed, but on scrutiny of the data we determined a perfect match between the designated genes and ancestral sequences provided and the NC_000913.3 build, hence we employ the NC_000913.3 and not NC_000913.2.

The analysis was, where possible, extended to other bacteria species. Mutation data for WT *S. pneumoniae D39* was obtained from Jiang and colleagues (see their S5 Table in S1 File [111]) for 1,321 total CDS mutations. Data for WT *M. smegmatis MC2 155* was merged from Kucukyildirim and colleagues (see their S2 Table [88]) and Castañeda-García and colleagues [89] (data provided by Dr. Esmeralda Cebrián-Sastre and Prof. Jesús Blazquez) for 757 total CDS mutations.

In replicating the analysis for yeast (*S. cerevisiae*), we obtain data for WT lines from four MA experiments: Zhu and colleagues [90] Sharp and colleagues [91], Liu and Zhang [92], and Sui and colleagues [93]. We combine these to produce a collection of 4,600 mutations, 3,404 of which in the protein-coding sequence including introns and exons (see S22 Table for a gene-by-gene record of reference annotations and mutation counts).

### Feature annotation

Mutations were tracked back to the genes in which they occurred by reference to the genome position indicated in the source data and relevant features for these were extracted (see S1 Table for mutation and feature annotations per gene). All gene annotations were retrieved from reference material downloaded from NCBI [108]. We considered basic structural metrics including gene length (in base pair, bp), as well as the relative distance from the origin to take into account replication order. As it has been suggested that mutation rates could show a fluctuating rather than linear pattern as a function of distance from the origin of replication (see, e.g., [42]), we also consider relative distance from the closest midway point between the origin and the terminus. Strand orientation is scored as a binary factor (leading strand as 1, lagging as 0). As for sequence composition, we consider GC content and the deviation of observed CpG content from what is expected by the mononucleotide frequencies (see formula below), both normalised to gene length.


$$\begin{array}{c} \text{CpG deviation}=\;\frac{\frac{\text{CpG count}}{\text{sequence length}}}{\left(\frac{\text{G count}}{\text{sequence length}})*(\frac{\text{C count}}{\text{sequence length}}\right)} \end{array}$$

Gene essentiality information was obtained from the PEC database by Hashimoto and colleagues [77], who score genes as 2 if non-essential and 1 if essential. Genes scored as unknown were considered as missing information. Although we also consider incorporating records from transposon insertion mutant libraries (Tn-seq) for gene essentiality, recent studies have highlighted discrepancies between Tn-seq and single-gene knockout methods [112]. Specifically, they find that false negatives can arise when transposon insertions occur in non-essential sub-genic domains, whilst protein-DNA interactions hindering insertion can lead to false positives [112]. We thus opt to use the PEC knockout database [77] (accessed on 5th December 2025 from their online source available at: https://shigen.nig.ac.jp/ecoli/pec/download.jsp).

#### *E. coli* parameters.

Expression data was also considered. Protein levels are represented by amassed protein abundance recorded in PaxDB [78] (version 6 updated 2025, accessed 21st November 2025 for taxid 511,145 from https://pax-db.org/species/511145) for reference genome NC_000913.3 (we check both genome versions and find that locus tags refer to the same protein id, indicating that protein measures can be used for both Foster and Wei records). RNA measures are drawn from a compendium of several thousand RNA-seq experiments [79], available from their S2 Table. Although the source for transcript abundance does not clarify if missing data represents no information or zero expression, considering RNA expression as mean versus median, and treating missing data as NA versus zero makes very little difference [28]. We thus opt to use median transcription levels with “no data” treated as zero expression.

While the above data base provides extensive expression data, it covers a diversity of experimental conditions (see S3 Table). As expression is known to alter with conditions in a manner that may impact genic mutation rates [7], we also sought to replicate the correlations between mutation density and expression using the original source data for which the claim was made. Martincorena and colleagues [1] employed data from cDNA sequencing of *E. coli*’s transcription grown in LB medium [113], deposited at https://www.ebi.ac.uk/biostudies/arrayexpress/studies/E-MTAB-387. The processed data provides read counts mapped per base pair of the *E. coli* genome (version NC_000913.2). So as to avoid mis-ascribing reads in cases where genes overlap, for every CDS in the genome we defined the locations that were unique to each gene (i.e., not overlapping with any other CDS). We follow Martincorena and colleagues in considering the mean map number for the bases within each gene to determine that gene’s expression level. Read mappings for two parts of the growth phase are provided, early exponential and transition to stationary. We follow Martincorena and colleagues in employing the former.

For Wei data only, we also consider the underlying transcriptional propensity of genomic regions. For each locus, we calculate a transcriptional propensity value using the reporter insertion data set by Scholz and colleagues [81], which includes over 144,000 reporters inserted throughout the *E. coli* genome. We find the average RNA per DNA values of any reporter construct inserted within a 2,000 bp window (±1,000 bp) centred on the gene’s midway point. For each reporter, Scholz and colleagues give two raw RNA per DNA values (see their S7 Table [81]), and we consider their average in our calculation. Transcriptional propensity can only be considered for Wei data as it was found for the *E. coli* genome NC_000913.3.

For all genes, mutation counts were determined such that if a mutation occurs in overlapping genes, it is scored as 1 divided by the number of overlapping genes (e.g., if it occurs at a position where two genes overlap, a count of 0.5 is added to both genes). We find that no mutations occur in positions with more than two genes overlapping.

To combine feature annotations allowing for potential variations between the reference genomes used in the Foster (NC_000913.2) and the Wei (NC_000913.3) data, we computed weighted means for GC content, CpG deviation, gene length and relative position from the origin or from the closest midway point between origin and terminus. Weights were assigned based on the proportion of mutations contributed by each data set to the merged data (Fig 1A, Foster: 0.65, Wei: 0.35).

Further, between-species values of Ka/Ks were derived from analysis of singleton genes in *E. coli* with one to one orthologs across three close relatives (*E. coli, Escherichia fergusonii* and *Salmonella enterica* as outgroup), with lineage-specific rates since the common ancestor with the in-group (i.e., between *E. coli* and *Escherichia fergusonii)*. For method details see [97], data available at: https://doi.org/10.5281/zenodo.15488140.

#### Other bacteria parameters.

For the analysis on additional bacteria species, the reference genome sequence and annotation was also obtained from NCBI [108], for *S. pneumoniae D39* with accession NC_008533.2 and for *M. smegmatis MC2 155* with accession NC_018289.1, both were accessed on 24th April 2026. As for transcript abundance data, for *S. pneumoniae D39* we use that from Jiang and colleagues [111] kindly provided by Dr. Tongtong Lin, and for *M. smegmatis MC2 155* we use average abundance across the growth phase reported by Li and colleagues [114] (see their S2 Table).

#### *S. cerevisiae* parameters.

For the analysis with yeast, we obtain reference genome information for *Saccharomyces cerevisiae S288C* (accession GCF_000146045.2, assembly R64-2-1) from the Saccharomyces Genome Database (SGD) on 29th April 2026 (see S288C_reference_genome_R64-2-1_20150113.tgz available at: http://sgd-archive.yeastgenome.org/sequence/S288C_reference/genome_releases). As some datasets specified solely that S288C was used, we checked for all four MA data sets that what they report as the reference base for a mutation was the reference base in the above genome. For all mutations in all analyses, this was found. For redundant gene entries, i.e., those with the same identifier and overlapping positions, we annotate mutation counts to the longest entry.

Protein expression is represented by amassed protein abundance recorded in PaxDB [78] (version 6 updated 2025, accessed 29th April 2026 for taxid 4932 from https://pax-db.org/species/4932), and transcript levels were obtained on 28th April 2026 from the NCBI GEO database [94] (see https://www.ncbi.nlm.nih.gov/geo/download/?acc=GSM1239961). This was selected as it closely matches growth conditions used in the above experiments.

Replication timing for each gene was obtained from Berners-Lee and colleagues [96] (accessed on 11th May 2026 from https://github.com/rb2065/S_cerevisiae_DNA_rep_model). Their predictive model employs data from Müeller and colleagues [115] for the *S. cerevisiae* record in OriDB [116]. The input origin data in OriDb is from *S. cerevisiae* sacCer1 [116], which corresponds to the SGD record of the *S. cerevisiae S288C* assembly R27-1-1 [117]. Although this is not the assembly for which the remaining data are available, gene identifiers are conserved between the two assemblies, allowing us to use the record. Since Berners-Lee and colleagues [96] provide predicted replication times for each chromosome at kilobase resolution, we derived a replication time for each gene by first interpolating replication times at the single-base-pair level using a linear model (employing reference genome sequence and annotation for S288C_reference_genome_R27-1-1_20031001.tgz from the SGD, accessed 11th May 2026 at http://sgd-archive.yeastgenome.org/sequence/S288C_reference/genome_releases). We then calculated the mean replication time across all base pairs spanned by each gene.

As for gene essentiality, we use data from the Yeast Phenome Database [95] (https://yeastphenome.org/, previously collected by Rivas-Santisteban and colleagues (see [97] and their code repository at: https://doi.org/10.5281/zenodo.15488140). Further, between-species values of Ka/Ks were derived from analysis of single-copy genes in *S. cerevisiae* with one-to-one orthologs across three close relatives (*S. cerevisiae, Saccharomyces paradoxus* and *Saccharomyces mikatae IFO 1815* as outgroup), with lineage-specific rates since the common ancestor with the in-group (i.e., between *S. cerevisiae* and *Saccharomyces paradoxus)*. For method details see [97], data available at: https://doi.org/10.5281/zenodo.15488140.

### Trinucleotide mutation trends

To consider whether selection operates on the mutations reported in the Foster and Wei MA data, we observed trinucleotide mutation patterns. Foster and Wei mutation data were respectively tracked back to *E. coli* reference genomes NC_000913.2 and NC_000913.3 to extract mutation frequencies for each possible trinucleotide (where the middle base represents the mutation). These were determined by finding the number of mutations occurring in each trinucleotide per occurrence of that trinucleotide within CDS. We calculated frequencies for mutations occurring at any codon site, as well as splitting them based on whether the mutation occurred at within-codon site 1, 2 or 3. We then observed trends across sites and across data sets.

### Ka/Ks estimation for MA line mutations

To investigate whether there may have been selection on mutations in MA lines, Foster and Wei mutations were tracked back by location to *E. coli* reference genomes NC_000913.2 and NC_000913.3 respectively, excluding mutations occurring outside of CDSs or those causing premature stop codons (i.e., nonsense mutations). For each genome, we generated reference and mutated sequences by concatenating all valid CDSs (excluding stop codons). This resulted in, for each genome, a multigene reference sequence and a corresponding mutated sequence containing all mutations recorded for that genome from the respective mutation data set. Additionally, we concatenated the reference and mutated sequences from the two genomes to create sequences that could be used to investigate the evolutionary rate of the combined mutation data. As the reference and mutated sequences are inherently aligned, we then used PAML’s CODEML program [118] to calculate pairwise maximum likelihood estimates of the ratio between non-synonymous and synonymous substitution rates (Ka/Ks). The sequences were analysed at the codon level with estimation of equilibrium codon frequencies using the F3X4 model. We assume a single Ka/Ks ratio for all sites (M0 model) and for the evolutionary rate to be constant across sites. This was considered as the estimation for observed mutation data for both genomes as well as the merged set.

To evaluate the statistical significance of the observed Ka/Ks values, we simulated 10,000 mutated sequences to obtain a null distribution of Ka/Ks values. Using the mutations tracked back to the reference genomes, we determined the trinucleotide context of all mutations. For each mutated trinucleotide and its derived allele, we randomly selected one of the same trinucleotides in CDS. Should this result in a nonsense mutation, the process was repeated until a synonymous or non-synonymous mutation was created. As above for the observed data, we concatenated the reference and simulated mutated sequences for each genome version, as well as for the merged data set. We estimated the Ka/Ks ratios for all 10,000 simulations using CODEML [118] (under the same settings) and compared these simulated distributions against the observed Ka/Ks values through *Z* scores. The *Z* is found by subtracting the mean simulated value from the observed and dividing by the standard deviation of the simulated.

Note that this Ka/Ks analysis of MA line mutations is distinct from the analysis of Ka/Ks of genes seen in between-species comparisons where we expect (and observe) strong evidence of purifying selection (Ka/Ks « 1).

### Correlation analysis

To perform correlation analyses, we opted for Kendall’s tau and partial tau, given the number of genes in our data set with the same mutation density. We employ Kendall’s tau b as it is robust to tied data and unaffected by non-normality in the data [76]. For full Kendall correlation analysis, we employ “corr.test” from R package “psych” with no adjustment and no assumption of a normal distribution for tau values. For partial correlation analysis, we use package “ppcor”.

### Monte Carlo simulations

We perform Monte Carlo simulations and define expected Kendall’s tau under a null model to determine if observed correlations are statistical artefacts. The total number of observed mutations was randomly reallocated with replacement across the entire set of possible CDS bases in each genome, assuming an equal probability of mutation per base. This process generated simulated data sets that preserved the original gene features but randomised the specific mutation locations. For each of 10,000 iterations, we calculated the correlation between each gene feature and the simulated mutation density to establish the distribution of coefficients expected by chance. For bivariate features (strand and essentiality), we also calculated the mean difference in mutation density between the two groups (i.e., leading - lagging and essential - non-essential) instead of the correlation.

We then compared simulated values to the observed values by producing a Z-score representative of the deviation between the two. The Z is found by subtracting the mean simulated value from the observed and dividing by the standard deviation of the simulated.

We further considered a null simulation in which we allow for mutation by RNA level effects. For this, we repeated the null simulations but accepted a randomly allocated mutation conditional on the expression level of the gene containing the mutation. If the gene is in the least expressed class, we allowed the mutation into the simulated set. Otherwise, we allowed the mutation in conditional on the relative mutational density, compared to the least expressed genes, of genes with that expression level.

### Simulations to determine strength of artefacts in Spearman and Kendall correlations

To determine the extent to which correlation methods produce artefactually strong correlations, we considered the simplest simulation. Unlike the above simulations where we respect the trinucleotide context of all mutations, here we do not. Rather, we take the set of CDS genes in *E. coli* and ascribe each base a unique identification number, there being a total of *M* such numbers, equal to the number of CDS base pairs (note we here ignore gene-gene overlaps). We then consider *N* mutations. For these, we randomly sample with replacement *N* from the *M* identifiers. We then reattribute the identifiers to their host genes thereby generating a count of the number of mutated bases per gene. We divide this number by the gene length to derive a simulated mutational density. We repeat the simulations 1,000 times for each value and *N* and, in turn, vary *N*.

### Gene binning controlling by total sequence length

To assess mutational density across gene sizes, genes were ranked by length and divided into bins of approximately constant total sequence length such that for a total sequence of *N* bases and *M* bins, each bin contained approximately *N*/*M* bases, resulting in fewer genes in bins with larger gene sizes. Mutational density was calculated for each bin by dividing the total count of observed mutations by the total sequence length within that bin. To control for potential artefacts arising from arbitrary bin selections, the analysis was repeated using multiple combinations of bin sizes and numbers ranging from 4 to 30 bins.

### Statistics

All data handling and statistical tests were carried out in Python 3 and R. All necessary scripts are available at https://doi.org/10.5281/zenodo.23263169.

## Supporting information

S1 Fig Trinucleotide mutation profiles per codon site. Distribution of trinucleotide mutation differences for each pairwise codon site comparison. Shapiro-Wilk normality test results displayed on the plot, as well as those of a sign test with a null of 50:50 positive:negative differences. **A–C** for Foster data; **D–F** for Wei data. Trinucleotides were formed such that the middle focal base is the mutating one and can occur at within-codon site 1, 2, or 3 (as labelled). Trinucleotide mutations determined per occurrence of the trinucleotide in the protein-coding genome. For Foster and colleagues [64], genome accession NC_000913.2 and for Wei and colleagues [65] NC_000913.3. The data and code needed to reproduce this Figure can be found in https://doi.org/10.5281/zenodo.23263169.
(PDF)

S2 Fig Scatter plot of trinucleotide mutations recorded in Foster and Wei data. Trinucleotides were formed such that the middle focal base is the mutating one and irrespective of within-codon position (site 1, 2, or 3). Trinucleotide mutations determined per occurrence of the trinucleotide in the protein-coding genome. For Foster and colleagues [64], genome accession NC_000913.2 and for Wei and colleagues [65] NC_000913.3. The line represents orthogonal regression between the two data set, and the Kendall’s tau and respective *P*-value is displayed on the plot. The data and code needed to reproduce this Figure can be found in https://doi.org/10.5281/zenodo.23263169.
(PDF)

S3 Fig Testing for lack of selection in mutation data. **A–C** Ka/Ks estimation for observed and simulated mutation data. **D–F** Number of nonsense mutations for observed and simulated data. For A and D, mutation data merged from Foster and colleagues [64] (with *E. coli* genome NC_000913.2) and Wei and colleagues [65] (NC_000913.3). B and E include Foster data only, C and F Wei data only. For all panels, the values found for each of the 10,000 simulated mutation densities are shown in the blue histograms and their mean by the black line, whilst the value from the observed mutation density by the orange line. The *Z* score displayed at the top right of each plot represents the deviation of observed from the simulated null calculated by subtracting the mean simulated value from the observed value and dividing it by the standard deviation of the simulated values. The *P*-value displayed is inferred from the *Z* score via R’s pnorm function under a two-tailed assumption. The data and code needed to reproduce this Figure can be found in https://doi.org/10.5281/zenodo.23263169.
(PDF)

S4 Fig Scatter plot of transcript abundance from a merged set of experiments and one in LB conditions. The full set is the median transcript abundance from a compendium of *E. coli* RNA-seq data [79], this compared to transcript abundance obtained from an experiment involving growth in LB medium by Martincorena and colleagues [1]. On both axes, values are transformed by adding 1 and taking its log10. Line represents orthogonal regression. Spearman rho and respective *P*-value is displayed on the plot. The data and code needed to reproduce this Figure can be found in https://doi.org/10.5281/zenodo.23263169.
(PDF)

S5 Fig Average proportion of genes with zero mutations per sequence feature. Features include gene length (**A**), GC content (**B**), deviation of CpG content from that expected by mononucleotide content (**C**), relative position from origin (**D**), protein abundance from PaxDB [78] (**E**), and median transcript abundance from a compendium of RNA-seq data [79] (**F**), strand orientation relative to the origin (**G**, scored as 0 for lagging strand, 1 for leading strand), gene essentiality classification from the PEC database [77] (**H**, therein scored as 2 for non-essential and 1 for essential). For all panels, genes binned according to ranging sequence feature in order to produce similarly sized bins (number of genes in each bin is reported on top of bars). For panels A-D, data merged using a weighted mean between Foster and colleagues [64] (with genome accession NC_000913.2) and Wei and colleagues [65] (NC_000913.3), considering the total proportion of CDS mutations contributed by each (Foster:Wei 0.65:0.35). The data and code needed to reproduce this Figure can be found in https://doi.org/10.5281/zenodo.23263169.
(PDF)

S6 Fig Average proportion of genes with zero mutations per sequence feature, for Foster data only. Features include gene length (**A**), GC content (**B**), deviation of CpG content from that expected by mononucleotide content (**C**), relative position from origin (**D**), protein abundance from PaxDB [78] (**E**), and median transcript abundance from a compendium of RNA-seq data [79] (**F**), strand orientation relative to the origin (**G**, scored as 0 for lagging strand, 1 for leading strand), gene essentiality classification from the PEC database [77] (**H**, therein scored as 2 for non-essential and 1 for essential). For all panels, genes binned according to ranging sequence feature in order to produce similarly sized bins (number of genes in each bin is reported on top of bars). Mutation data retrieved from Foster and colleagues [64] (with genome accession NC_000913.2). The data and code needed to reproduce this Figure can be found in https://doi.org/10.5281/zenodo.23263169.
(PDF)

S7 Fig Average mutation density per gene length controlling for total gene sequence. To assess mutational density across gene sizes, genes were ranked by length and divided into bins of approximately constant total sequence length such that for a total sequence of *N* bases and *M* bins, each bin contained approximately *N*/*M* bases, resulting in fewer genes in bins with larger gene sizes. Mutational density was calculated for each bin by dividing the total count of observed mutations by the total sequence length within that bin. To control for potential artefacts arising from arbitrary bin selections, the analysis was repeated using multiple combinations of bin sizes and numbers ranging from 4 to 30 bins (**A–I**), total sequence per bin reported in each plot. Spearman correlation between mean gene length and mutation density and respective *P*-value reported in each plot. Mutation data merged from Foster and colleagues [64] (annotated to genome accession NC_000913.2) and Wei and colleagues [65] (NC_000913.3). The data and code needed to reproduce this Figure can be found in https://doi.org/10.5281/zenodo.23263169.
(PDF)

S8 Fig Average mutation density per gene length controlling for total gene sequence, for Foster data only. To assess mutational density across gene sizes, genes were ranked by length and divided into bins of approximately constant total sequence length such that for a total sequence of *N* bases and *M* bins, each bin contained approximately *N*/*M* bases, resulting in fewer genes in bins with larger gene sizes. Mutational density was calculated for each bin by dividing the total count of observed mutations by the total sequence length within that bin. To control for potential artefacts arising from arbitrary bin selections, the analysis was repeated using multiple combinations of bin sizes and numbers ranging from 4 to 30 bins (**A–I**), total sequence per bin reported in each plot. Spearman correlation between mean gene length and mutation density and respective *P*-value reported in each plot. Mutation data retrieved from Foster and colleagues [64] (annotated to genome accession NC_000913.2). The data and code needed to reproduce this Figure can be found in https://doi.org/10.5281/zenodo.23263169.
(PDF)

S9 Fig Mutation density by gene feature for observed and simulated data (full Kendall correlations). Correlations determined between mutation density and relevant gene features. Features include gene length (**A**), GC content (**B**), deviation of CpG content from that expected by mononucleotide content (**C**), relative position from origin (**D**), protein abundance from PaxDB [78] (**E**), and median transcript abundance from a compendium of RNA-seq data [79] (**F**), strand orientation relative to the origin (**G**, scored as 0 for lagging strand, 1 for leading strand), gene essentiality classification from the PEC database [77] (**H**, therein scored as 2 for non-essential and 1 for essential). For all panels, the values found for each of the 10,000 simulated mutation densities are shown in the blue histograms and their mean by the black line, whilst the value from the observed mutation density by the orange line. The *Z* score displayed at the top right of each plot represents the deviation of observed from the simulated null calculated by subtracting the mean simulated value from the observed value and dividing it by the standard deviation of the simulated values. The data and code needed to reproduce this Figure can be found in https://doi.org/10.5281/zenodo.23263169.
(PDF)

S10 Fig Mutation density by gene feature for observed and simulated data (partial Kendall correlations). Correlations determined between mutation density and relevant gene features. Features include gene length (**A**), GC content (**B**), deviation of CpG content from that expected by mononucleotide content (**C**), relative position from origin (**D**), protein abundance from PaxDB [78] (**E**), and median transcript abundance from a compendium of RNA-seq data [79] (**F**), strand orientation relative to the origin (**G**, scored as 0 for lagging strand, 1 for leading strand), gene essentiality classification from the PEC database [77] (**H**, therein scored as 2 for non-essential and 1 for essential). For all panels, the values found for each of the 10,000 simulated mutation densities are shown in the blue histograms and their mean by the black line, whilst the value from the observed mutation density by the orange line. The *Z* score displayed at the top right of each plot represents the deviation of observed from the simulated null calculated by subtracting the mean simulated value from the observed value and dividing it by the standard deviation of the simulated values. The data and code needed to reproduce this Figure can be found in https://doi.org/10.5281/zenodo.23263169.
(PDF)

S11 Fig Mutation density by bivariate gene feature for observed and simulated data. For strand (**A**) and essentiality (**B**), the mean difference in densities between the two groups was found (as indicated on the *x* axis). Strand orientation is relative to the origin (scored as 0 for lagging strand, 1 for leading strand) and gene essentiality classification was taken from the PEC database [77] (therein scored as 2 for non-essential and 1 for essential). For all panels, the values found for each of the 10,000 simulated mutation densities are shown in the blue histograms and their mean by the black line, whilst the value from the observed mutation density by the orange line. The *Z* score displayed at the top right of each plot represents the deviation of observed from the simulated null calculated by subtracting the mean simulated value from the observed value and dividing it by the standard deviation of the simulated values. The data and code needed to reproduce this Figure can be found in https://doi.org/10.5281/zenodo.23263169.
(PDF)

S12 Fig Mutation density by bivariate gene feature for observed and simulated data, controlling for RNA expression. Genes are divided by strand (**A, B**) and essentiality (**C, D**), to find the mean difference in densities between the two groups of the alternative feature (as indicated on the *x* axis). Strand orientation is relative to the origin (scored as 0 for lagging strand, 1 for leading strand) and gene essentiality classification was taken from the PEC database [77] (therein scored as 2 for non-essential and 1 for essential). For all panels, the values found for each of the 10,000 simulated mutation densities are shown in the blue histograms and their mean by the black line, whilst the value from the observed mutation density by the orange line. The simulated data was conditional on the expression level of the gene containing the mutation. The *Z* score displayed at the top right of each plot represents the deviation of observed from the simulated null calculated by subtracting the mean simulated value from the observed value and dividing it by the standard deviation of the simulated values. The data and code needed to reproduce this Figure can be found in https://doi.org/10.5281/zenodo.23263169.
(PDF)

S13 Fig Relationship between average transcriptional propensity, transcript abundance and mutation density. Transcriptional propensity for each locus was calculated using a reporter insertion data set [81] and finding the average RNA/DNA values of any reporter construct inserted within a 2,000 bp window (±1,000 bp) centred on the gene’s midway point. (**A**) The association between average transcriptional propensity of a locus and its median transcript abundance (“RNA”, retrieved from a compendium of RNA-seq data [79]). (**B**) The association between average transcriptional propensity and mutation density. Mutation data retrieved from Wei and colleagues [65] only as it matches the genome version used in the transcriptional propensity data set (NC_000913.3). In both panels, the line represents orthogonal regression between the two variables and the Kendall correlation and the corresponding *P*-value are displayed. (**C**) Mutation density by transcriptional propensity for observed and simulated mutation data. All full Kendall correlation values found for each of the 10,000 simulated mutation densities are shown in the blue histogram and their mean by the black line, whilst the value from the observed mutation density by the orange line. The *Z* score displayed at the top right represents the deviation of observed from the simulated null calculated by subtracting the mean simulated value from the observed value and dividing it by the standard deviation of the simulated values. The *P*-value displayed is inferred from the *Z* score via R’s pnorm function under a two-tailed assumption. The data and code needed to reproduce this Figure can be found in https://doi.org/10.5281/zenodo.23263169.
(PDF)

S14 Fig Mutation accumulation data merged from MMR-deficient strains. Breakdown of Mutation accumulation data combined from MMR strains generated by Foster and colleagues [64] and Wei and colleagues [65]. Mutations are assembled as those occurring in the whole-genome sequence and then filtered to include only those in the coding sequence (CDS). The proportion of CDS mutations contributed by each data set to the full CDS set are Foster:Wei 0.08:0.92. For Foster data, mutations are tracked back to *E. coli* genome NC_000913.2 (with 4321 genes and 4,073,876 total CDS bp), for Wei data NC_000913.3 (with 4308 genes and 4,005,730 total CDS bp). See S14 Table for mutation annotations per gene. The data and code needed to reproduce this Figure can be found in https://doi.org/10.5281/zenodo.23263169.
(PDF)

S15 Fig Average mutation density per gene length controlling for total gene sequence, for MMR-deficient strains. To assess mutational density across gene sizes, genes were ranked by length and divided into bins of approximately constant total sequence length such that for a total sequence of *N* bases and *M* bins, each bin contained approximately *N*/*M* bases, resulting in fewer genes in bins with larger gene sizes. Mutational density was calculated for each bin by dividing the total count of observed mutations by the total sequence length within that bin. To control for potential artefacts arising from arbitrary bin selections, the analysis was repeated using multiple combinations of bin sizes and numbers ranging from 4 to 30 bins (**A–I**), total sequence per bin reported in each plot. Spearman correlation between mean gene length and mutation density and respective *P*-value reported in each plot. Mutation data merged from Foster and colleagues [64] (annotated to genome accession NC_000913.2) and Wei and colleagues [65] (NC_000913.3). The data and code needed to reproduce this Figure can be found in https://doi.org/10.5281/zenodo.23263169.
(PDF)

S16 Fig Relationship between average transcriptional propensity, transcript abundance and mutation density, in MMR-deficient strains. Transcriptional propensity for each locus was calculated using a reporter insertion data set [81] and finding the average RNA/DNA values of any reporter construct inserted within a 2,000 bp window (±1,000 bp) centred on the gene’s midway point. (**A**) The association between average transcriptional propensity of a locus and its median transcript abundance (“RNA”, retrieved from a compendium of RNA-seq data [79]). (**B**) The association between average transcriptional propensity and mutation density. Mutation data retrieved from Wei and colleagues [65] only as it matches the genome version used in the transcriptional propensity data set (NC_000913.3). In both panels, the line represents orthogonal regression between the two variables and the Kendall correlation and the corresponding *P*-value are displayed. (**C**) Mutation density by transcriptional propensity for observed and simulated mutation data. All full Kendall correlation values found for each of the 10,000 simulated mutation densities are shown in the blue histogram and their mean by the black line, whilst the value from the observed mutation density by the orange line. The *Z* score displayed at the top right represents the deviation of observed from the simulated null calculated by subtracting the mean simulated value from the observed value and dividing it by the standard deviation of the simulated values. The *P*-value displayed is inferred from the *Z* score via R’s pnorm function under a two-tailed assumption. The data and code needed to reproduce this Figure can be found in https://doi.org/10.5281/zenodo.23263169.
(PDF)

S17 Fig Trinucleotide mutation frequencies in MMR-deficient strains. Trinucleotides are rank-ordered on the *x* axis form most to least mutating and colour-coded in (**A**) by the mutating (middle) base, and in (**B**) by the GC content of the flanking nucleotides. Mutation data merged from Foster and colleagues [64] (annotated to genome accession NC_000913.2) and Wei and colleagues [65] (NC_000913.3). The data and code needed to reproduce this Figure can be found in https://doi.org/10.5281/zenodo.23263169.
(PDF)

S18 Fig Mutation accumulation data merging and mutation profiles of strictly WT data. (**A**) Breakdown of Mutation accumulation data combined from WT strains generated by Foster and colleagues [64], Long and colleagues [66], Sane and colleagues [67], Tincher and colleagues [68] and Wei and colleagues [65]. Mutations are assembled as those occurring in the whole genome sequence and then filtered to include only those in the coding sequence (CDS). For Foster data, mutations are tracked back to *E. coli* genome NC_000913.2 (with 4321 genes and 4,073,876 total CDS bp), for the rest of the data NC_000913.3 (with 4308 genes and 4,005,730 total CDS bp). (**B–D**) Distribution of trinucleotide mutation differences for each pairwise codon site comparison. Shapiro-Wilk normality test results displayed on the plot, as well as those of a paired Wilcoxon Signed-Rank test. Trinucleotides were formed such that the middle focal base is the mutating one and can occur at within-codon site 1, 2, or 3 (as labelled). Despite non-normality, we include mean and SEM for information. Trinucleotide mutations are determined per occurrence of the trinucleotide in the respective protein-coding genome (and version). (**E**) Ka/Ks estimation for observed and simulated mutation data. (**F**) Number of nonsense mutations for observed and simulated data. For E-F, the values found for each of the 10,000 simulated mutation densities are shown in the blue histograms and their mean by the black line, whilst the value from the observed mutation density by the orange line. The *Z* score displayed at the top right of each plot represents the deviation of observed from the simulated null calculated by subtracting the mean simulated value from the observed value and dividing it by the standard deviation of the simulated values. The *P*-value displayed is inferred from the *Z* score via R’s pnorm function under a two-tailed assumption. The data and code needed to reproduce this Figure can be found in https://doi.org/10.5281/zenodo.23263169.
(PDF)

S19 Fig Mutation density by gene feature for observed and simulated data (partial Kendall correlations), for “purely” WT *E. coli* strains. Correlations determined between mutation density and relevant gene features. Features include gene length (**A**), GC content (**B**), deviation of CpG content from that expected by mononucleotide content (**C**), relative position from origin (**D**), protein abundance from PaxDB [78] (**E**), and median transcript abundance from a compendium of RNA-seq data [79] (**F**), strand orientation relative to the origin (**G**, scored as 0 for lagging strand, 1 for leading strand), gene essentiality classification from the PEC database [77] (**H**, therein scored as 2 for non-essential and 1 for essential). For all panels, the values found for each of the 10,000 simulated mutation densities are shown in the blue histograms and their mean by the black line, whilst the value from the observed mutation density by the orange line. The *Z* score displayed at the top right of each plot represents the deviation of observed from the simulated null calculated by subtracting the mean simulated value from the observed value and dividing it by the standard deviation of the simulated values. Mutation data includes sample PFM2 normal from Foster and colleagues [64], Wei data as described in Methods [65], data from Sane and colleagues [67], Tincher and colleagues [68]and Long and colleagues [66] for WT samples. The data and code needed to reproduce this Figure can be found in https://doi.org/10.5281/zenodo.23263169.
(PDF)

S20 Fig Mutation density by gene feature for observed and simulated data (partial Kendall correlations), for *S. pneumoniae.* Correlations determined between mutation density and relevant gene features. Features include gene length (**A**), GC content (**B**), deviation of CpG content from that expected by mononucleotide content (**C**), relative position from origin (**D**), strand orientation relative to the origin (**E**, scored as 0 for lagging strand, 1 for leading strand), and transcript abundance [111] (**F**). For all panels, the values found for each of the 10,000 simulated mutation densities are shown in the blue histograms and their mean by the black line, whilst the value from the observed mutation density by the orange line. The *Z* score displayed at the top right of each plot represents the deviation of observed from the simulated null calculated by subtracting the mean simulated value from the observed value and dividing it by the standard deviation of the simulated values. The data and code needed to reproduce this Figure can be found in https://doi.org/10.5281/zenodo.23263169.
(PDF)

S21 Fig Mutation density by gene feature for observed and simulated data (partial Kendall correlations), for *M. smegmatis.* Correlations determined between mutation density and relevant gene features. Features include gene length (**A**), GC content (**B**), deviation of CpG content from that expected by mononucleotide content (**C**), relative position from origin (**D**), strand orientation relative to the origin (**E**, scored as 0 for lagging strand, 1 for leading strand), and transcript abundance averaged across the growth phase [114] (**F**). For all panels, the values found for each of the 10,000 simulated mutation densities are shown in the blue histograms and their mean by the black line, whilst the value from the observed mutation density by the orange line. The *Z* score displayed at the top right of each plot represents the deviation of observed from the simulated null calculated by subtracting the mean simulated value from the observed value and dividing it by the standard deviation of the simulated values. The data and code needed to reproduce this Figure can be found in https://doi.org/10.5281/zenodo.23263169.
(PDF)

S22 Fig Mutation density by strand orientation for observed and simulated data. For all panels, strand orientation is relative to the origin of replication (scored as 0 for lagging (−) strand, 1 for leading (+) strand). Observed mutation density determined as mutation count per gene normalised by gene length. (**A**) Mutation data for *S. pneumoniae* retrieved for WT samples from Jiang and colleagues [111]. (**B**) Mutation data for *M. smegmatis* combined from WT samples from Kucukyildirim and colleagues [88] and Castañeda-García and colleagues [89]. For A-B, error bars represent standard error of the mean (SEM), a Mann–Whitney *U* test was performed and the *U* statistic with respective *P*-value displayed in each plot. For simulated mutation data for (**C**) *S. pneumoniae* and (**D**) *M. smegmatis*, the mean difference in densities between the two groups was found (as indicated on the *x* axis). The values found for each of the 10,000 simulated mutation densities are shown in the blue histograms and their mean by the black line, whilst the value from the observed mutation density by the orange line. The *Z* score displayed at the top right of each plot represents the deviation of observed from the simulated null calculated by subtracting the mean simulated value from the observed value and dividing it by the standard deviation of the simulated values. The data and code needed to reproduce this Figure can be found in https://doi.org/10.5281/zenodo.23263169.
(PDF)

S23 Fig Testing for lack of selection in mutation data for *S. cerevisiae.* (**A**) Ka/Ks estimation for observed and simulated mutation data. (**B**) Number of nonsense mutations for observed and simulated data. Mutation data merged from four MA experiments [90–93]. The values found for each of the 10,000 simulated mutation densities are shown in the blue histograms and their mean by the black line, whilst the value from the observed mutation density by the orange line. The *Z* score displayed at the top right of each plot represents the deviation of observed from the simulated null calculated by subtracting the mean simulated value from the observed value and dividing it by the standard deviation of the simulated values. The *P*-value displayed is inferred from the *Z* score via R’s pnorm function under a two-tailed assumption. The data and code needed to reproduce this Figure can be found in https://doi.org/10.5281/zenodo.23263169.
(PDF)

S24 Fig Mutation density by gene feature for observed and simulated data (full Kendall correlations), for *S. cerevisiae.* Correlations determined between mutation density and relevant gene features. Features include gene length (**A**), GC content (**B**), deviation of CpG content from that expected by mononucleotide content (**C**), transcript abundance from the NCBI GEO Database [94] (**D**), protein abundance from PaxDB [78] (**E**), gene essentiality classification from the Yeast Phenome Database [95] (**F**, therein scored as 2 for non-essential and 1 for essential), and gene replication timing obtained from Berners-Lee [96] (**G**). For all panels, the values found for each of the 10,000 simulated mutation densities are shown in the blue histograms and their mean by the black line, whilst the value from the observed mutation density by the orange line. The *Z* score displayed at the top right of each plot represents the deviation of observed from the simulated null calculated by subtracting the mean simulated value from the observed value and dividing it by the standard deviation of the simulated values. Mutation data merged from four MA experiments [90–93]. The data and code needed to reproduce this Figure can be found in https://doi.org/10.5281/zenodo.23263169.
(PDF)

S25 Fig Mutation density by gene feature for observed and simulated data (partial Kendall correlations), for *S. cerevisiae.* Correlations determined between mutation density and relevant gene features. Features include gene length (**A**), GC content (**B**), deviation of CpG content from that expected by mononucleotide content (**C**), transcript abundance from the NCBI GEO Database [94] (**D**), protein abundance from PaxDB [78] (**E**), gene essentiality classification from the Yeast Phenome Database [95] (**F**, therein scored as 2 for non-essential and 1 for essential), and gene replication timing obtained from Berners-Lee [96] (**G**). For all panels, the values found for each of the 10,000 simulated mutation densities are shown in the blue histograms and their mean by the black line, whilst the value from the observed mutation density by the orange line. The *Z* score displayed at the top right of each plot represents the deviation of observed from the simulated null calculated by subtracting the mean simulated value from the observed value and dividing it by the standard deviation of the simulated values. Mutation data merged from four MA experiments [90–93]. The data and code needed to reproduce this Figure can be found in https://doi.org/10.5281/zenodo.23263169.
(PDF)

S26 Fig Mutation density by gene essentiality for observed and simulated data, for *S. cerevisiae.* Gene essentiality classification was taken from the Yeast Phenome Database [95] (therein scored as 2 for non-essential and 1 for essential). (**A**) Simulated data. The mean difference in densities between the two groups was found (as indicated on the *x* axis). The values found for each of the 10,000 simulated mutation densities are shown in the blue histograms and their mean by the black line, whilst the value from the observed mutation density by the orange line. The *Z* score displayed at the top right of each plot represents the deviation of observed from the simulated null calculated by subtracting the mean simulated value from the observed value and dividing it by the standard deviation of the simulated values. (**B**) Observed average mutation density across the two groups. Error bars represent standard error of the mean (SEM). A Mann–Whitney *U* test was performed and the *U* statistic with respective *P*-value displayed in the plot. The data and code needed to reproduce this Figure can be found in https://doi.org/10.5281/zenodo.23263169.
(PDF)

S27 Fig Testing for lack of selection in mutation data from MMR-deficient strains. Ka/Ks estimation for observed and simulated mutation data. For (**A**), mutation data merged from Foster and colleagues [64] (with *E. coli* genome NC_000913.2) and Wei and colleagues [65] (NC_000913.3). (**B**) include Foster data only, **C** Wei data only. For all panels, the values found for each of the 10,000 simulated mutation densities are shown in the blue histograms and their mean by the black line, whilst the value from the observed mutation density by the orange line. The *Z* score displayed at the top right of each plot represents the deviation of observed from the simulated null calculated by subtracting the mean simulated value from the observed value and dividing it by the standard deviation of the simulated values. The *P*-value displayed is inferred from the *Z* score via R’s pnorm function under a two-tailed assumption. The data and code needed to reproduce this Figure can be found in https://doi.org/10.5281/zenodo.23263169.
(PDF)

S1 Table Comprehensive list of all *E. coli* annotated genomic sequences with respective annotated features and mutation counts. Mutation data combined from Wei and colleagues [65] and Foster and colleagues [64]. All annotations are based on the *E. coli* K-12 MG1655 reference genome (for Foster data version NC_000913.2 and for Wei data NC_000913.3, both available on NCBI [108]). For each, table includes gene identifiers (locus tag, gene name), sequence type (CDS, ncRNA, rRNA, or tRNA), classification as protein-coding or non-coding, genomic position (start:end), strand orientation with respect to the genome (“gff_strand”) or relative to the origin of replication (“rel_strand”) (“+” as leading strand, “-” as lagging strand), gene length (in base pairs, bp), proportion of sequence within the 5′ end (first 90 bp), relative distance from the origin of replication (“rel_pos”), GC content, CpG content, deviation of CpG from expected based on mononucleotide frequencies, essentiality score (following PEC database classification [77]: essential = 1, non-essential = 2, unknown = 3), protein abundance (values from PaxDB [78]), and transcript abundance (values from a compendium of RNA-seq data [79], handled in four variations: mean or median, treating no data as zero or NA). Where relevant, both the values according to the reference genome used by Foster and Wei are included (as indicated in the column name). For GC content, CpG deviation and relative position from the origin, weighted means were used taking into account the proportion of CDS mutations contributed by each data set to the merged CDS mutation data (Foster: 0.65, Wei: 0.35). For these values, columns containing “_WeightedMeanNormCDS” contain the mean weighted value if a locus tag is present in both genomes, if not it keeps the value of the genes in which it is present. Values in columns containing “_WeightedMeanNormAll” follow a similar logic, though weighing the values based on the proportion of each data set in the total mutation set, i.e., not CDS mutations only (Foster: 0.61, Wei: 0.39). Mutation counts (“mut_cnt”) are adjusted for overlapping genes, such that a mutation occurring in two overlapping genes contributes 0.5 to each. These are included for both data sets separately and summed across Foster and Wei data (“mut_cnt_sum”). Mutation density (“mut_density”) determined as mutation count per gene divided by normalised weighted mean of gene length. The data and code needed to reproduce this Table can be found in https://doi.org/10.5281/zenodo.23263169.
(CSV)

S2 Table Full (top) and partial (bottom) Kendall correlation analysis between mutation density and all annotated gene features, for Foster data only. Mutation density (“mut_density”) determined as mutation count per gene normalised by gene length. Features include gene length (“gene_len”), GC content (“GC”), deviation of CpG content from that expected by mononucleotide content (“CpG_dev”), relative position from origin (“rel_pos”), gene essentiality classification from the PEC database [77] (“nonessentiality”, scored as 2 for non-essential and 1 for essential), strand orientation relative to the origin (“strand”, scored as 0 for lagging strand, 1 for leading strand), protein abundance from PaxDB [78] (“prot_abundance”), and median transcript abundance from a compendium of RNA-seq data [79] (“RNA_abundance”). Mutation data retrieved from Foster and colleagues [64] for a total of 1,676 CDS mutations annotated to the reference *E. coli* genome NC_000913.2. Stars represent *P*-value (*P* < 0.0001 **** *P* < 0.001 *** *P* < 0.01 ** *P* < 0.05 * *P* > 0.05 no stars). The data and code needed to reproduce this Table can be found in https://doi.org/10.5281/zenodo.23263169.
(CSV)

S3 Table Reference study information for each entry in compendium of RNAseq transcript abundance data [79]. The data and code needed to reproduce this Table can be found in https://doi.org/10.5281/zenodo.23263169.
(CSV)

S4 Table Full (top) and partial (bottom) Kendall correlation analysis between mutation density and all annotated gene features, for expression data under LB conditions. Mutation density (“mut_density”) determined as mutation count per gene normalised by gene length. Features in panel **A** include gene length (“gene_len”), GC content (“GC”), deviation of CpG content from that expected by mononucleotide content (“CpG_dev”), relative position from origin (“rel_pos”), gene essentiality classification from the PEC database [77] (“nonessentiality”, scored as 2 for non-essential and 1 for essential), strand orientation relative to the origin (“strand”, scored as 0 for lagging strand, 1 for leading strand), protein abundance from PaxDB [78] restricted to only experiments conducted in LB medium (“LB_protein”), and transcript abundance from an experiment conducted in LB medium by Martincorena and colleagues [1] (“LB_transcript”). Panel **B** includes the same features as panel A, except for gene length. In both, mutation data combined from Foster and colleagues [64] and Wei and colleagues [65] for a total of 2,577 CDS mutations annotated respectively to the reference *E. coli* genomes NC_000913.2 and NC_000913.3. For GC content, CpG deviation, gene length and relative position from the origin, weighted means were used taking into account the proportion of CDS mutations contributed by each data set to the merged CDS mutation data (Foster: 0.65, Wei: 0.35). Stars represent *P*-value (*P* < 0.0001 **** *P* < 0.001 *** *P* < 0.01 ** *P* < 0.05 * *P* > 0.05 no stars). The data and code needed to reproduce these Tables can be found in https://doi.org/10.5281/zenodo.23263169.
(CSV)

S5 Table Full (top) and partial (bottom) Kendall correlation analysis between mutation density and all annotated gene features. Mutation density (“mut_density”) determined as mutation count per gene normalised by gene length. Features include gene length (“gene_len”), GC content (“GC”), deviation of CpG content from that expected by mononucleotide content (“CpG_dev”), relative position from the closest midway point between the origin and terminus (such that the closer the gene is to it, the lower the value, “rel_pos_fluct”), gene essentiality classification from the PEC database [77] (“nonessentiality”, scored as 2 for non-essential and 1 for essential), strand orientation relative to the origin (“strand”, scored as 0 for lagging strand, 1 for leading strand), protein abundance from PaxDB [78] (“prot_abundance”), and median transcript abundance from a compendium of RNA-seq data [79] (“RNA_abundance”). Mutation data combined from Foster and colleagues [64] and Wei and colleagues [65] for a total of 2,577 CDS mutations annotated respectively to the reference *E. coli* genomes NC_000913.2 and NC_000913.3. For GC content, CpG deviation, gene length and relative position from the origin, weighted means were used taking into account the proportion of CDS mutations contributed by each data set to the merged CDS mutation data (Foster: 0.65, Wei: 0.35). Stars represent *P*-value (*P* < 0.0001 **** *P* < 0.001 *** *P* < 0.01 ** *P* < 0.05 * *P* > 0.05 no stars). The data and code needed to reproduce this Table can be found in https://doi.org/10.5281/zenodo.23263169.
(CSV)

S6 Table Kendall correlation and partial correlation analysis between mutation density and all annotated gene features, for observed and simulated data, using Foster data only. “observed_cor” refers to the full Kendall’s tau between observed mutation density and each feature, whilst “observed_pcor” refers to the Kendall partial tau. The *P*-values for both are represented with stars such that *P* < 0.0001 **** *P* < 0.001 *** *P* < 0.01 ** *P* < 0.05 * *P* > 0.05 no stars. “sim_mean_cor” and “sim_mean_pcor” respectively represent the mean of full and partial Kendall’s tau values across 10,000 simulations of mutational density. For each, standard deviations are also included. “Zscore_cor” and “Zscore_pcor” represent the deviation in observed tau to the simulated null calculated by subtracting the mean simulated tau from the observed tau and dividing it by the standard deviation of the simulated tau values (respectively for full and partial tau values). Considered features include gene length, GC content, deviation of CpG content from that expected by mononucleotide content (“CpG deviation”), relative position from origin of replication (“relative position”), gene essentiality classification from the PEC database [77] (“nonessentiality”, therein scored as 2 for non-essential and 1 for essential), strand orientation relative to the origin (“strand”, scored as 0 for lagging strand, 1 for leading strand), protein abundance from PaxDB [78] (“protein”), and median transcript abundance from a compendium of RNA-seq data [79] (“RNA”). Mutation data was retrieved from Foster and colleagues [64] (annotated to genome accession NC_000913.2). For simulated data *P*-value is inferred from the *Z* score via R’s pnorm function under two-tailed assumptions and also included in the table such that *P* < 0.0001 **** *P* < 0.001 *** *P* < 0.01 ** *P* < 0.05 * *P* > 0.05 no stars. The data and code needed to reproduce this Table can be found in https://doi.org/10.5281/zenodo.23263169.
(CSV)

S7 Table Kendall correlation and partial correlation analysis between mutation density and all annotated gene features, for observed and simulated data, using expression data under LB conditions. “observed_cor” refers to the full Kendall’s tau between observed mutation density and each feature, whilst “observed_pcor” refers to the Kendall partial tau. The *P*-values for both are represented with stars such that *P* < 0.0001 **** *P* < 0.001 *** *P* < 0.01 ** *P* < 0.05 * *P* > 0.05 no stars. “sim_mean_cor” and “sim_mean_pcor” respectively represent the mean of full and partial Kendall’s tau values across 10,000 simulations of mutational density. For each, standard deviations are also included. “Zscore_cor” and “Zscore_pcor” represent the deviation in observed tau to the simulated null calculated by subtracting the mean simulated tau from the observed tau and dividing it by the standard deviation of the simulated tau values (respectively for full and partial tau values). Considered features include gene length, GC content, deviation of CpG content from that expected by mononucleotide content (“CpG deviation”), relative position from origin of replication (“relative position”), gene essentiality classification from the PEC database [77] (“nonessentiality”, scored as 2 for non-essential and 1 for essential), strand orientation relative to the origin (“strand”, scored as 0 for lagging strand, 1 for leading strand), protein abundance from PaxDB [78] restricted to only experiments conducted in LB medium (“protein”), and transcript abundance from an experiment conducted in LB medium by Martincorena and colleagues [1] (“RNA”). Mutation data was merged from Foster and colleagues [64] (annotated to genome accession NC_000913.2) and Wei and colleagues [65] (NC_000913.3). For GC content, CpG deviation and relative position from the origin, weighted means across the two genomes were used taking into account the proportion of CDS mutations contributed by each data set to the merged CDS mutation data (Foster: 0.65, Wei: 0.35). For simulated data *P*-value is inferred from the *Z* score via R’s pnorm function under two-tailed assumptions and also included in the table such that *P* < 0.0001 **** *P* < 0.001 *** *P* < 0.01 ** *P* < 0.05 * *P* > 0.05 no stars. The data and code needed to reproduce this Table can be found in https://doi.org/10.5281/zenodo.23263169.
(CSV)

S8 Table Spearman correlation and partial correlation analysis between mutation density and all annotated gene features, for observed and simulated data. “observed_cor” refers to the full Kendall’s tau between observed mutation density and each feature, whilst “observed_pcor” refers to the Kendall partial tau. The *P*-values for both are represented with stars such that *P* < 0.0001 **** *P* < 0.001 *** *P* < 0.01 ** *P* < 0.05 * *P* > 0.05 no stars. “sim_mean_cor” and “sim_mean_pcor” respectively represent the mean of full and partial Kendall’s tau values across 10,000 simulations of mutational density. For each, standard deviations are also included. “Zscore_cor” and “Zscore_pcor” represent the deviation in observed tau to the simulated null calculated by subtracting the mean simulated tau from the observed tau and dividing it by the standard deviation of the simulated tau values (respectively for full and partial tau values). Considered features include gene length, GC content, deviation of CpG content from that expected by mononucleotide content (“CpG deviation”), relative position from origin of replication (“relative position”), gene essentiality classification from the PEC database [77] (“nonessentiality”, scored as 2 for non-essential and 1 for essential), strand orientation relative to the origin (“strand”, scored as 0 for lagging strand, 1 for leading strand), protein abundance from PaxDB [78] (“protein”), and median transcript abundance from a compendium of RNA-seq data [79] (“RNA”). Mutation data was merged from Foster and colleagues [64] (annotated to genome accession NC_000913.2) and Wei and colleagues [65] (NC_000913.3). For GC content, CpG deviation and relative position from the origin, weighted means across the two genomes were used taking into account the proportion of CDS mutations contributed by each data set to the merged CDS mutation data (Foster: 0.65, Wei: 0.35). For simulated data *P*-value is inferred from the *Z* score via R’s pnorm function under two-tailed assumptions and also included in the table such that *P* < 0.0001 **** *P* < 0.001 *** P < 0.01 ** P < 0.05 * *P* > 0.05 no stars. The data and code needed to reproduce this Table can be found in https://doi.org/10.5281/zenodo.23263169.
(CSV)

S9 Table Analysis between mutation density and bivariate gene features, for observed and simulated data. Features include gene essentiality classification from the PEC database [77] (“nonessentiality”, therein scored as 2 for non-essential and 1 for essential) and strand orientation relative to the origin of replication (“strand”, scored as 0 for lagging strand, 1 for leading strand). “observed_diff” represents the difference in mutation density between the two groups (leading – lagging or essential – nonessential) using the observed mutation counts. “sim_mean_diff”, “sim_median_diff” and “sim_sd_diff” respectively represent the mean, median and standard deviation of mutation counts across 10,000 simulations. “Zscore_dev” represents the deviation in observed value to the simulated null calculated by subtracting the mean simulated value from the observed value and dividing it by the standard deviation of the simulated values. Mutation data merged from Foster and colleagues [64] (annotated to genome accession NC_000913.2) and Wei and colleagues [65] (NC_000913.3). The data and code needed to reproduce this Table can be found in https://doi.org/10.5281/zenodo.23263169.
(CSV)

S10 Table Analysis between mutation density and bivariate gene features, for observed and simulated data, using Foster data only. Features include gene essentiality classification from the PEC database [77] (“nonessentiality”, therein scored as 2 for non-essential and 1 for essential) and strand orientation relative to the origin of replication (“strand”, scored as 0 for lagging strand, 1 for leading strand). “observed_diff” represents the difference in mutation density between the two groups (leading – lagging or essential – nonessential) using the observed mutation counts. “sim_mean_diff”, “sim_median_diff” and “sim_sd_diff” respectively represent the mean, median and standard deviation of mutation counts across 10,000 simulations. “Zscore_dev” represents the deviation in observed value to the simulated null calculated by subtracting the mean simulated value from the observed value and dividing it by the standard deviation of the simulated values. Mutation data retrieved from Foster and colleagues [64] (annotated to genome accession NC_000913.2). The data and code needed to reproduce this Table can be found in https://doi.org/10.5281/zenodo.23263169.
(CSV)

S11 Table Analysis between mutation density and bivariate gene features, for observed and simulated data. Genes are divided by strand and essentiality, to find the mean difference in densities between the two groups of the alternative feature (as indicated in “feature”). Gene essentiality classification was retrieved from the PEC database [77] (“nonessentiality”, scored as 2 for non-essential and 1 for essential) and strand orientation is relative to the origin of replication (“strand”, scored as 0 for lagging strand, 1 for leading strand). “observed_diff” represents the difference in mutation density between the two groups (leading – lagging or essential – nonessential) using the observed mutation counts. “sim_mean_diff” and “sim_sd_diff” respectively represent the mean and standard deviation of mutation counts across 10,000 simulations. The simulated data was conditional on the expression level of the gene containing the mutation. “Zscore_dev” represents the deviation in observed value to the simulated null calculated by subtracting the mean simulated value from the observed value and dividing it by the standard deviation of the simulated values. Mutation data merged from Foster and colleagues [64] (annotated to genome accession NC_000913.2) and Wei and colleagues [65] (NC_000913.3). The data and code needed to reproduce this Table can be found in https://doi.org/10.5281/zenodo.23263169.
(CSV)

S12 Table Analysis between mutation density and bivariate gene features, for observed and simulated data, using Foster data only. Genes are divided by strand and essentiality, to find the mean difference in densities between the two groups of the alternative feature (as indicated in “feature”). Gene essentiality classification was retrieved from the PEC database [77] (“nonessentiality”, scored as 2 for non-essential and 1 for essential) and strand orientation is relative to the origin of replication (“strand”, scored as 0 for lagging strand, 1 for leading strand). “observed_diff” represents the difference in mutation density between the two groups (leading – lagging or essential – nonessential) using the observed mutation counts. “sim_mean_diff” and “sim_sd_diff” respectively represent the mean and standard deviation of mutation counts across 10,000 simulations. The simulated data was conditional on the expression level of the gene containing the mutation. “Zscore_dev” represents the deviation in observed value to the simulated null calculated by subtracting the mean simulated value from the observed value and dividing it by the standard deviation of the simulated values. Mutation data retrieved from Foster and colleagues [64] (annotated to genome accession NC_000913.2). The data and code needed to reproduce this Table can be found in https://doi.org/10.5281/zenodo.23263169.
(CSV)

S13 Table Kendall correlation and partial correlation analysis between mutation density and protein abundance, for observed and two sets of simulated data. Protein abundance was retrieved from PaxDB [78]. The two sets of simulated data refer to those generated by randomly reallocating with replacement the total number of observed mutations across the entire set of possible CDS bases in each genome, assuming an equal probability of mutation per base (“gene length simulations”), and a conditional probability based on RNA expression levels of the gene at the CDS position (“gene length and RNA simulations”). RNA expression levels for the second simulated set were considered as median transcript abundances from a compendium of RNA-seq data [79]. “observed_cor” refers to the full Kendall’s tau between observed mutation density and protein abundance, whilst “observed_pcor” refers to the Kendall partial tau. The partial correlation analysis for “gene length simulations” was performed controlling for the following features: gene length, GC content, deviation of CpG content from that expected by mononucleotide content, relative position from origin of replication, gene essentiality classification from the PEC database [77] (scored as 2 for non-essential and 1 for essential), strand orientation relative to the origin (scored as 0 for lagging strand, 1 for leading strand), and median transcript abundance from a compendium of RNA-seq data [79]. The partial correlation analysis for “gene length and RNA simulations” was performed controlling for all the same features, except for RNA expression. The *P*-values for both full and partial tau values are included in brackets. “sim_mean_cor” and “sim_mean_pcor” respectively represent the mean of full and partial Kendall’s tau values across 10,000 simulations of mutational density. For each, standard deviations are also included. “Zscore_cor” and “Zscore_pcor” represent the deviation in observed tau to the simulated null calculated by subtracting the mean simulated tau from the observed tau and dividing it by the standard deviation of the simulated tau values (respectively for full and partial tau). Mutation data was merged from Foster and colleagues [64] (annotated to genome accession NC_000913.2) and Wei and colleagues [65] (NC_000913.3). For GC content, CpG deviation and relative position from the origin, weighted means across the two genomes were used taking into account the proportion of CDS mutations contributed by each data set to the merged CDS mutation data (Foster: 0.65, Wei: 0.35). For simulated data, *P*-value is inferred from the *Z* score via R’s pnorm function under two-tailed assumptions and included in brackets. The data and code needed to reproduce this Table can be found in https://doi.org/10.5281/zenodo.23263169.
(CSV)

S14 Table Comprehensive list of all *E. coli* annotated genomic sequences with respective annotated features and mutation counts, from MMR-deficient strains. Mutation data combined from Wei and colleagues [65] and Foster and colleagues [64]. All annotations are based on the *E. coli* K-12 MG1655 reference genome (for Foster data version NC_000913.2 and for Wei data NC_000913.3, both available on NCBI [108]). For each, table includes gene identifiers (locus tag, gene name), sequence type (CDS, ncRNA, rRNA, or tRNA), classification as protein-coding or non-coding, genomic position (start:end), strand orientation with respect to the genome (“gff_strand”) or relative to the origin of replication (“rel_strand”) (“+” as leading strand, “-” as lagging strand), gene length (in base pairs, bp), proportion of sequence within the 5′ end (first 90 bp), relative distance from the origin of replication (“rel_pos”), GC content, CpG content, deviation of CpG from expected based on mononucleotide frequencies, essentiality score (following PEC database classification [77]: essential = 1, non-essential = 2, unknown = 3), protein abundance (values from PaxDB [78]), and transcript abundance (values from a compendium of RNA-seq data [79], handled in four variations: mean or median, treating no data as zero or NA). Where relevant, both the values according to the reference genome used by Foster and Wei are included (as indicated in the column name). For GC content, CpG deviation and relative position from the origin, weighted means were used taking into account the proportion of CDS mutations contributed by each data set to the merged CDS mutation data (Foster: 0.0805, Wei: 0.9195). For these values, columns containing “_WeightedMeanNormCDS” contain the mean weighted value if a locus tag is present in both genomes, if not it keeps the value of the genes in which it is present. Values in columns containing “_WeightedMeanNormAll” follow a similar logic, though weighing the values based on the proportion of each data set in the total mutation set, i.e., not CDS mutations only (Foster: 0.0813, Wei: 0.9187). Mutation counts (“mut_cnt”) are adjusted for overlapping genes, such that a mutation occurring in two overlapping genes contributes 0.5 to each. These are included for both data sets separately and summed across Foster and Wei data (“mut_cnt_sum”). Mutation density (“mut_density”) determined as mutation count per gene divided by normalised weighted mean of gene length. The data and code needed to reproduce this Table can be found in https://doi.org/10.5281/zenodo.23263169.
(CSV)

S15 Table Full (top) and partial (bottom) Kendall correlation analysis between annotated gene features. Features include gene length (“gene_len”), GC content (“GC”), deviation of CpG content from that expected by mononucleotide content (“CpG_dev”), relative position from origin (“rel_pos”), gene essentiality classification from the PEC database [77] (“nonessentiality”, scored as 2 for non-essential and 1 for essential), strand orientation relative to the origin (“strand”, scored as 0 for lagging strand, 1 for leading strand), protein abundance from PaxDB [78] (“prot_abundance”), and median transcript abundance from a compendium of RNA-seq data [79] (“RNA”). For GC content, CpG deviation, gene length and relative position from the origin, weighted means across *E. coli* genomes NC_000913.2 and NC_000913.3 were used taking into account the proportion of CDS mutations contributed by each data set to the merged CDS mutation data (NC_000913.2: 0.65, NC_000913.3: 0.35). Stars represent *P*-value (*P* < 0.0001 **** *P* < 0.001 *** *P* < 0.01 ** *P* < 0.05 * *P* > 0.05 no stars). The data and code needed to reproduce this Table can be found in https://doi.org/10.5281/zenodo.23263169.
(CSV)

S16 Table Full (top) and partial (bottom) Kendall correlation analysis between mutation density and all annotated gene features, for MMR-deficient strains. Mutation density (“mut_density”) determined as mutation count per gene normalised by gene length. Features include gene length (“gene_len”), GC content (“GC”), deviation of CpG content from that expected by mononucleotide content (“CpG_dev”), relative position from the closest midway point between the origin and terminus (such that the closer the gene is to it, the lower the value, “rel_pos_fluct”), gene essentiality classification from the PEC database [77] (“nonessentiality”, scored as 2 for non-essential and 1 for essential), strand orientation relative to the origin (“strand”, scored as 0 for lagging strand, 1 for leading strand), protein abundance from PaxDB [78] (“prot_abundance”), and median transcript abundance from a compendium of RNA-seq data [79] (“RNA”). Mutation data combined from Foster and colleagues [64] and Wei and colleagues [65] for a total of 74,007 CDS mutations annotated respectively to the reference *E. coli* genomes NC_000913.2 and NC_000913.3. For GC content, CpG deviation, gene length and relative position from the origin, weighted means were used taking into account the proportion of CDS mutations contributed by each data set to the merged CDS mutation data (Foster: 0.08, Wei: 0.92). Stars represent *P*-value (*P* < 0.0001 **** *P* < 0.001 *** *P* < 0.01 ** *P* < 0.05 * *P* > 0.05 no stars). The data and code needed to reproduce this Table can be found in https://doi.org/10.5281/zenodo.23263169.
(CSV)

S17 Table Full (top) and partial (bottom) Kendall correlation analysis between mutation density and all annotated gene features, for “purely” WT *E. coli* strains. Mutation density (“mut_density”) determined as mutation count per gene normalised by gene length. Features include gene length (“gene_len”), GC content (“GC”), deviation of CpG content from that expected by mononucleotide content (“CpG_dev”), relative position from origin (“rel_pos”), gene essentiality classification from the PEC database [77] (“nonessentiality”, scored as 2 for non-essential and 1 for essential), strand orientation relative to the origin (“strand”, scored as 0 for lagging strand, 1 for leading strand), protein abundance from PaxDB [78] (“prot_abundance”), and median transcript abundance from a compendium of RNA-seq data [79] (“RNA”). Mutation data includes sample PFM2 normal from Foster and colleagues [64], Wei data as described in Methods [65], data from Sane and colleagues [67], Long and colleagues [66] and Tincher and colleagues [68] for WT samples. For GC content, CpG deviation, gene length and relative position from the origin, weighted means were used taking into account the proportion of CDS mutations contributed by each data set to the merged CDS mutation data. Stars represent *P*-value (*P* < 0.0001 **** *P* < 0.001 *** *P* < 0.01 ** *P* < 0.05 * *P* > 0.05 no stars). The data and code needed to reproduce this Table can be found in https://doi.org/10.5281/zenodo.23263169.
(CSV)

S18 Table Kendall correlation and partial correlation analysis between mutation density and all annotated gene features, for observed and simulated data, for “purely” WT *E. coli* strains and using expression data under LB conditions. “observed_cor” refers to the full Kendall’s tau between observed mutation density and each feature, whilst “observed_pcor” refers to the Kendall partial tau. The *P*-values for both are represented with stars such that *P* < 0.0001 **** *P* < 0.001 *** *P* < 0.01 ** *P* < 0.05 * *P* > 0.05 no stars. “sim_mean_cor” and “sim_mean_pcor” respectively represent the mean of full and partial Kendall’s tau values across 10,000 simulations of mutational density. For each, standard deviations are also included. “Zscore_cor” and “Zscore_pcor” represent the deviation in observed tau to the simulated null calculated by subtracting the mean simulated tau from the observed tau and dividing it by the standard deviation of the simulated tau values (respectively for full and partial tau values). Considered features include gene length, GC content, deviation of CpG content from that expected by mononucleotide content (“CpG deviation”), relative position from origin of replication (“relative position”), gene essentiality classification from the PEC database [77] (“nonessentiality”, scored as 2 for non-essential and 1 for essential), strand orientation relative to the origin (“strand”, scored as 0 for lagging strand, 1 for leading strand), protein abundance from PaxDB [78] restricted to only experiments conducted in LB medium (“protein”), and transcript abundance from an experiment conducted in LB medium by Martincorena and colleagues [1] (“RNA”). Mutation accumulation data combined from WT strains generated by Foster and colleagues [64], Long and colleagues [66], Sane and colleagues [67], Tincher and colleagues [68] and Wei and colleagues [65]; Foster data annotated to genome accession NC_000913.2 and the rest to genome accession NC_000913.3. For GC content, CpG deviation and relative position from the origin, weighted means across the two genomes were used taking into account the proportion of CDS mutations contributed by each data set to the merged CDS mutation data. For simulated data *P*-value is inferred from the *Z* score via R’s pnorm function under two-tailed assumptions and also included in the table such that *P* < 0.0001 **** *P* < 0.001 *** *P* < 0.01 ** *P* < 0.05 * *P* > 0.05 no stars. The data and code needed to reproduce this Table can be found in https://doi.org/10.5281/zenodo.23263169.
(CSV)

S19 Table Full (top) and partial (bottom) Kendall correlation analysis between mutation density and all annotated gene features, for MMR-deficient strains from Foster data only. Mutation density (“mut_density”) determined as mutation count per gene normalised by gene length. Features include gene length (“gene_len”), GC content (“GC”), deviation of CpG content from that expected by mononucleotide content (“CpG_dev”), relative position from origin (“rel_pos”), gene essentiality classification from the PEC database [77] (“nonessentiality”, scored as 2 for non-essential and 1 for essential), strand orientation relative to the origin (“strand”, scored as 0 for lagging strand, 1 for leading strand), protein abundance from PaxDB [78] (“prot_abundance”), and median transcript abundance from a compendium of RNA-seq data [79] (“RNA”). Mutation data retrieved from Foster and colleagues [64] for reference *E. coli* genome NC_000913.2. Stars represent *P*-value (*P* < 0.0001 **** *P* < 0.001 *** *P* < 0.01 ** *P* < 0.05 * *P* > 0.05 no stars). The data and code needed to reproduce this Table can be found in https://doi.org/10.5281/zenodo.23263169.
(CSV)

S20 Table Full (top) and partial (bottom) Kendall correlation analysis between mutation density and all annotated gene features. Mutation density (“mut_density”) determined as mutation count per gene normalised by gene length. Features include GC content (“GC”), deviation of CpG content from that expected by mononucleotide content (“CpG_dev”), relative position from origin (“rel_pos”), gene essentiality classification from the PEC database [77] (“nonessentiality”, scored as 2 for non-essential and 1 for essential), strand orientation relative to the origin (“strand”, scored as 0 for lagging strand, 1 for leading strand), protein abundance from PaxDB [78] (“prot_abundance”), and median transcript abundance from a compendium of RNA-seq data [79] (“RNA”). Mutation data combined from Foster and colleagues [64] and Wei and colleagues [65] for a total of 2,577 CDS mutations annotated respectively to the reference *E. coli* genomes NC_000913.2 and NC_000913.3. For GC content, CpG deviation and relative position from the origin, weighted means were used taking into account the proportion of CDS mutations contributed by each data set to the merged CDS mutation data (Foster: 0.65, Wei: 0.35). Stars represent *P*-value (*P* < 0.0001 **** *P* < 0.001 *** *P* < 0.01 ** *P* < 0.05 * *P* > 0.05 no stars). The data and code needed to reproduce this Table can be found in https://doi.org/10.5281/zenodo.23263169.
(CSV)

S21 Table Full (top) and partial (bottom) Kendall correlation analysis between mutation density and all annotated gene features. Mutation density (“mut_density”) determined as mutation count per gene normalised by gene length. Features include GC content (“GC”), deviation of CpG content from that expected by mononucleotide content (“CpG_dev”), relative position from origin (“rel_pos”), gene essentiality classification from the PEC database [77] (“nonessentiality”, scored as 2 for non-essential and 1 for essential), strand orientation relative to the origin (“strand”, scored as 0 for lagging strand, 1 for leading strand), protein abundance from PaxDB [78] only for those available sources conducting the experiment in LB medium [103,104] (“LB_abundance”), and median transcript abundance from a compendium of RNA-seq data [79] (“RNA”). Mutation data combined from Foster and colleagues [64] and Wei and colleagues [65] for a total of 2,577 CDS mutations annotated respectively to the reference *E. coli* genomes NC_000913.2 and NC_000913.3. For GC content, CpG deviation and relative position from the origin, weighted means were used taking into account the proportion of CDS mutations contributed by each data set to the merged CDS mutation data (Foster: 0.65, Wei: 0.35). Stars represent *P*-value (*P* < 0.0001 **** *P* < 0.001 *** *P* < 0.01 ** *P* < 0.05 * *P* > 0.05 no stars). The data and code needed to reproduce this Table can be found in https://doi.org/10.5281/zenodo.23263169.
(CSV)

S22 Table Comprehensive list of all *S. cerevisiae* annotated genomic sequences with respective annotated features and mutation counts. Mutation data combined from four MA experiments [90–93]. All annotations are based on the *Saccharomyces cerevisiae S288C* (available on the Saccharomyces Genome Database). Table includes gene identifiers (locus tag, gene name), genomic position (chromosome, start:end for each exon separately and for the whole gene), strand orientation, gene length and CDS length (in base pairs, bp), GC content, CpG content, deviation of CpG from expected based on mononucleotide frequencies (all three for the whole gene or for CDS only), essentiality score (“KO_E1_NE2_UNK3”: following the Yeast Phenome Database classification [95]: essential = 1, non-essential = 2, unknown = 3), protein abundance (values from PaxDB [78], both raw “abundance” and log10 transformed “Log10abundance”), transcript abundance (“rpkm”, values from the NCBI GEO Database [94]), KaKs values derived from analysis of single-copy genes in *S. cerevisiae* with one to one orthologs across three close relatives (*S. cerevisiae*, *Saccharomyces paradoxus* and *Saccharomyces mikatae IFO 1815* as outgroup) performed by Rivas-Santisteban and colleagues [97], and gene replication times interpolated using data from Berners-Lee and colleagues [96] (“avg_reptime”). Mutation counts (“mut_cnt”) are adjusted for overlapping genes, such that a mutation occurring in two overlapping genes contributes 0.5 to each, and mutation density (“mut_density”) determined as mutation count per gene divided by whole gene length. The data and code needed to reproduce this Table can be found in https://doi.org/10.5281/zenodo.23263169.
(CSV)

S1 Data Correlation analysis between mutation density and annotated gene features, for *Streptococcus pneumoniae D39.* For both sheets A and B, table is split as full (top) and partial (bottom) Kendall correlation analysis. Mutation density (“mut_density”) determined as mutation count per gene normalised by gene length. Features include gene length (“gene_len”), GC content (“GC”), deviation of CpG content from that expected by mononucleotide content (“CpG_dev”), relative position from origin (“rel_pos”), strand orientation relative to the origin (“strand”, scored as 0 for lagging strand, 1 for leading strand), and average transcript levels [111] (“RNA_avg”). For sheet B, gene length is excluded as a covariate. Mutation data and transcript abundance data for WT samples from Jiang and colleagues [111]. Stars represent *P*-value (*P* < 0.0001 **** *P* < 0.001 *** *P* < 0.01 ** *P* < 0.05 * *P* > 0.05 no stars). The data and code needed to reproduce this file can be found in https://doi.org/10.5281/zenodo.23263169.
(XLSX)

S2 Data Correlation analysis between mutation density and annotated gene features, for *Mycobacterium smegmatis MC2 155.* For both sheets A and B, table is split as full (top) and partial (bottom) Kendall correlation analysis. Mutation density (“mut_density”) determined as mutation count per gene normalised by gene length. Features include gene length (“gene_len”), GC content (“GC”), deviation of CpG content from that expected by mononucleotide content (“CpG_dev”), relative position from origin (“rel_pos”), strand orientation relative to the origin (“strand”, scored as 0 for lagging strand, 1 for leading strand) and average transcript levels across the growth phase [114] (“RNA_avg”). For sheet B, gene length is excluded as a covariate. Mutation data for WT samples from Kucukyildirim and colleagues [88] and Castañeda-García and colleagues [89], transcript abundance data from Li and colleagues [114]. Stars represent *P*-value (*P* < 0.0001 **** *P* < 0.001 *** *P* < 0.01 ** *P* < 0.05 * *P* > 0.05 no stars). The data and code needed to reproduce this file can be found in https://doi.org/10.5281/zenodo.23263169.
(XLSX)

S3 Data Correlation analysis between mutation density and annotated gene features, for *Saccharomyces cerevisiae.* For both sheets A and B, table is split as full (top) and partial (bottom) Kendall correlation analysis. Mutation density (“mut_density”) determined as mutation count per gene normalised by gene length. Features include gene length (“gene_length”), GC content (“gene_GC”), deviation of CpG content from that expected by mononucleotide content (“gene_CpG_dev”), transcript levels from the NCBI GEO Database [94] (“rpkm”), protein abundance from PaxDB [78] (“abundance”), gene essentiality classification from the Yeast Phenome Database [95] (“KO_E1_NE2_UNK3”), and gene replication times interpolated using data from Berners-Lee and colleagues [96] (“avg_reptime”). For sheet B, gene length is excluded as a covariate. For both, mutation data merged from four MA experiments [90–93]. Stars represent *P*-value (*P* < 0.0001 **** *P* < 0.001 *** *P* < 0.01 ** *P* < 0.05 * *P* > 0.05 no stars). The data and code needed to reproduce this file can be found in https://doi.org/10.5281/zenodo.23263169.
(XLSX)

S1 Text Summary of statistical basis performed by prior analyses. We summarise the variable tested, what claim is made as regards to it and what method was used to test it. References are included for each. The data and code needed to reproduce this file can be found in https://doi.org/10.5281/zenodo.23263169.
(DOCX)

S2 Text Tests and generalised linear model outputs for mutation count analyses for *E. coli* and *S. cerevisiae.* R outputs are displayed for consideration of mutation data predictors. For *E. coli*, mutation data is combined from Wei and colleagues [65] and Foster and colleagues [64]. All annotations are based on the *E. coli* K-12 MG1655 reference genome (for Foster data version NC_000913.2 and for Wei data NC_000913.3, both available on NCBI [108]). For *S. cerevisiae*, mutation data is combined from four MA experiments [90–93]. All annotations are based on the *Saccharomyces cerevisiae S288C* (available on the Saccharomyces Genome Database). For both organisms, mutation counts are adjusted for overlapping genes, such that a mutation occurring in two overlapping genes contributes 0.5 to each. For *E. coli*, the full models consider the following potential predictors: log-transformed gene length (“gene_len”), GC content (“GC”), deviation of CpG content from that expected by mononucleotide content (“CpG_dev”), relative position from origin (“rel_pos”), gene essentiality classification from the PEC database [77] (“nonessentiality”, scored as 2 for non-essential and 1 for essential), strand orientation relative to the origin (“strand”, scored as 0 for lagging strand, 1 for leading strand), protein abundance from PaxDB [78] (“prot_abundance”), and median transcript abundance from a compendium of RNA-seq data [79] (“RNAmedian_zero”). For *S. cerevisiae*, the full models include log-transformed gene length (“gene_length”), GC content (“CDS_GC”), deviation of CpG content from that expected by mononucleotide content (“gene_CpG_dev”), transcript levels from the NCBI GEO Database [94] (“rpkm”), protein abundance from PaxDB [78] (“abundance”), gene essentiality classification from the Yeast Phenome Database [95] (“KO_E1_NE2_UNK3”), and gene replication times interpolated using data from Berners-Lee and colleagues [96] (“avg_reptime”). For the adaptive models: we consider protein expression, essentiality and strand for *E. coli,* and protein expression and essentiality for *S. cerevisiae*. For *E. coli*, we also consider expression data under LB conditions, including protein abundance from PaxDB [78] restricted to only experiments conducted in LB medium (“LB_protein”), and transcript abundance from an experiment conducted in LB medium by Martincorena and colleagues [1] (“LB_transcripts_Martincorena_early”). For all models, expression data is considered both log-transformed and untransformed. Outputs include a range of testing: Poisson regression followed by overdispersion tests (using “dispersiontest” from the AER package) to select whether to a Poisson or negative binomial regression is more appropriate, residual analysis to test for zero-inflation of the negative binomial models (using “check_zeroinflation” from R package performance), and calculations of Nagelkerke [106] pseudo *R*^2^ (from R package fmsb). All results are qualitatively the same if non-integer mutation counts (introduced to control for mutations occurring in overlapping genes) are removed. Full scripts are available in the public repository as described in the Methods section. The data and code needed to reproduce this file can be found in https://doi.org/10.5281/zenodo.23263169.
(DOCX)

## Abbreviations

bp: base pair
CDS: coding sequence
KO: knockout
MA: mutation accumulation
MMR: mismatch repair
PEC: profiling Escerichia coli chromosome database
SEM: standard error of the mean
SGD: Saccharomyces Genome Database
SNP: single-nucleotide polymorphism
WT: wild-type

## References

[1] Martincorena I, Seshasayee ASN, Luscombe NM. Evidence of non-random mutation rates suggests an evolutionary risk management strategy. Nature. 2012;485(7396):95–8. doi: 10.1038/nature10995 22522932

[2] Monroe JG, Srikant T, Carbonell-Bejerano P, Becker C, Lensink M, Exposito-Alonso M, et al. Mutation bias reflects natural selection in *Arabidopsis thaliana*. Nature. 2022;602(7895):101–5. doi: 10.1038/s41586-021-04269-6 35022609 PMC8810380

[3] Paul S, Million-Weaver S, Chattopadhyay S, Sokurenko E, Merrikh H. Accelerated gene evolution through replication-transcription conflicts. Nature. 2013;495(7442):512–5. doi: 10.1038/nature11989 23538833 PMC3807732

[4] Cairns J, Overbaugh J, Miller S. The origin of mutants. Nature. 1988;335(6186):142–5. doi: 10.1038/335142a0 3045565

[5] Melamed D, Nov Y, Malik A, Yakass MB, Bolotin E, Shemer R, et al. De novo mutation rates at the single-mutation resolution in a human HBB gene region associated with adaptation and genetic disease. Genome Res. 2022;32(3):488–98. doi: 10.1101/gr.276103.121 35031571 PMC8896469

[6] Stoltzfus A. Mutation, randomness, and evolution. Oxford and New York: Oxford University Press; 2023.

[7] Tenaillon O, Denamur E, Matic I. Evolutionary significance of stress-induced mutagenesis in bacteria. Trends Microbiol. 2004;12(6):264–70. doi: 10.1016/j.tim.2004.04.002 15165604

[8] Hodgkinson A, Eyre-Walker A. Variation in the mutation rate across mammalian genomes. Nat Rev Genet. 2011;12(11):756–66. doi: 10.1038/nrg3098 21969038

[9] Hara Y, Kuraku S. Intragenomic mutational heterogeneity: Structural and functional insights from gene evolution. Trends Genet. 2025;41(11):974–83. doi: 10.1016/j.tig.2025.03.007 40328580

[10] Miyata T, Hayashida H, Kuma K, Mitsuyasu K, Yasunaga T. Male-driven molecular evolution: A model and nucleotide sequence analysis. Cold Spring Harb Symp Quant Biol. 1987;52:863–7. doi: 10.1101/sqb.1987.052.01.094 3454295

[11] Schroeder JW, Yeesin P, Simmons LA, Wang JD. Sources of spontaneous mutagenesis in bacteria. Crit Rev Biochem Mol Biol. 2018;53(1):29–48. doi: 10.1080/10409238.2017.1394262 29108429 PMC5975382

[12] Cox EC. On the organization of higher chromosomes. Nat New Biol. 1972;239(92):133–4. doi: 10.1038/newbio239133a0 4633814

[13] Chuang JH, Li H. Functional bias and spatial organization of genes in mutational hot and cold regions in the human genome. PLoS Biol. 2004;2(2):E29. doi: 10.1371/journal.pbio.0020029 14966531 PMC340940

[14] Million-Weaver S, Samadpour AN, Moreno-Habel DA, Nugent P, Brittnacher MJ, Weiss E, et al. An underlying mechanism for the increased mutagenesis of lagging-strand genes in *Bacillus subtilis*. Proc Natl Acad Sci U S A. 2015;112(10):E1096-105. doi: 10.1073/pnas.1416651112 25713353 PMC4364195

[15] Galhardo RS, Hastings PJ, Rosenberg SM. Mutation as a stress response and the regulation of evolvability. Crit Rev Biochem Mol Biol. 2007;42(5):399–435. doi: 10.1080/10409230701648502 17917874 PMC3319127

[16] Merrikh H. Spatial and temporal control of evolution through replication-transcription conflicts. Trends Microbiol. 2017;25(7):515–21. doi: 10.1016/j.tim.2017.01.008 28216294 PMC5474121

[17] Rocha EPC, Danchin A. Gene essentiality determines chromosome organisation in bacteria. Nucleic Acids Res. 2003;31(22):6570–7. doi: 10.1093/nar/gkg859 14602916 PMC275555

[18] Rocha EPC, Danchin A. Essentiality, not expressiveness, drives gene-strand bias in bacteria. Nat Genet. 2003;34(4):377–8. doi: 10.1038/ng1209 12847524

[19] Chen X, Zhang J. No gene-specific optimization of mutation rate in *Escherichia coli*. Mol Biol Evol. 2013;30(7):1559–62. doi: 10.1093/molbev/mst060 23533222 PMC3684851

[20] Liu HX, Zhang JZ. Is the mutation rate lower in genomic regions of stronger selective constraints? Mol Biol Evol. 2022;39(8). doi: 10.1093/molbev/msac169 WOS:000839630100005 PMC9372563 35907247

[21] Wang L, Ho AT, Hurst LD, Yang S. Re-evaluating evidence for adaptive mutation rate variation. Nature. 2023;619(7971):E52–6. doi: 10.1038/s41586-023-06314-y 37495884 PMC10371861

[22] McVean GT, Hurst LD. Evidence for a selectively favourable reduction in the mutation rate of the X chromosome. Nature. 1997;386(6623):388–92. doi: 10.1038/386388a0 9121553

[23] Majic P, Payne JL. Developmental selection and the perception of mutation bias. Mol Biol Evol. 2023;40(8):msad179. doi: 10.1093/molbev/msad179 37556606 PMC10443735

[24] Vigué L, Tenaillon O. Predicting the effect of mutations to investigate recent events of selection across 60,472 *Escherichia coli* strains. Proc Natl Acad Sci U S A. 2023;120(31):e2304177120. doi: 10.1073/pnas.2304177120 37487088 PMC10401003

[25] Eyre-Walker A. Recombination and mammalian genome evolution. Proc Biol Sci. 1993;252(1335):237–43. doi: 10.1098/rspb.1993.0071 8394585

[26] Long H, Sung W, Kucukyildirim S, Williams E, Miller SF, Guo W, et al. Evolutionary determinants of genome-wide nucleotide composition. Nat Ecol Evol. 2018;2(2):237–40. doi: 10.1038/s41559-017-0425-y 29292397 PMC6855595

[27] Charlesworth B, Jensen JD. Population genetic considerations regarding evidence for biased mutation rates in *Arabidopsis thaliana*. Mol Biol Evol. 2023;40(2):msac275. doi: 10.1093/molbev/msac275 36572441 PMC9907473

[28] Radrizzani S, Rivas-Santisteban J, Han N, Hurst LD. Bacterial gene 5’ ends have unusual mutation rates that can mislead tests of selection. PLoS Biol. 2025;23(12):e3003569. doi: 10.1371/journal.pbio.3003569 41397037 PMC12725619

[29] Nachman MW, Crowell SL. Estimate of the mutation rate per nucleotide in humans. Genetics. 2000;156(1):297–304. doi: 10.1093/genetics/156.1.297 10978293 PMC1461236

[30] Lynch M. Evolution of the mutation rate. Trends Genet. 2010;26(8):345–52. doi: 10.1016/j.tig.2010.05.003 20594608 PMC2910838

[31] Xia X, Xie Z, Li W-H. Effects of GC content and mutational pressure on the lengths of exons and coding sequences. J Mol Evol. 2003;56(3):362–70. doi: 10.1007/s00239-002-2406-1 12612839

[32] Pink CJ, Hurst LD. Timing of replication is a determinant of neutral substitution rates but does not explain slow Y chromosome evolution in rodents. Mol Biol Evol. 2010;27(5):1077–86. doi: 10.1093/molbev/msp314 20026481

[33] Chen C-L, Rappailles A, Duquenne L, Huvet M, Guilbaud G, Farinelli L, et al. Impact of replication timing on non-CpG and CpG substitution rates in mammalian genomes. Genome Res. 2010;20(4):447–57. doi: 10.1101/gr.098947.109 20103589 PMC2847748

[34] Lang GI, Murray AW. Mutation rates across budding yeast chromosome VI are correlated with replication timing. Genome Biol Evol. 2011;3:799–811. doi: 10.1093/gbe/evr054 21666225 PMC3170098

[35] Stamatoyannopoulos JA, Adzhubei I, Thurman RE, Kryukov GV, Mirkin SM, Sunyaev SR. Human mutation rate associated with DNA replication timing. Nat Genet. 2009;41(4):393–5. doi: 10.1038/ng.363 19287383 PMC2914101

[36] Tomkova M, Tomek J, Kriaucionis S, Schuster-Böckler B. Mutational signature distribution varies with DNA replication timing and strand asymmetry. Genome Biol. 2018;19(1):129. doi: 10.1186/s13059-018-1509-y 30201020 PMC6130095

[37] Weber CC, Pink CJ, Hurst LD. Late-replicating domains have higher divergence and diversity in *Drosophila melanogaster*. Mol Biol Evol. 2012;29(2):873–82. doi: 10.1093/molbev/msr265 22046001

[38] Koren A, Polak P, Nemesh J, Michaelson JJ, Sebat J, Sunyaev SR, et al. Differential relationship of DNA replication timing to different forms of human mutation and variation. Am J Hum Genet. 2012;91(6):1033–40. doi: 10.1016/j.ajhg.2012.10.018 23176822 PMC3516607

[39] Agier N, Fischer G. The mutational profile of the yeast genome is shaped by replication. Mol Biol Evol. 2012;29(3):905–13. doi: 10.1093/molbev/msr280 22114361

[40] Lang GI, Parsons L, Gammie AE. Mutation rates, spectra, and genome-wide distribution of spontaneous mutations in mismatch repair deficient yeast. G3 (Bethesda). 2013;3(9):1453–65. doi: 10.1534/g3.113.006429 23821616 PMC3755907

[41] Wolfe KH, Sharp PM, Li WH. Mutation rates differ among regions of the mammalian genome. Nature. 1989;337(6204):283–5. doi: 10.1038/337283a0 2911369

[42] Dillon MM, Sung W, Lynch M, Cooper VS. Periodic variation of mutation rates in bacterial genomes associated with replication timing. mBio. 2018;9(4):e01371-18. doi: 10.1128/mBio.01371-18 30131359 PMC6106087

[43] Lato DF, Golding GB. Spatial patterns of gene expression in bacterial genomes. J Mol Evol. 2020;88(6):510–20. doi: 10.1007/s00239-020-09951-3 32506154 PMC7324424

[44] Niccum BA, Lee H, MohammedIsmail W, Tang H, Foster PL. The symmetrical wave pattern of base-pair substitution rates across the *Escherichia coli* chromosome has multiple causes. mBio. 2019;10(4):e01226-19. doi: 10.1128/mBio.01226-19 31266871 PMC6606806

[45] Datta A, Jinks-Robertson S. Association of increased spontaneous mutation rates with high levels of transcription in yeast. Science. 1995;268(5217):1616–9. doi: 10.1126/science.7777859 7777859

[46] Park C, Qian W, Zhang J. Genomic evidence for elevated mutation rates in highly expressed genes. EMBO Rep. 2012;13(12):1123–9. doi: 10.1038/embor.2012.165 23146897 PMC3512408

[47] Lind PA, Andersson DI. Whole-genome mutational biases in bacteria. Proc Natl Acad Sci U S A. 2008;105(46):17878–83. doi: 10.1073/pnas.0804445105 19001264 PMC2584707

[48] Chen X, Zhang J. Yeast mutation accumulation experiment supports elevated mutation rates at highly transcribed sites. Proc Natl Acad Sci U S A. 2014;111(39):E4062. doi: 10.1073/pnas.1412284111 25217566 PMC4191789

[49] Zhu YO, Siegal ML, Hall DW, Petrov DA. Reply to Chen and Zhang: On interpreting genome-wide trends from yeast mutation accumulation data. Proc Natl Acad Sci U S A. 2014;111(39):E4063. doi: 10.1073/pnas.1413861111 25217565 PMC4191808

[50] Cho J-E, Jinks-Robertson S. Ribonucleotides and transcription-associated mutagenesis in yeast. J Mol Biol. 2017;429(21):3156–67. doi: 10.1016/j.jmb.2016.08.005 27511624 PMC5296399

[51] Xia B, Yan Y, Baron M, Wagner F, Barkley D, Chiodin M, et al. Widespread transcriptional scanning in the testis modulates gene evolution rates. Cell. 2020;180(2):248-262.e21. doi: 10.1016/j.cell.2019.12.015 31978344 PMC7891839

[52] Polak P, Arndt PF. Transcription induces strand-specific mutations at the 5’ end of human genes. Genome Res. 2008;18(8):1216–23. doi: 10.1101/gr.076570.108 18463301 PMC2493432

[53] Foster PL, Niccum BA, Lee H. DNA replication-transcription conflicts do not significantly contribute to spontaneous mutations due to replication errors in *Escherichia coli*. mBio. 2021;12(5):e0250321. doi: 10.1128/mBio.02503-21 34634932 PMC8510543

[54] Lynch M, Ackerman MS, Gout J-F, Long H, Sung W, Thomas WK, et al. Genetic drift, selection and the evolution of the mutation rate. Nat Rev Genet. 2016;17(11):704–14. doi: 10.1038/nrg.2016.104 27739533

[55] Schroeder JW, Hirst WG, Szewczyk GA, Simmons LA. The effect of local sequence context on mutational bias of genes encoded on the leading and lagging strands. Curr Biol. 2016;26(5):692–7. doi: 10.1016/j.cub.2016.01.016 26923786 PMC4783269

[56] Monroe JG, Murray KD, Xian W, Srikant T, Carbonell-Bejerano P, Becker C, et al. Reply to: Re-evaluating evidence for adaptive mutation rate variation. Nature. 2023;619(7971):E57–60. doi: 10.1038/s41586-023-06315-x 37495874 PMC10371858

[57] Martincorena I, Luscombe NM. Response to No gene-specific optimization of mutation rate in *Escherichia coli*. arXiv. 2013. doi: 10.48550/arXiv.1305.1436 PMC3684851 23533222

[58] Lee H, Popodi E, Tang H, Foster PL. Rate and molecular spectrum of spontaneous mutations in the bacterium *Escherichia coli* as determined by whole-genome sequencing. Proc Natl Acad Sci U S A. 2012;109(41):E2774-83. doi: 10.1073/pnas.1210309109 22991466 PMC3478608

[59] Waneka G, Pate B, Monroe JG, Sloan DB. Exploring the relationship between gene expression and low-frequency somatic mutations in *Arabidopsis* with duplex sequencing. Genome Biol Evol. 2024;16(10):evae213. doi: 10.1093/gbe/evae213 39365161 PMC11489876

[60] Mellon I, Champe GN. Products of DNA mismatch repair genes mutS and mutL are required for transcription-coupled nucleotide-excision repair of the lactose operon in *Escherichia coli*. Proc Natl Acad Sci U S A. 1996;93(3):1292–7. doi: 10.1073/pnas.93.3.1292 8577757 PMC40073

[61] Mellon I, Hanawalt PC. Induction of the *Escherichia coli* lactose operon selectively increases repair of its transcribed DNA strand. Nature. 1989;342(6245):95–8. doi: 10.1038/342095a0 2554145

[62] Beletskii A, Bhagwat AS. Transcription-induced mutations: Increase in C to T mutations in the nontranscribed strand during transcription in *Escherichia coli*. Proc Natl Acad Sci U S A. 1996;93(24):13919–24. doi: 10.1073/pnas.93.24.13919 8943036 PMC19468

[63] Couturier E, Rocha EPC. Replication-associated gene dosage effects shape the genomes of fast-growing bacteria but only for transcription and translation genes. Mol Microbiol. 2006;59(5):1506–18. doi: 10.1111/j.1365-2958.2006.05046.x 16468991

[64] Foster PL, Lee H, Popodi E, Townes JP, Tang H. Determinants of spontaneous mutation in the bacterium *Escherichia coli* as revealed by whole-genome sequencing. Proc Natl Acad Sci U S A. 2015;112(44):E5990-9. doi: 10.1073/pnas.1512136112 26460006 PMC4640725

[65] Wei W, Ho W-C, Behringer MG, Miller SF, Bcharah G, Lynch M. Rapid evolution of mutation rate and spectrum in response to environmental and population-genetic challenges. Nat Commun. 2022;13(1):4752. doi: 10.1038/s41467-022-32353-6 35963846 PMC9376063

[66] Long H, Miller SF, Strauss C, Zhao C, Cheng L, Ye Z, et al. Antibiotic treatment enhances the genome-wide mutation rate of target cells. Proc Natl Acad Sci U S A. 2016;113(18):E2498-505. doi: 10.1073/pnas.1601208113 27091991 PMC4983809

[67] Sane M, Parveen S, Agashe D. Mutation bias alters the distribution of fitness effects of mutations. PLoS Biol. 2025;23(7):e3003282. doi: 10.1371/journal.pbio.3003282 40658723 PMC12273949

[68] Tincher C, Long H, Behringer M, Walker N, Lynch M. The Glyphosate-based Herbicide Roundup Does Not Elevate Genome-wide Mutagenesis Of *Escherichia coli*. G3 (Bethesda). 2017;7(10):3331–5. doi: 10.1534/g3.117.300133 28983068 PMC5633383

[69] Pál C, Papp B, Hurst LD. Highly expressed genes in yeast evolve slowly. Genetics. 2001;158(2):927–31. doi: 10.1093/genetics/158.2.927 11430355 PMC1461684

[70] Zhang J, Yang J-R. Determinants of the rate of protein sequence evolution. Nat Rev Genet. 2015;16(7):409–20. doi: 10.1038/nrg3950 26055156 PMC4523088

[71] Schüler A, Ghanbarian AT, Hurst LD. Purifying selection on splice-related motifs, not expression level nor RNA folding, explains nearly all constraint on human lincRNAs. Mol Biol Evol. 2014;31(12):3164–83. doi: 10.1093/molbev/msu249 25158797 PMC4245815

[72] Wahl LM, Agashe D. Selection bias in mutation accumulation. Evolution. 2022;76(3):528–40. doi: 10.1111/evo.14430 34989408

[73] Mahilkar A, Raj N, Kemkar S, Saini S. Selection in a growing colony biases results of mutation accumulation experiments. Sci Rep. 2022;12(1):15470. doi: 10.1038/s41598-022-19928-5 36104390 PMC9475022

[74] Jago MJ, Green R, Czernuszka MR, Denisov S, Krašovec R, Knight CG, et al. Extended sequence context shapes mutational bias in *Escherichia coli*. Proc Natl Acad Sci U S A. 2026;123(23):e2601345123. doi: 10.1073/pnas.2601345123 42234524 PMC13250602

[75] Haig D, Hurst LD. A quantitative measure of error minimization in the genetic code. J Mol Evol. 1991;33(5):412–7. doi: 10.1007/BF02103132 1960738

[76] Kendall MG, Dickinson Gibbons J. Rank correlation methods. 5th ed. New York, NY: Oxford University Press; 1990.

[77] Hashimoto M, Ichimura T, Mizoguchi H, Tanaka K, Fujimitsu K, Keyamura K, et al. Cell size and nucleoid organization of engineered *Escherichia coli* cells with a reduced genome. Mol Microbiol. 2005;55(1):137–49. doi: 10.1111/j.1365-2958.2004.04386.x 15612923

[78] Huang Q, Szklarczyk D, Wang M, Simonovic M, von Mering C. PaxDb 5.0: Curated Protein Quantification Data Suggests Adaptive Proteome Changes In Yeasts. Mol Cell Proteomics. 2023;22(10):100640. doi: 10.1016/j.mcpro.2023.100640 37659604 PMC10551891

[79] Tjaden B. *Escherichia coli* transcriptome assembly from a compendium of RNA-seq data sets. RNA Biol. 2023;20(1):77–84. doi: 10.1080/15476286.2023.2189331 36920168 PMC10392735

[80] Feugeas J-P, Tourret J, Launay A, Bouvet O, Hoede C, Denamur E, et al. Links Between Transcription, Environmental Adaptation And Gene Variability in *Escherichia coli*: Correlations between Gene Expression And Gene Variability Reflect Growth Efficiencies. Mol Biol Evol. 2016;33(10):2515–29. doi: 10.1093/molbev/msw105 27352853

[81] Scholz SA, Diao R, Wolfe MB, Fivenson EM, Lin XN, Freddolino PL. High-Resolution Mapping of the *Escherichia coli* Chromosome Reveals Positions Of High And Low Transcription. Cell Syst. 2019;8(3):212-225.e9. doi: 10.1016/j.cels.2019.02.004 30904377 PMC6508686

[82] Hanawalt PC, Spivak G. Transcription-coupled DNA repair: two decades of progress and surprises. Nat Rev Mol Cell Biol. 2008;9(12):958–70. doi: 10.1038/nrm2549 19023283

[83] Klapacz J, Bhagwat AS. Transcription-dependent increase in multiple classes of base substitution mutations in *Escherichia coli*. J Bacteriol. 2002;184(24):6866–72. doi: 10.1128/JB.184.24.6866-6872.2002 12446637 PMC135453

[84] Foster PL, Niccum BA, Popodi E, Townes JP, Lee H, MohammedIsmail W, et al. Determinants of Base-pair Substitution Patterns Revealed By Whole-genome Sequencing Of DNA mismatch repair defective *Escherichia coli*. Genetics. 2018;209(4):1029–42. doi: 10.1534/genetics.118.301237 29907647 PMC6063221

[85] Ewens WJ. On estimating P values by the Monte Carlo method. Am J Hum Genet. 2003;72(2):496–8. doi: 10.1086/346174 12596794 PMC529334

[86] Lynch M, Ali F, Lin T, Wang Y, Ni J, Long H. The divergence of mutation rates and spectra across the Tree of Life. EMBO Rep. 2023;24(10):e57561. doi: 10.15252/embr.202357561 37615267 PMC10561183

[87] Hale OF, Yin M, Behringer MG. Elevated rates and biased spectra of mutations in anaerobically cultured lactic acid bacteria. mBio. 2025;16(12):e0305425. doi: 10.1128/mbio.03054-25 41369254 PMC12691593

[88] Kucukyildirim S, Long H, Sung W, Miller SF, Doak TG, Lynch M. The rate and spectrum of spontaneous mutations in *Mycobacterium smegmatis*, a bacterium naturally devoid of the postreplicative mismatch repair pathway. G3 (Bethesda). 2016;6(7):2157–63. doi: 10.1534/g3.116.030130 27194804 PMC4938668

[89] Castañeda-García A, Martín-Blecua I, Cebrián-Sastre E, Chiner-Oms A, Torres-Puente M, Comas I, et al. Specificity and mutagenesis bias of the mycobacterial alternative mismatch repair analyzed by mutation accumulation studies. Sci Adv. 2020;6(7):eaay4453. doi: 10.1126/sciadv.aay4453 32095527 PMC7015689

[90] Zhu YO, Siegal ML, Hall DW, Petrov DA. Precise estimates of mutation rate and spectrum in yeast. Proc Natl Acad Sci U S A. 2014;111(22):E2310-8. doi: 10.1073/pnas.1323011111 24847077 PMC4050626

[91] Sharp NP, Sandell L, James CG, Otto SP. The genome-wide rate and spectrum of spontaneous mutations differ between haploid and diploid yeast. Proc Natl Acad Sci U S A. 2018;115(22):E5046–55. doi: 10.1073/pnas.1801040115 29760081 PMC5984525

[92] Liu H, Zhang J. Yeast spontaneous mutation rate and spectrum vary with environment. Curr Biol. 2019;29(10):1584-1591.e3. doi: 10.1016/j.cub.2019.03.054 31056389 PMC6529271

[93] Sui Y, Qi L, Wu J-K, Wen X-P, Tang X-X, Ma Z-J, et al. Genome-wide mapping of spontaneous genetic alterations in diploid yeast cells. Proc Natl Acad Sci U S A. 2020;117(45):28191–200. doi: 10.1073/pnas.2018633117 33106417 PMC7668089

[94] Clough E, Barrett T, Wilhite SE, Ledoux P, Evangelista C, Kim IF, et al. NCBI GEO: Archive for gene expression and epigenomics data sets: 23-year update. Nucleic Acids Res. 2024;52(D1):D138–44. doi: 10.1093/nar/gkad965 37933855 PMC10767856

[95] Turco G, Chang C, Wang RY, Kim G, Stoops EH, Richardson B, et al. Global analysis of the yeast knockout phenome. Sci Adv. 2023;9(21):eadg5702. doi: 10.1126/sciadv.adg5702 37235661 PMC11326039

[96] Berners-Lee R, Gilmore E, Berkemeier F, Boemo MA. Regulation of replication timing in *Saccharomyces cerevisiae*. PLoS Comput Biol. 2025;21(6):e1013066. doi: 10.1371/journal.pcbi.1013066 40455924 PMC12165382

[97] Rivas-Santisteban J, Yubero P, Hurst LD. Cell compartment is a predictor of protein rate of evolution, but not in the manner expected: Evidence against the extended complexity hypothesis. Genome Biol Evol. 2025;17(7):evaf126. doi: 10.1093/gbe/evaf126 40580929 PMC12271741

[98] Pich O, Muiños F, Sabarinathan R, Reyes-Salazar I, Gonzalez-Perez A, Lopez-Bigas N. Somatic and germline mutation periodicity follow the orientation of the DNA minor groove around nucleosomes. Cell. 2018;175(4):1074-1087.e18. doi: 10.1016/j.cell.2018.10.004 30388444

[99] Chen X, Yang J-R, Zhang J. Nascent RNA folding mitigates transcription-associated mutagenesis. Genome Res. 2016;26(1):50–9. doi: 10.1101/gr.195164.115 26518484 PMC4691750

[100] Yu G, Liu Y, Li Z, Deng S, Wu Z, Zhang X, et al. Genome-wide probing of eukaryotic nascent RNA structure elucidates cotranscriptional folding and its antimutagenic effect. Nat Commun. 2023;14(1):5853. doi: 10.1038/s41467-023-41550-w 37730811 PMC10511511

[101] Hasenauer FC, Barreto HC, Lotton C, Matic I. Genome-wide mapping of spontaneous DNA replication error-hotspots using mismatch repair proteins in rapidly proliferating *Escherichia coli*. Nucleic Acids Res. 2025;53(2):gkae1196. doi: 10.1093/nar/gkae1196 39660654 PMC11754648

[102] Fryxell KJ, Zuckerkandl E. Cytosine deamination plays a primary role in the evolution of mammalian isochores. Mol Biol Evol. 2000;17(9):1371–83. doi: 10.1093/oxfordjournals.molbev.a026420 10958853

[103] Krug K, Carpy A, Behrends G, Matic K, Soares NC, Macek B. Deep coverage of the *Escherichia coli* proteome enables the assessment of false discovery rates in simple proteogenomic experiments. Mol Cell Proteomics. 2013;12(11):3420–30. doi: 10.1074/mcp.M113.029165 23908556 PMC3820952

[104] Wiśniewski JR, Rakus D. Multi-enzyme digestion FASP and the ‘Total Protein Approach’-based absolute quantification of the *Escherichia coli* proteome. J Proteomics. 2014;109:322–31. doi: 10.1016/j.jprot.2014.07.012 25063446

[105] Drummond DA, Bloom JD, Adami C, Wilke CO, Arnold FH. Why highly expressed proteins evolve slowly. Proc Natl Acad Sci U S A. 2005;102(40):14338–43. doi: 10.1073/pnas.0504070102 16176987 PMC1242296

[106] Nagelkerke NJD. A note on a general definition of the coefficient of determination. Biometrika. 1991;78(3):691–2. doi: 10.1093/biomet/78.3.691

[107] Cox DR, Snell EJ. Analysis of binary data. 2nd ed. Chapman & Hall; 1989.

[108] O’Leary NA, Wright MW, Brister JR, Ciufo S, Haddad D, McVeigh R, et al. Reference sequence (RefSeq) database at NCBI: Current status, taxonomic expansion, and functional annotation. Nucleic Acids Res. 2016;44(D1):D733-45. doi: 10.1093/nar/gkv1189 26553804 PMC4702849

[109] Zhang X, Zhang X, Zhang X, Liao Y, Song L, Zhang Q, et al. Spatial vulnerabilities of the *Escherichia coli* genome to spontaneous mutations revealed with improved duplex sequencing. Genetics. 2018;210(2):547–58. doi: 10.1534/genetics.118.301345 30076202 PMC6216599

[110] Bhawsinghka N, Burkholder A, Schaaper RM. Detection of DNA replication errors and 8-oxo-dGTP-mediated mutations in *E. coli* by Duplex DNA Sequencing. DNA Repair (Amst). 2023;123:103462. doi: 10.1016/j.dnarep.2023.103462 36738688 PMC9992157

[111] Jiang W, Lin T, Pan J, Rivera CE, Tincher C, Wang Y, et al. Spontaneous mutations and mutational responses to penicillin treatment in the bacterial pathogen *Streptococcus pneumoniae* D39. Mar Life Sci Technol. 2024;6(2):198–211. doi: 10.1007/s42995-024-00220-6 38827133 PMC11136922

[112] Choe D, Kim U, Hwang S, Seo SW, Kim D, Cho S, et al. Revealing causes for false-positive and false-negative calling of gene essentiality in *Escherichia coli* using transposon insertion sequencing. mSystems. 2023;8(1):e0089622. doi: 10.1128/msystems.00896-22 36507678 PMC9948719

[113] Kahramanoglou C, Seshasayee ASN, Prieto AI, Ibberson D, Schmidt S, Zimmermann J, et al. Direct and indirect effects of H-NS and Fis on global gene expression control in *Escherichia coli*. Nucleic Acids Res. 2011;39(6):2073–91. doi: 10.1093/nar/gkq934 21097887 PMC3064808

[114] Li XF, Mei H, Chen F, Tang Q, Yu ZQ, Cao XJ. Transcriptome landscape of *Mycobacterium smegmatis*. Front Microbiol. 2017. doi: 10.3389/fmicb.2017.02505 WOS:000418120900001 PMC5741613 29326668

[115] Müller CA, Hawkins M, Retkute R, Malla S, Wilson R, Blythe MJ, et al. The dynamics of genome replication using deep sequencing. Nucleic Acids Res. 2014;42(1):e3. doi: 10.1093/nar/gkt878 24089142 PMC3874191

[116] Siow CC, Nieduszynska SR, Müller CA, Nieduszynski CA. OriDB, the DNA replication origin database updated and extended. Nucleic Acids Res. 2012;40(Database issue):D682-6. doi: 10.1093/nar/gkr1091 22121216 PMC3245157

[117] Engel SR, Dietrich FS, Fisk DG, Binkley G, Balakrishnan R, Costanzo MC, et al. The reference genome sequence of *Saccharomyces cerevisiae*: Then and now. G3 (Bethesda). 2014;4(3):389–98. doi: 10.1534/g3.113.008995 24374639 PMC3962479

[118] Yang Z. PAML 4: Phylogenetic analysis by maximum likelihood. Mol Biol Evol. 2007;24(8):1586–91. doi: 10.1093/molbev/msm088 17483113

