# Supplementary material for: The importance of genes does not predict their mutation rate
Source: PLoS Biol. 2026 Nov 12;24(11):e3004089. doi: 10.1371/journal.pbio.3004089 (PMC13759209; doi:10.1371/journal.pbio.3004089)
Supplement: Supplementary file 1 [file pbio.3004089.s001.pdf]

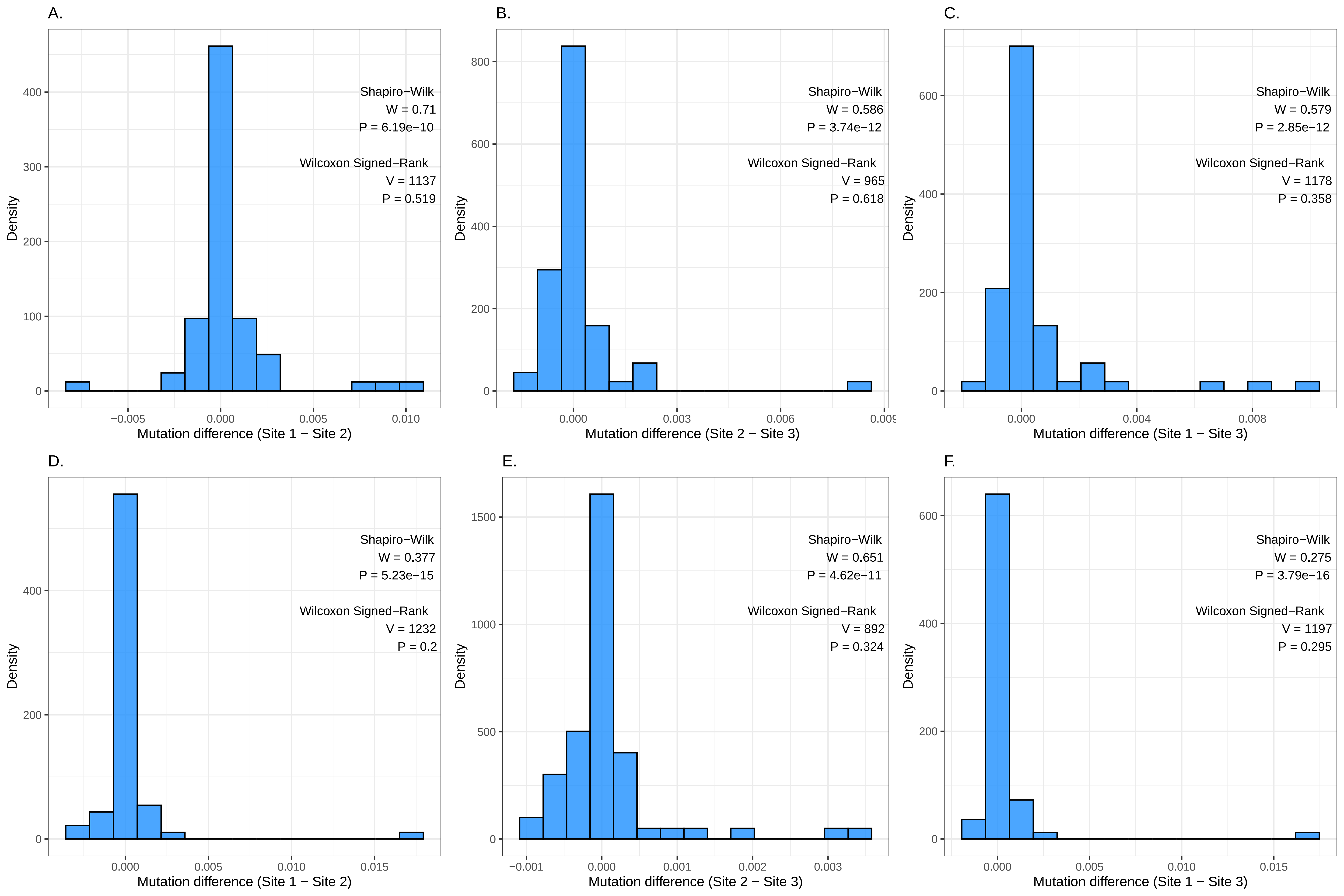
A.
Density
Shapiro−Wilk
W = 0.71
P = 6.19e−10
Wilcoxon Signed−Rank
V = 1137
P = 0.519
Mutation difference (Site 1 − Site 2)
B.
Density
Shapiro−Wilk
W = 0.586
P = 3.74e−12
Wilcoxon Signed−Rank
V = 965
P = 0.618
Mutation difference (Site 2 − Site 3)
C.
Density
Shapiro−Wilk
W = 0.579
P = 2.85e−12
Wilcoxon Signed−Rank
V = 1178
P = 0.358
Mutation difference (Site 1 − Site 3)
D.
Density
Shapiro−Wilk
W = 0.377
P = 5.23e−15
Wilcoxon Signed−Rank
V = 1232
P = 0.2
Mutation difference (Site 1 − Site 2)
E.
Density
Shapiro−Wilk
W = 0.651
P = 4.62e−11
Wilcoxon Signed−Rank
V = 892
P = 0.324
Mutation difference (Site 2 − Site 3)
F.
Density
Shapiro−Wilk
W = 0.275
P = 3.79e−16
Wilcoxon Signed−Rank
V = 1197
P = 0.295
Mutation difference (Site 1 − Site 3)
