# Supplementary material for: The importance of genes does not predict their mutation rate
Source: PLoS Biol. 2026 Nov 12;24(11):e3004089. doi: 10.1371/journal.pbio.3004089 (PMC13759209; doi:10.1371/journal.pbio.3004089)
Supplement: Supplementary file 2 [file pbio.3004089.s002.pdf]

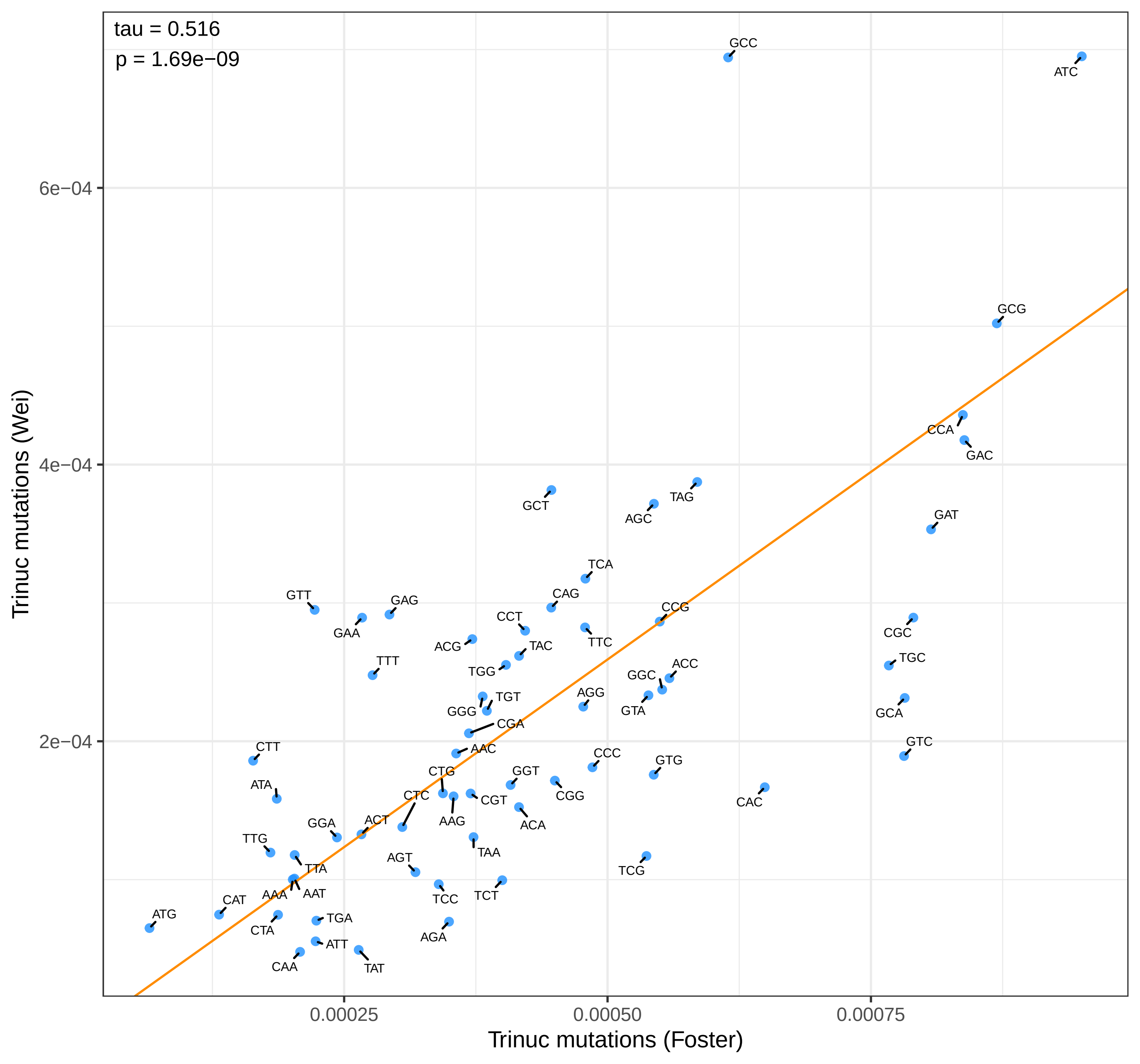

tau = 0.516
p = 1.69e−09
Trinuc mutations (Wei)
Trinuc mutations (Foster)
6e−04
4e−04
2e−04
0.00025
0.00050
0.00075
GCC
ATC
GCG
CCA
GAC
GAT
TAG
AGC
GCT
TCA
CAG
CCG
CGC
TGC
GCA
GTC
GTT
GAG
GAA
CCT
TTC
ACG
TAC
TGG
TTT
ACC
GGC
AGG
GTA
TGT
GGG
CGA
AAC
CCC
GTG
CTT
CTG
GGT
CGG
CAC
ATA
CTC
CGT
AAG
ACA
GGA
ACT
TTG
TAA
AGT
TCG
TTA
AAA
AAT
TCC
TCT
CAT
ATG
CTA
TGA
AGA
ATT
CAA
TAT
