# Supplementary material for: The importance of genes does not predict their mutation rate
Source: PLoS Biol. 2026 Nov 12;24(11):e3004089. doi: 10.1371/journal.pbio.3004089 (PMC13759209; doi:10.1371/journal.pbio.3004089)
Supplement: Supplementary file 3 [file pbio.3004089.s003.pdf]

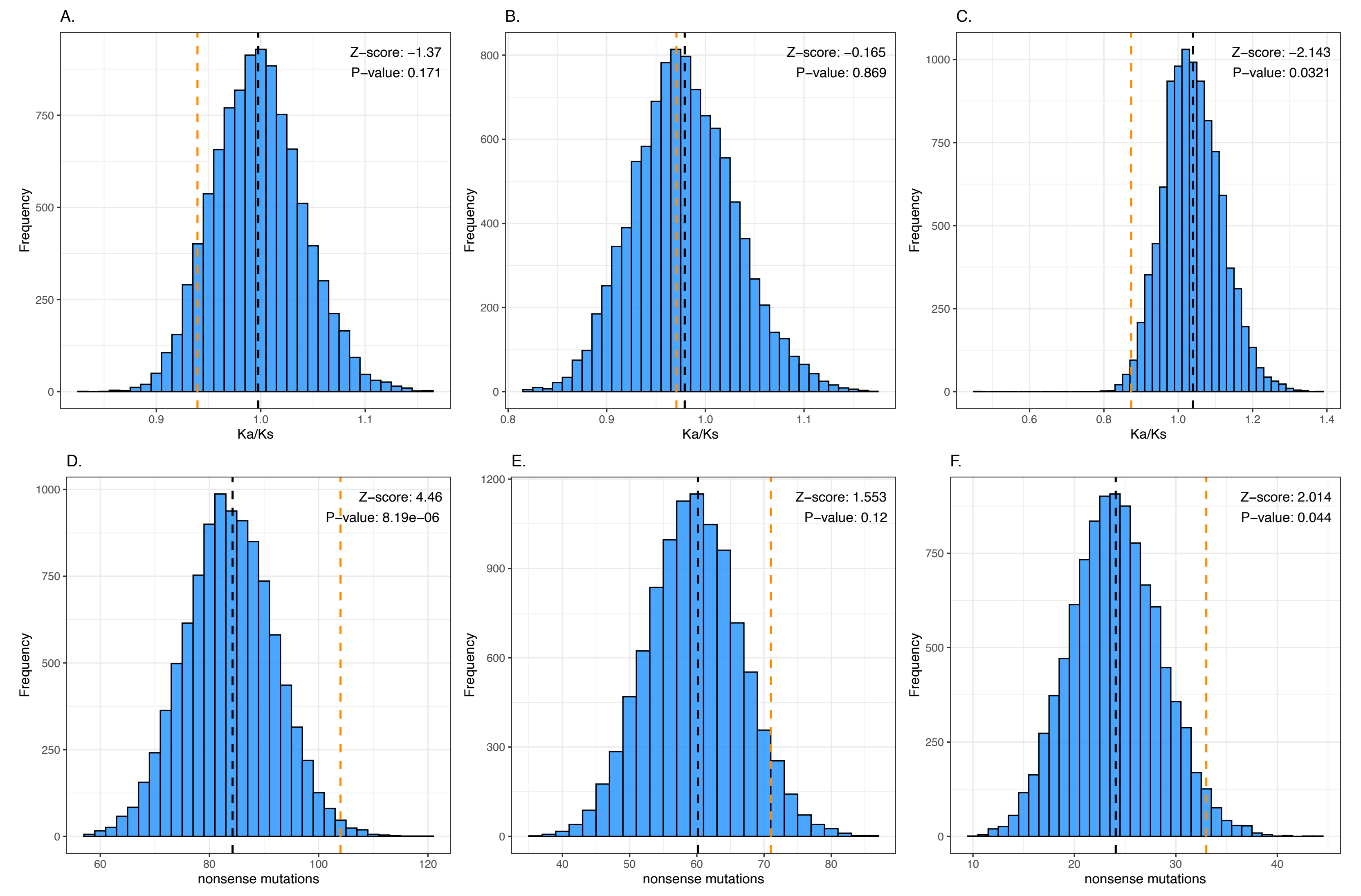
A.
Z–score: −1.37
P–value: 0.171
Frequency
Ka/Ks
0
250
500
750
0.9
1.0
1.1
B.
Z–score: −0.165
P–value: 0.869
Frequency
Ka/Ks
0
200
400
600
800
0.8
0.9
1.0
1.1
C.
Z–score: −2.143
P–value: 0.0321
Frequency
Ka/Ks
0
250
500
750
1000
0.6
0.8
1.0
1.2
1.4
D.
Z–score: 4.46
P–value: 8.19e–06
Frequency
nonsense mutations
0
250
500
750
1000
60
80
100
120
E.
Z–score: 1.553
P–value: 0.12
Frequency
nonsense mutations
0
300
600
900
1200
40
50
60
70
80
F.
Z–score: 2.014
P–value: 0.044
Frequency
nonsense mutations
0
250
500
750
10
20
30
40
