# Supplementary material for: The importance of genes does not predict their mutation rate
Source: PLoS Biol. 2026 Nov 12;24(11):e3004089. doi: 10.1371/journal.pbio.3004089 (PMC13759209; doi:10.1371/journal.pbio.3004089)
Supplement: Supplementary file 4 [file pbio.3004089.s004.pdf]

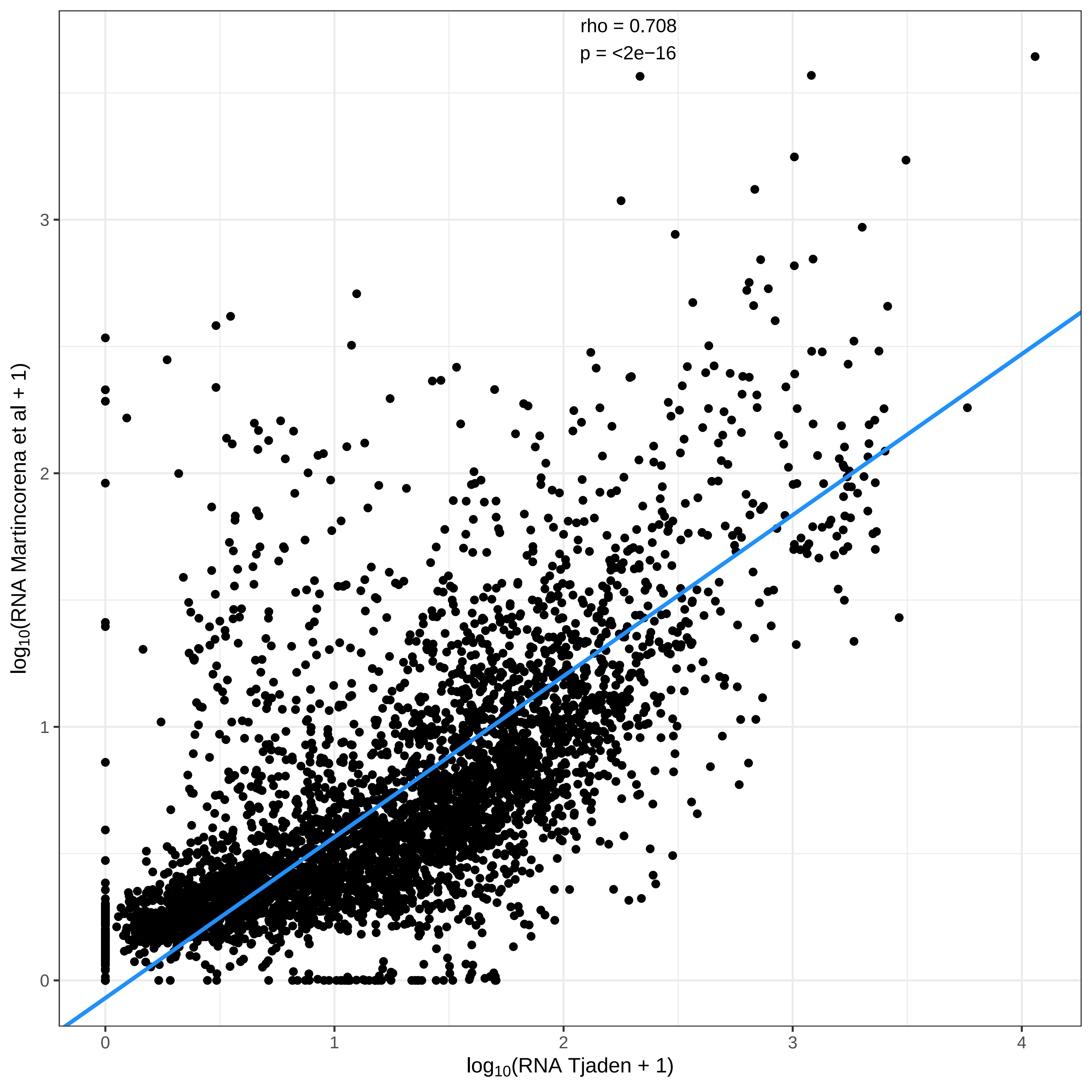

rho = 0.708
p = <2e−16
log10(RNA Martincorena et al + 1)
log10(RNA Tjaden + 1)
0
1
2
3
4
