# Supplementary material for: The importance of genes does not predict their mutation rate
Source: PLoS Biol. 2026 Nov 12;24(11):e3004089. doi: 10.1371/journal.pbio.3004089 (PMC13759209; doi:10.1371/journal.pbio.3004089)
Supplement: Supplementary file 6 [file pbio.3004089.s006.pdf]

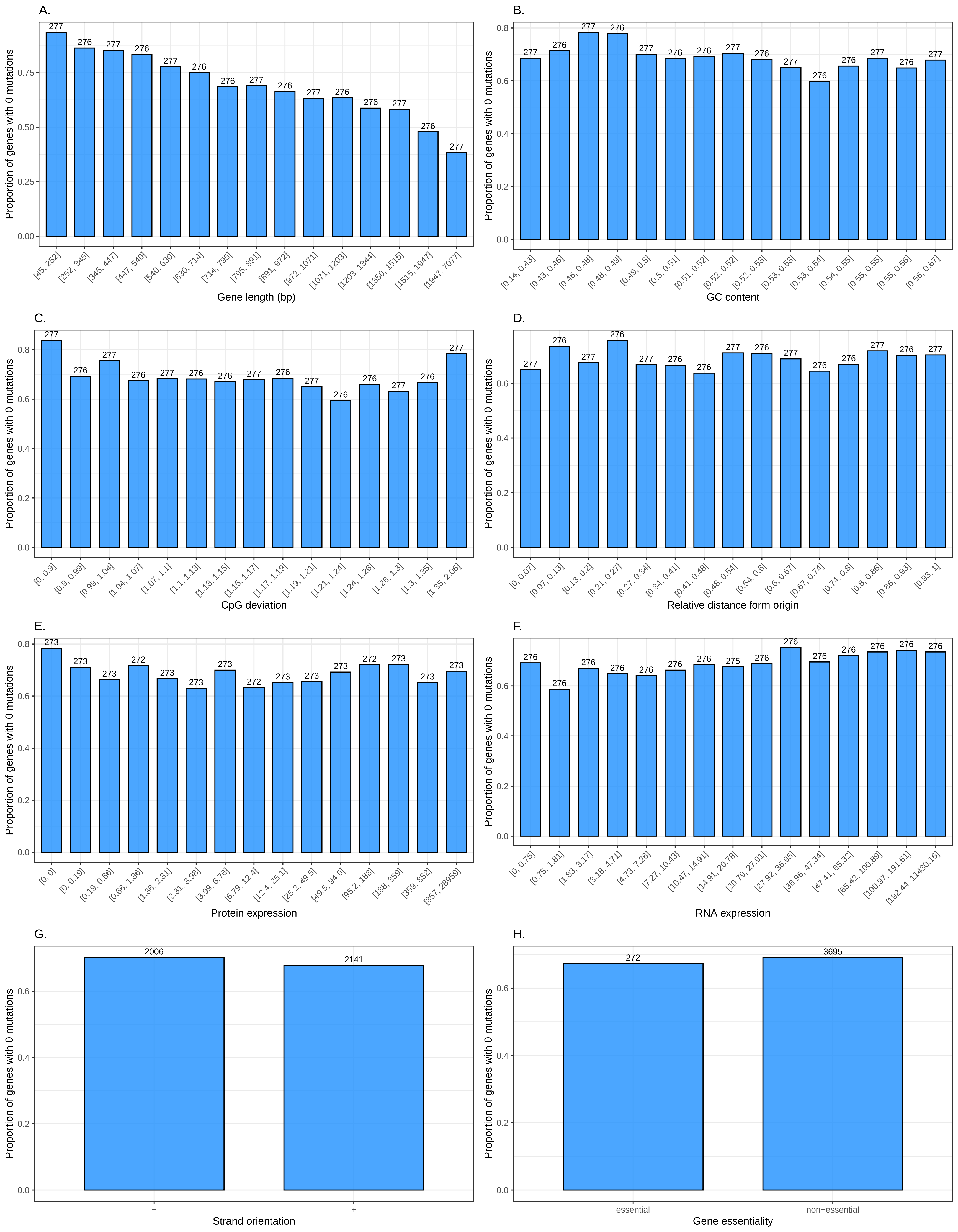
A.
Proportion of genes with 0 mutations
0.00
0.25
0.50
0.75
277
276
277
276
277
276
276
277
276
277
276
276
277
276
277
[45, 252]
[252, 345]
[345, 447]
[447, 540]
[540, 630]
[630, 714]
[714, 795]
[795, 891]
[891, 972]
[972, 1071]
[1071, 1203]
[1203, 1344]
[1350, 1515]
[1515, 1947]
[1947, 7077]
Gene length (bp)
B.
Proportion of genes with 0 mutations
0.0
0.2
0.4
0.6
0.8
277
276
277
276
277
276
276
277
276
277
276
276
277
276
277
[0.14, 0.43]
[0.43, 0.46]
[0.46, 0.48]
[0.48, 0.49]
[0.49, 0.5]
[0.5, 0.51]
[0.51, 0.52]
[0.52, 0.52]
[0.52, 0.53]
[0.53, 0.53]
[0.53, 0.54]
[0.54, 0.55]
[0.55, 0.55]
[0.55, 0.56]
[0.56, 0.67]
GC content
C.
Proportion of genes with 0 mutations
0.0
0.2
0.4
0.6
0.8
277
276
277
276
277
276
276
277
276
277
276
276
277
276
277
[0, 0.9]
[0.9, 0.99]
[0.99, 1.04]
[1.04, 1.07]
[1.07, 1.1]
[1.1, 1.13]
[1.13, 1.15]
[1.15, 1.17]
[1.17, 1.19]
[1.19, 1.21]
[1.21, 1.24]
[1.24, 1.26]
[1.26, 1.3]
[1.3, 1.35]
[1.35, 2.06]
CpG deviation
D.
Proportion of genes with 0 mutations
0.0
0.2
0.4
0.6
277
276
277
276
277
276
276
277
276
277
276
276
277
276
277
[0, 0.07]
[0.07, 0.13]
[0.13, 0.2]
[0.21, 0.27]
[0.27, 0.34]
[0.34, 0.41]
[0.41, 0.48]
[0.48, 0.54]
[0.54, 0.6]
[0.6, 0.67]
[0.67, 0.74]
[0.74, 0.8]
[0.8, 0.86]
[0.86, 0.93]
[0.93, 1]
Relative distance form origin
E.
Proportion of genes with 0 mutations
0.0
0.2
0.4
0.6
0.8
273
273
273
272
273
273
273
272
273
273
273
272
273
273
273
[0, 0]
[0, 0.19]
[0.19, 0.66]
[0.66, 1.36]
[1.36, 2.31]
[2.31, 3.98]
[3.99, 6.76]
[6.79, 12.4]
[12.4, 25.1]
[25.2, 49.5]
[49.5, 94.6]
[95.2, 188]
[188, 359]
[359, 852]
[857, 28959]
Protein expression
F.
Proportion of genes with 0 mutations
0.0
0.2
0.4
0.6
276
276
276
276
276
276
276
275
276
276
276
276
276
276
276
[0, 0.75]
[0.75, 1.81]
[1.83, 3.17]
[3.18, 4.71]
[4.73, 7.26]
[7.27, 10.43]
[10.47, 14.91]
[14.91, 20.78]
[20.79, 27.91]
[27.92, 36.95]
[36.96, 47.34]
[47.41, 65.32]
[65.42, 100.89]
[100.97, 191.61]
[192.44, 11430.16]
RNA expression
G.
Proportion of genes with 0 mutations
0.0
0.2
0.4
0.6
2006
2141
−
+
Strand orientation
H.
Proportion of genes with 0 mutations
0.0
0.2
0.4
0.6
272
3695
essential
non−essential
Gene essentiality
