# Supplementary material for: The importance of genes does not predict their mutation rate
Source: PLoS Biol. 2026 Nov 12;24(11):e3004089. doi: 10.1371/journal.pbio.3004089 (PMC13759209; doi:10.1371/journal.pbio.3004089)
Supplement: Supplementary file 7 [file pbio.3004089.s007.pdf]

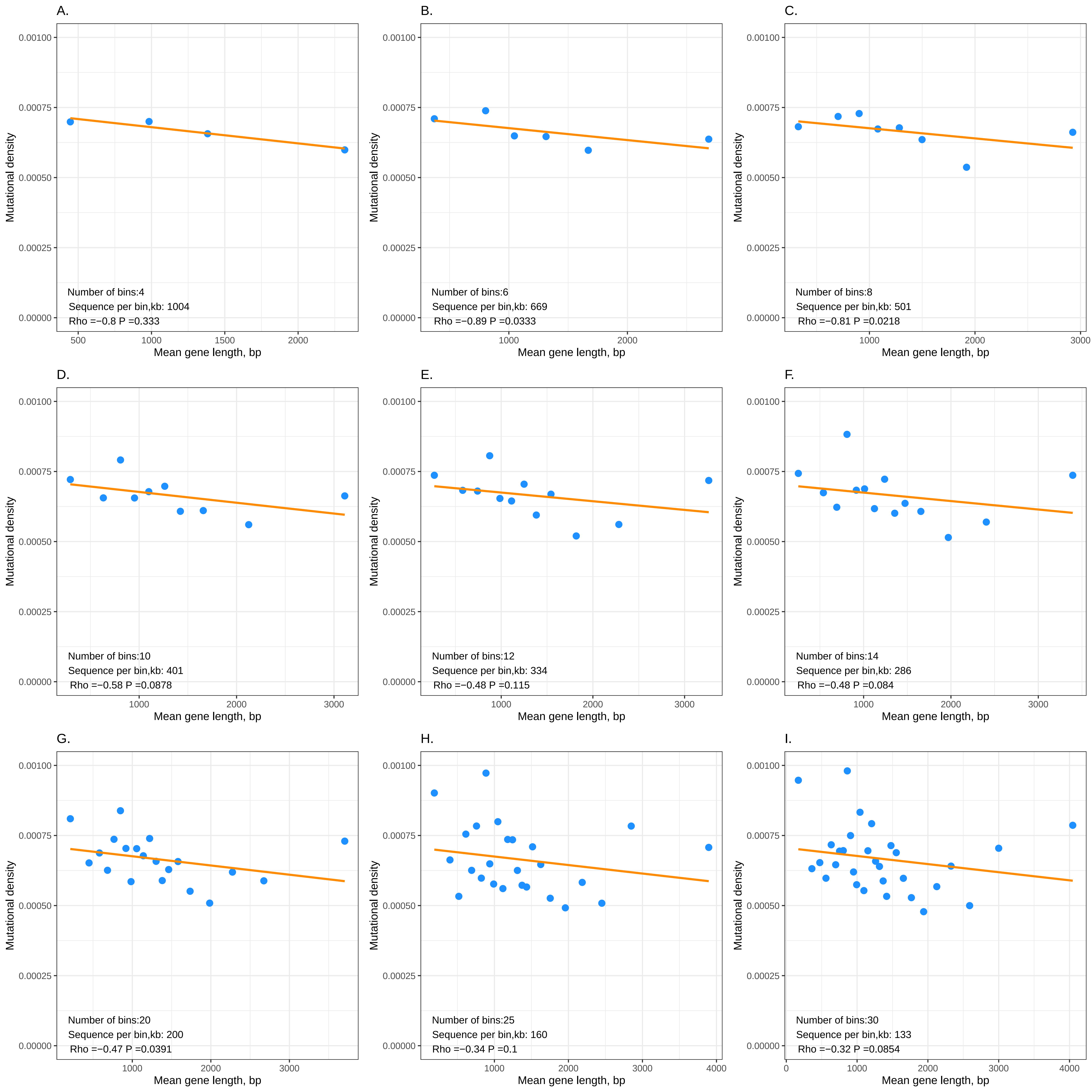
A.
Number of bins:4
Sequence per bin,kb: 1004
Rho =−0.8 P =0.333
B.
Number of bins:6
Sequence per bin,kb: 669
Rho =−0.89 P =0.0333
C.
Number of bins:8
Sequence per bin,kb: 501
Rho =−0.81 P =0.0218
D.
Number of bins:10
Sequence per bin,kb: 401
Rho =−0.58 P =0.0878
E.
Number of bins:12
Sequence per bin,kb: 334
Rho =−0.48 P =0.115
F.
Number of bins:14
Sequence per bin,kb: 286
Rho =−0.48 P =0.084
G.
Number of bins:20
Sequence per bin,kb: 200
Rho =−0.47 P =0.0391
H.
Number of bins:25
Sequence per bin,kb: 160
Rho =−0.34 P =0.1
I.
Number of bins:30
Sequence per bin,kb: 133
Rho =−0.32 P =0.0854
Mutational density
Mean gene length, bp
