# Supplementary material for: The importance of genes does not predict their mutation rate
Source: PLoS Biol. 2026 Nov 12;24(11):e3004089. doi: 10.1371/journal.pbio.3004089 (PMC13759209; doi:10.1371/journal.pbio.3004089)
Supplement: Supplementary file 8 [file pbio.3004089.s008.pdf]

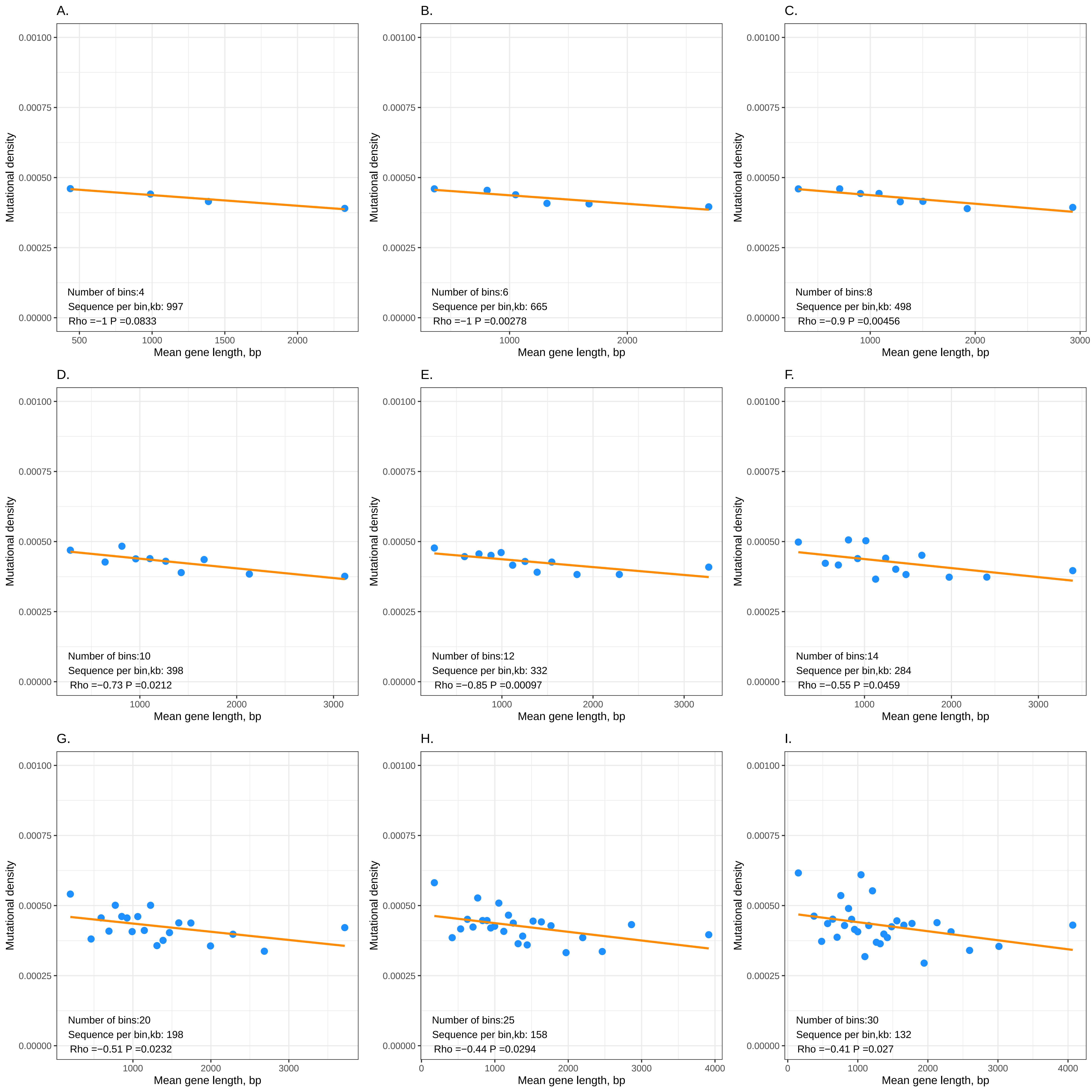
A.
Number of bins:4
Sequence per bin,kb: 997
Rho =−1 P =0.0833
B.
Number of bins:6
Sequence per bin,kb: 665
Rho =−1 P =0.00278
C.
Number of bins:8
Sequence per bin,kb: 498
Rho =−0.9 P =0.00456
D.
Number of bins:10
Sequence per bin,kb: 398
Rho =−0.73 P =0.0212
E.
Number of bins:12
Sequence per bin,kb: 332
Rho =−0.85 P =0.00097
F.
Number of bins:14
Sequence per bin,kb: 284
Rho =−0.55 P =0.0459
G.
Number of bins:20
Sequence per bin,kb: 198
Rho =−0.51 P =0.0232
H.
Number of bins:25
Sequence per bin,kb: 158
Rho =−0.44 P =0.0294
I.
Number of bins:30
Sequence per bin,kb: 132
Rho =−0.41 P =0.027
Mutational density
Mean gene length, bp
0.00000
0.00025
0.00050
0.00075
0.00100
