# Supplementary material for: The importance of genes does not predict their mutation rate
Source: PLoS Biol. 2026 Nov 12;24(11):e3004089. doi: 10.1371/journal.pbio.3004089 (PMC13759209; doi:10.1371/journal.pbio.3004089)
Supplement: Supplementary file 9 [file pbio.3004089.s009.pdf]

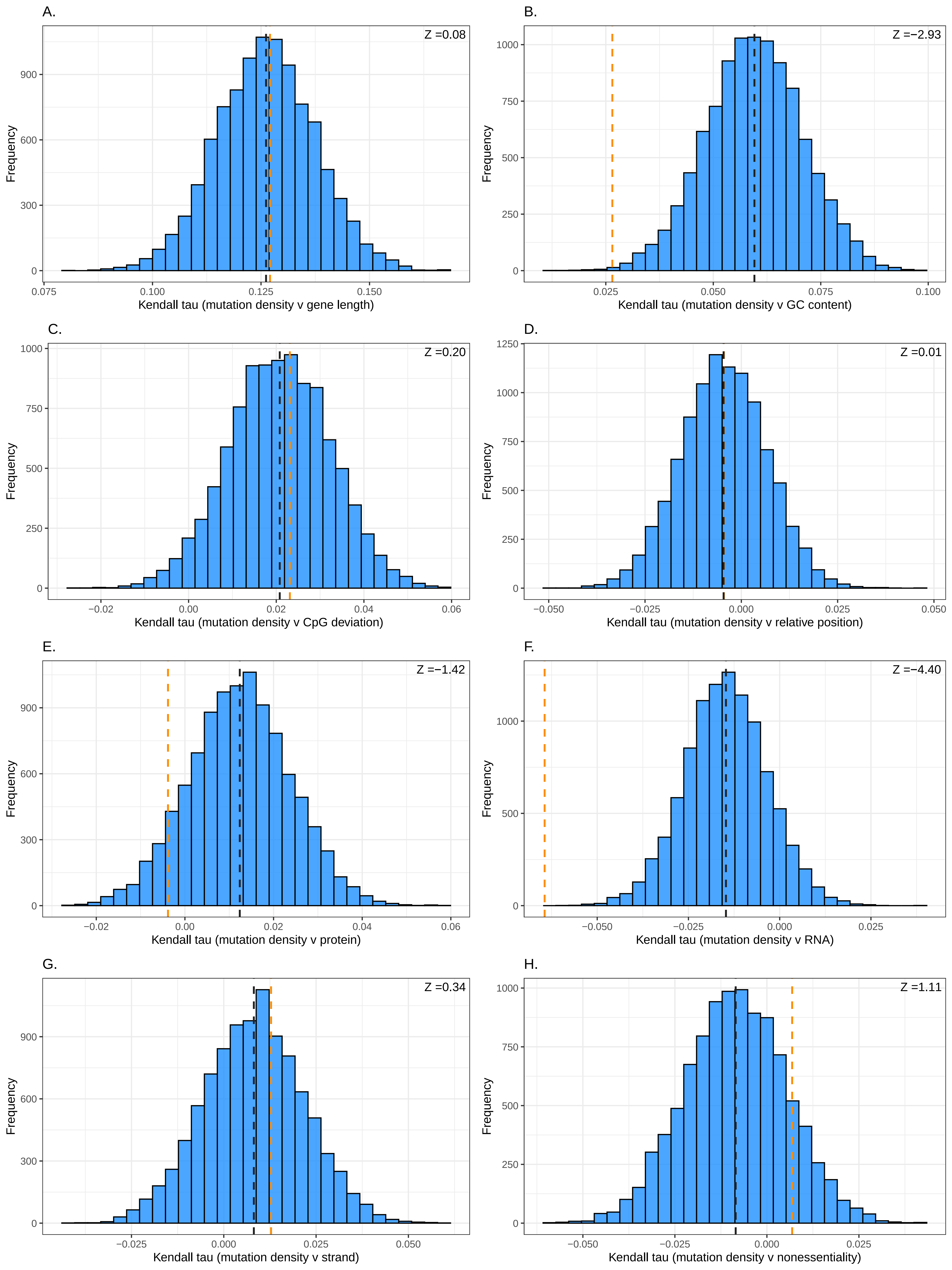
A.
Z =0.08
Frequency
Kendall tau (mutation density v gene length)
B.
Z =−2.93
Frequency
Kendall tau (mutation density v GC content)
C.
Z =0.20
Frequency
Kendall tau (mutation density v CpG deviation)
D.
Z =0.01
Frequency
Kendall tau (mutation density v relative position)
E.
Z =−1.42
Frequency
Kendall tau (mutation density v protein)
F.
Z =−4.40
Frequency
Kendall tau (mutation density v RNA)
G.
Z =0.34
Frequency
Kendall tau (mutation density v strand)
H.
Z =1.11
Frequency
Kendall tau (mutation density v nonessentiality)
