# Supplementary material for: The importance of genes does not predict their mutation rate
Source: PLoS Biol. 2026 Nov 12;24(11):e3004089. doi: 10.1371/journal.pbio.3004089 (PMC13759209; doi:10.1371/journal.pbio.3004089)
Supplement: Supplementary file 10 [file pbio.3004089.s010.pdf]

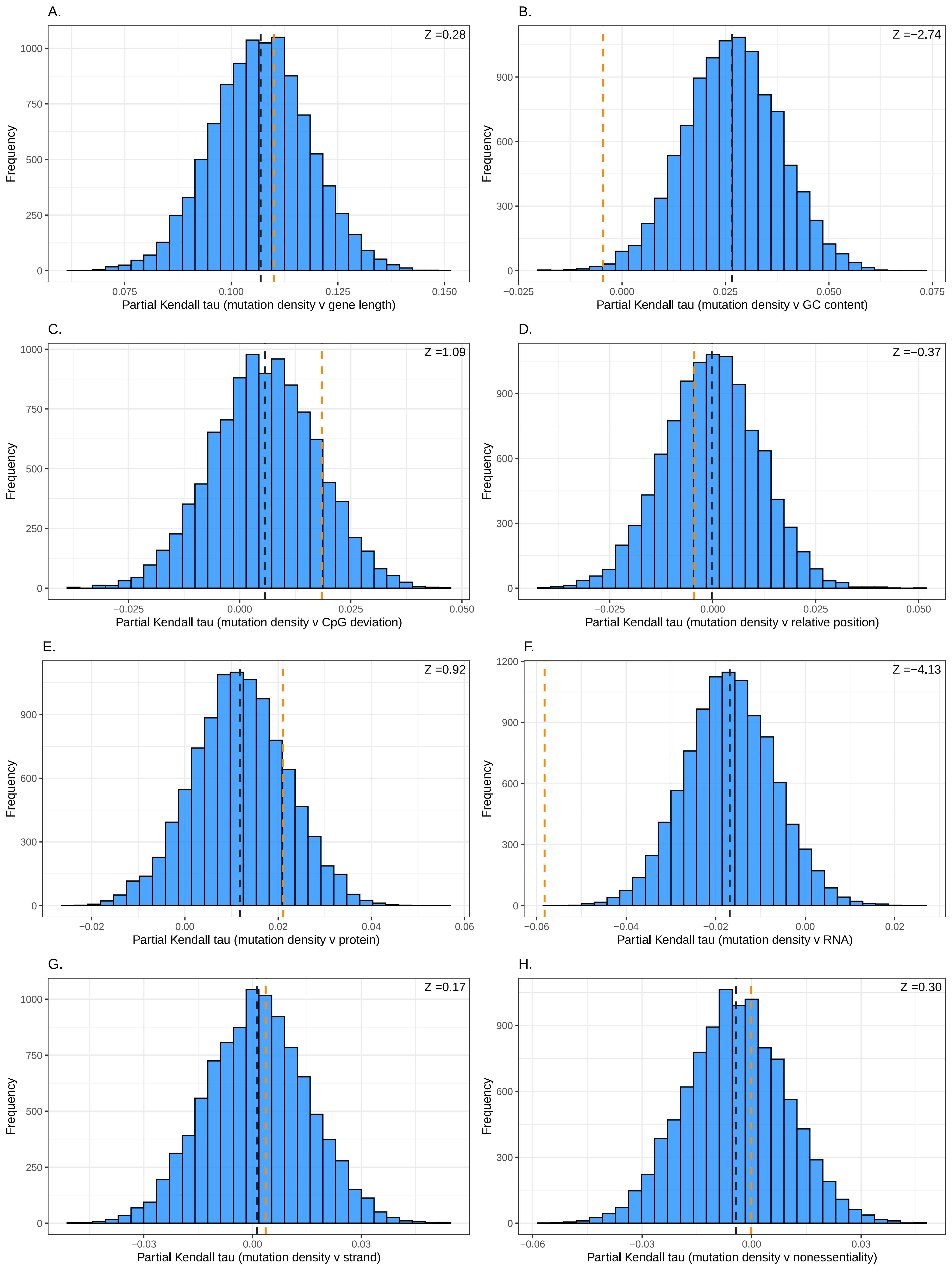
A.
Z =0.28
Frequency
0
250
500
750
1000
0.075
0.100
0.125
0.150
Partial Kendall tau (mutation density v gene length)
B.
Z =−2.74
Frequency
0
300
600
900
−0.025
0.000
0.025
0.050
0.075
Partial Kendall tau (mutation density v GC content)
C.
Z =1.09
Frequency
0
250
500
750
1000
−0.025
0.000
0.025
0.050
Partial Kendall tau (mutation density v CpG deviation)
D.
Z =−0.37
Frequency
0
300
600
900
−0.025
0.000
0.025
0.050
Partial Kendall tau (mutation density v relative position)
E.
Z =0.92
Frequency
0
300
600
900
−0.02
0.00
0.02
0.04
0.06
Partial Kendall tau (mutation density v protein)
F.
Z =−4.13
Frequency
0
300
600
900
1200
−0.06
−0.04
−0.02
0.00
0.02
Partial Kendall tau (mutation density v RNA)
G.
Z =0.17
Frequency
0
250
500
750
1000
−0.03
0.00
0.03
Partial Kendall tau (mutation density v strand)
H.
Z =0.30
Frequency
0
300
600
900
−0.06
−0.03
0.00
0.03
Partial Kendall tau (mutation density v nonessentiality)
