# Supplementary material for: The importance of genes does not predict their mutation rate
Source: PLoS Biol. 2026 Nov 12;24(11):e3004089. doi: 10.1371/journal.pbio.3004089 (PMC13759209; doi:10.1371/journal.pbio.3004089)
Supplement: Supplementary file 11 [file pbio.3004089.s011.pdf]

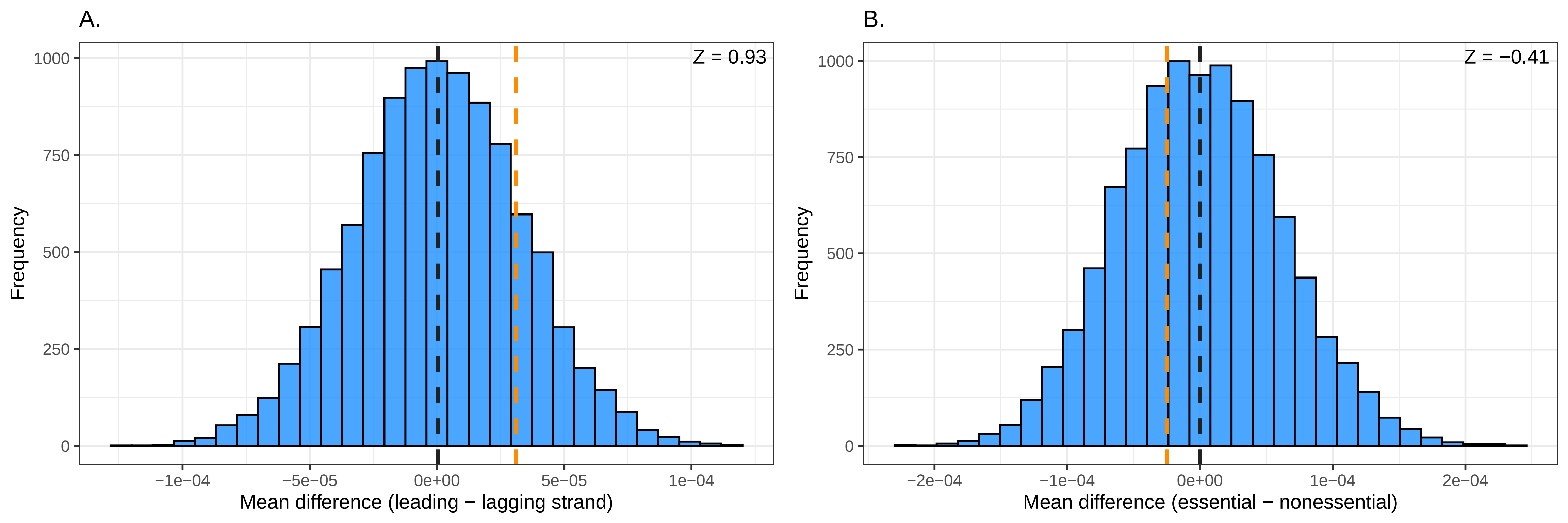

A.
Z = 0.93
Frequency
1000
750
500
250
0
−1e−04
−5e−05
0e+00
5e−05
1e−04
Mean difference (leading − lagging strand)
B.
Z = −0.41
Frequency
1000
750
500
250
0
−2e−04
−1e−04
0e+00
1e−04
2e−04
Mean difference (essential − nonessential)
