# Supplementary material for: The importance of genes does not predict their mutation rate
Source: PLoS Biol. 2026 Nov 12;24(11):e3004089. doi: 10.1371/journal.pbio.3004089 (PMC13759209; doi:10.1371/journal.pbio.3004089)
Supplement: Supplementary file 12 [file pbio.3004089.s012.pdf]

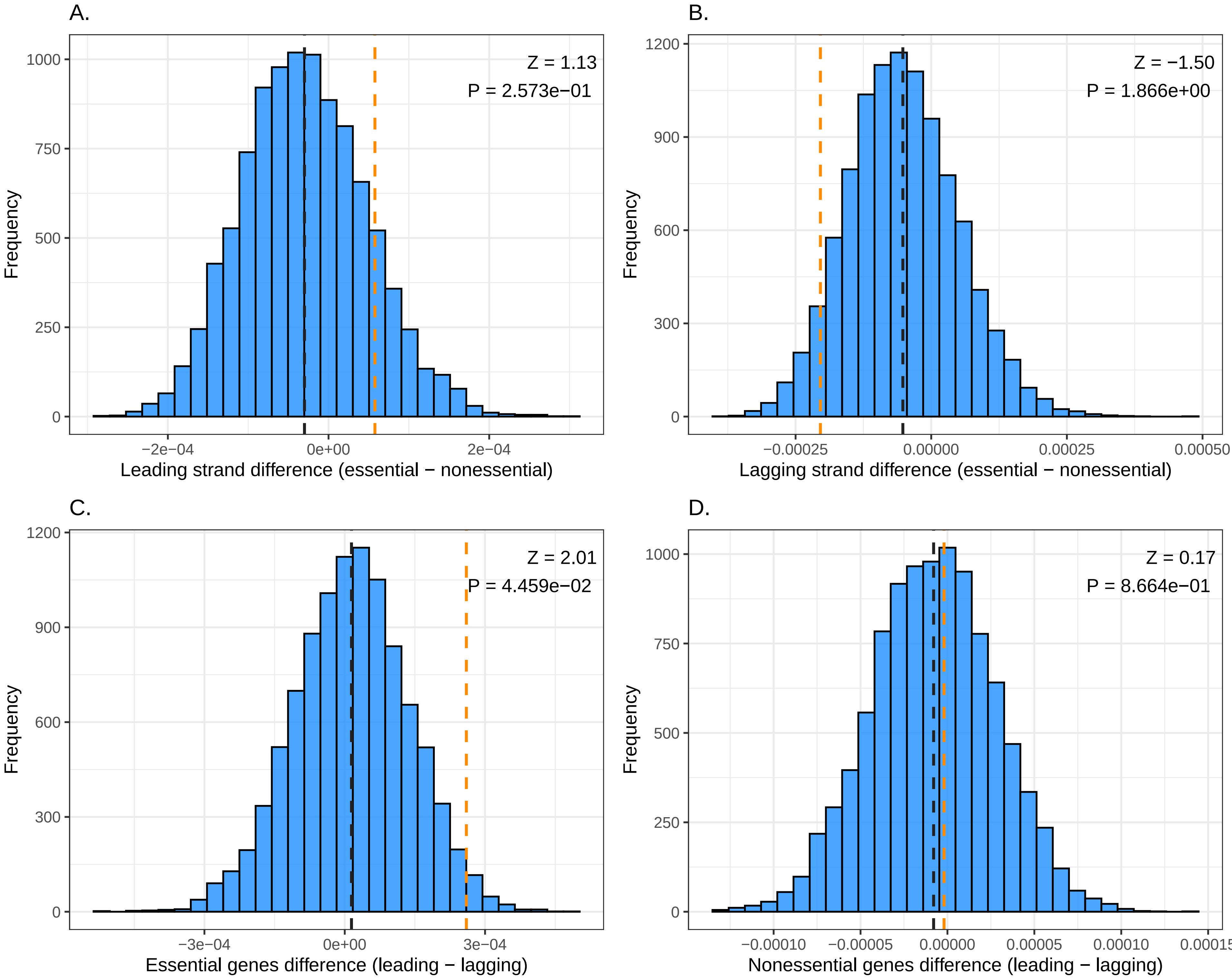
A.
Z = 1.13
P = 2.573e−01
Frequency
1000
750
500
250
0
−2e−04
0e+00
2e−04
Leading strand difference (essential − nonessential)
B.
Z = −1.50
P = 1.866e+00
Frequency
1200
900
600
300
0
−0.00025
0.00000
0.00025
0.00050
Lagging strand difference (essential − nonessential)
C.
Z = 2.01
P = 4.459e−02
Frequency
1200
900
600
300
0
−3e−04
0e+00
3e−04
Essential genes difference (leading − lagging)
D.
Z = 0.17
P = 8.664e−01
Frequency
1000
750
500
250
0
−0.00010
−0.00005
0.00000
0.00005
0.00010
0.00015
Nonessential genes difference (leading − lagging)
