# Supplementary material for: The importance of genes does not predict their mutation rate
Source: PLoS Biol. 2026 Nov 12;24(11):e3004089. doi: 10.1371/journal.pbio.3004089 (PMC13759209; doi:10.1371/journal.pbio.3004089)
Supplement: Supplementary file 13 [file pbio.3004089.s013.pdf]

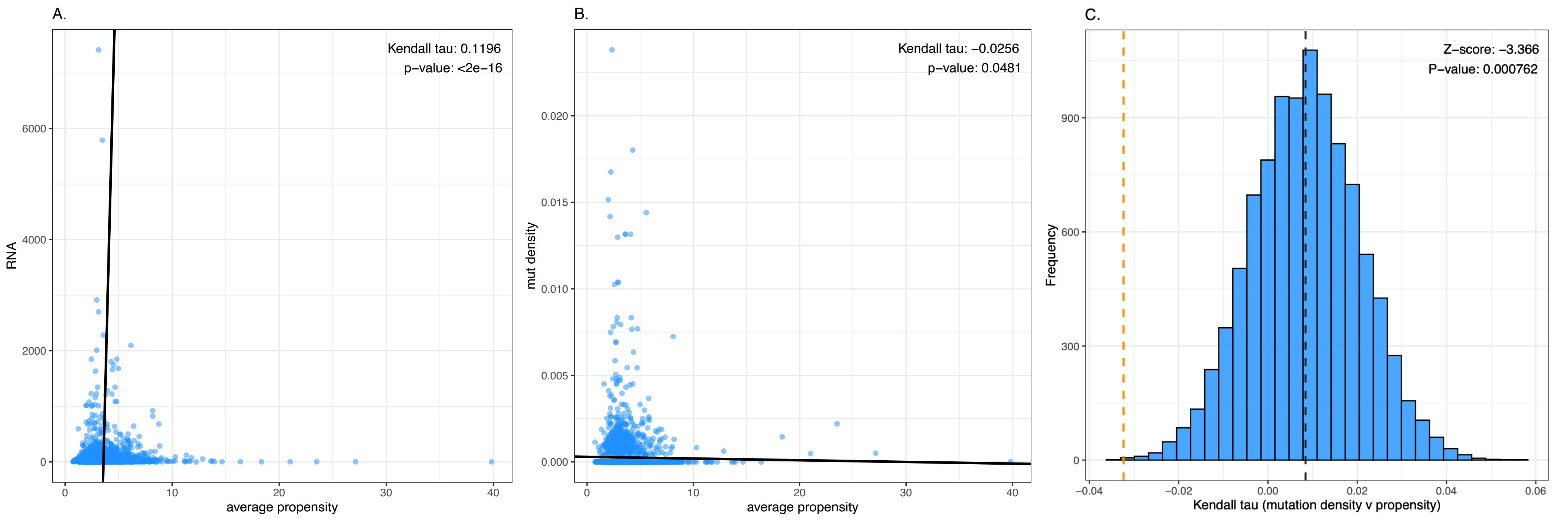
A.
Kendall tau: 0.1196
p−value: <2e−16
RNA
0
2000
4000
6000
0
10
20
30
40
average propensity
B.
Kendall tau: −0.0256
p−value: 0.0481
mut density
0.000
0.005
0.010
0.015
0.020
0
10
20
30
40
average propensity
C.
Z−score: −3.366
P−value: 0.000762
Frequency
0
300
600
900
−0.04
−0.02
0.00
0.02
0.04
0.06
Kendall tau (mutation density v propensity)
