# Supplementary material for: The importance of genes does not predict their mutation rate
Source: PLoS Biol. 2026 Nov 12;24(11):e3004089. doi: 10.1371/journal.pbio.3004089 (PMC13759209; doi:10.1371/journal.pbio.3004089)
Supplement: Supplementary file 14 [file pbio.3004089.s014.pdf]

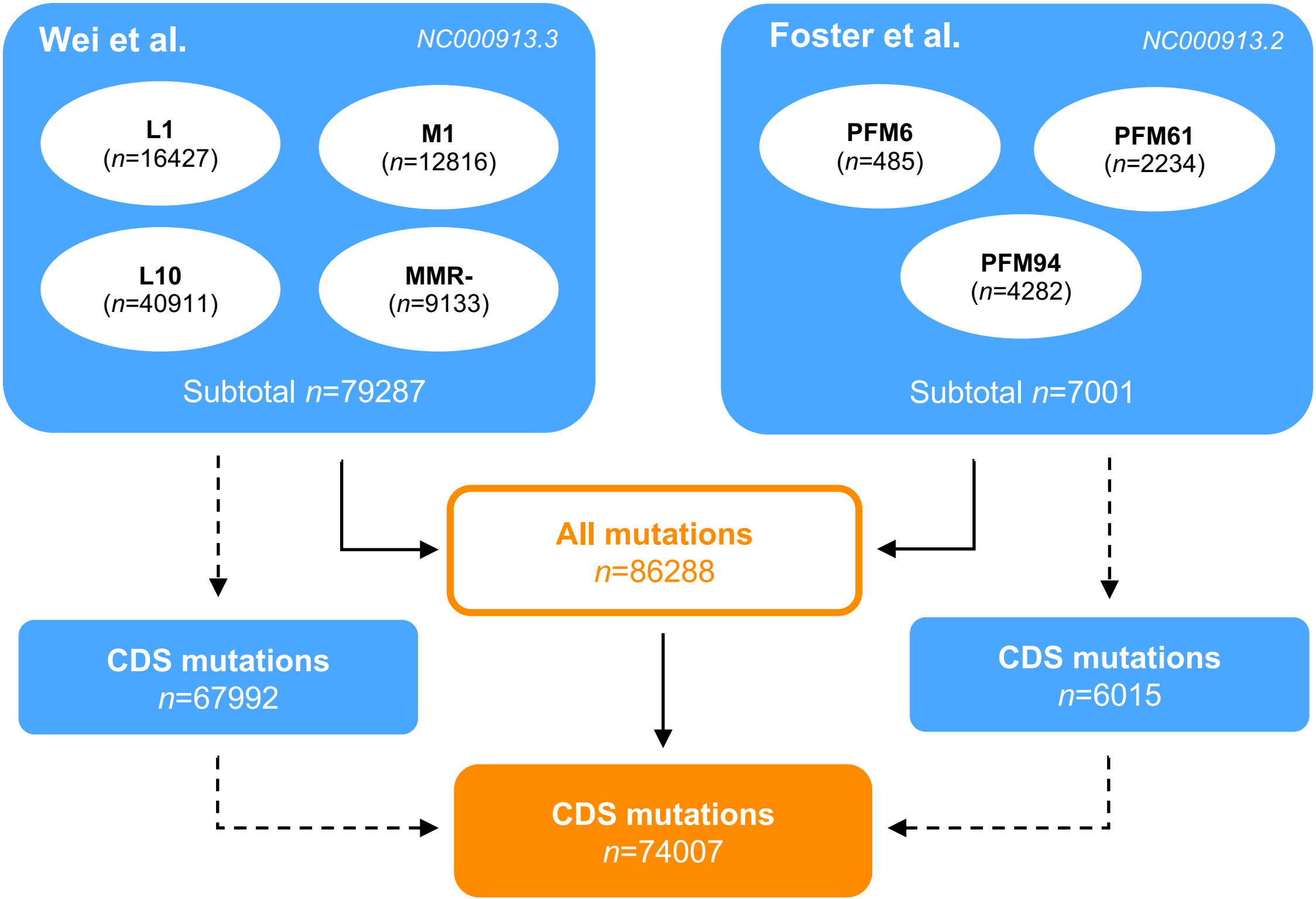
Wei et al.
NC000913.3
L1
(n=16427)
M1
(n=12816)
L10
(n=40911)
MMR-
(n=9133)
Subtotal n=79287
Foster et al.
NC000913.2
PFM6
(n=485)
PFM61
(n=2234)
PFM94
(n=4282)
Subtotal n=7001
All mutations
n=86288
CDS mutations
n=67992
CDS mutations
n=6015
CDS mutations
n=74007
