# Supplementary material for: The importance of genes does not predict their mutation rate
Source: PLoS Biol. 2026 Nov 12;24(11):e3004089. doi: 10.1371/journal.pbio.3004089 (PMC13759209; doi:10.1371/journal.pbio.3004089)
Supplement: Supplementary file 15 [file pbio.3004089.s015.pdf]

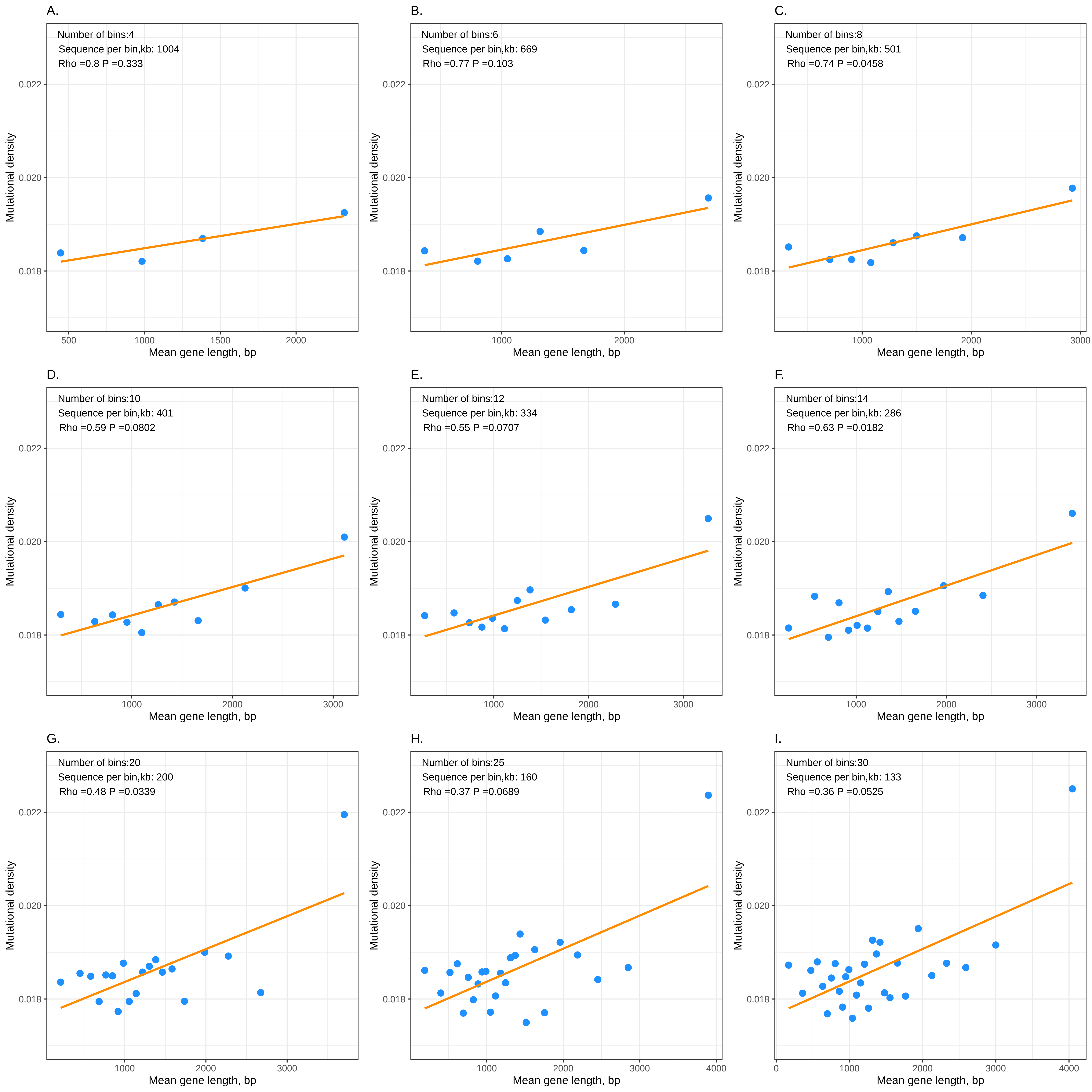
A.
Number of bins:4
Sequence per bin,kb: 1004
Rho =0.8 P =0.333
B.
Number of bins:6
Sequence per bin,kb: 669
Rho =0.77 P =0.103
C.
Number of bins:8
Sequence per bin,kb: 501
Rho =0.74 P =0.0458
D.
Number of bins:10
Sequence per bin,kb: 401
Rho =0.59 P =0.0802
E.
Number of bins:12
Sequence per bin,kb: 334
Rho =0.55 P =0.0707
F.
Number of bins:14
Sequence per bin,kb: 286
Rho =0.63 P =0.0182
G.
Number of bins:20
Sequence per bin,kb: 200
Rho =0.48 P =0.0339
H.
Number of bins:25
Sequence per bin,kb: 160
Rho =0.37 P =0.0689
I.
Number of bins:30
Sequence per bin,kb: 133
Rho =0.36 P =0.0525
Mutational density
Mean gene length, bp
