# Supplementary material for: The importance of genes does not predict their mutation rate
Source: PLoS Biol. 2026 Nov 12;24(11):e3004089. doi: 10.1371/journal.pbio.3004089 (PMC13759209; doi:10.1371/journal.pbio.3004089)
Supplement: Supplementary file 16 [file pbio.3004089.s016.pdf]

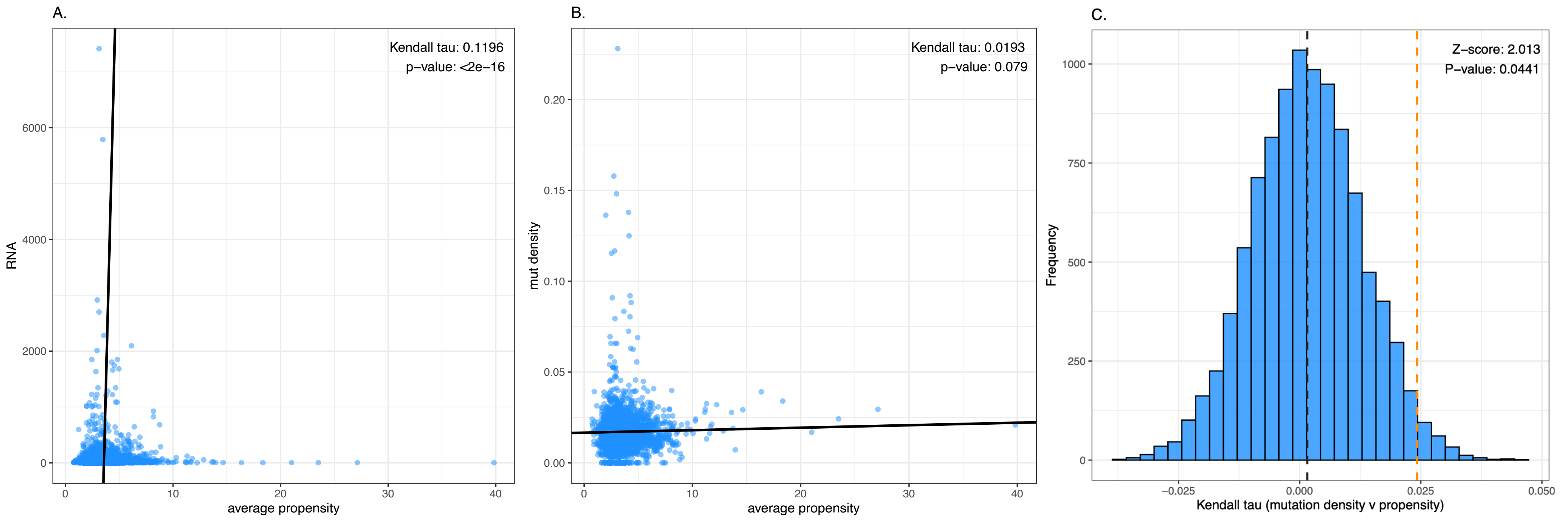
A.
Kendall tau: 0.1196
p−value: <2e−16
RNA
6000
4000
2000
0
0
10
20
30
40
average propensity
B.
Kendall tau: 0.0193
p−value: 0.079
mut density
0.20
0.15
0.10
0.05
0.00
0
10
20
30
40
average propensity
C.
Z−score: 2.013
P−value: 0.0441
Frequency
1000
750
500
250
0
−0.025
0.000
0.025
0.050
Kendall tau (mutation density v propensity)
