# Supplementary material for: The importance of genes does not predict their mutation rate
Source: PLoS Biol. 2026 Nov 12;24(11):e3004089. doi: 10.1371/journal.pbio.3004089 (PMC13759209; doi:10.1371/journal.pbio.3004089)
Supplement: Supplementary file 17 [file pbio.3004089.s017.pdf]

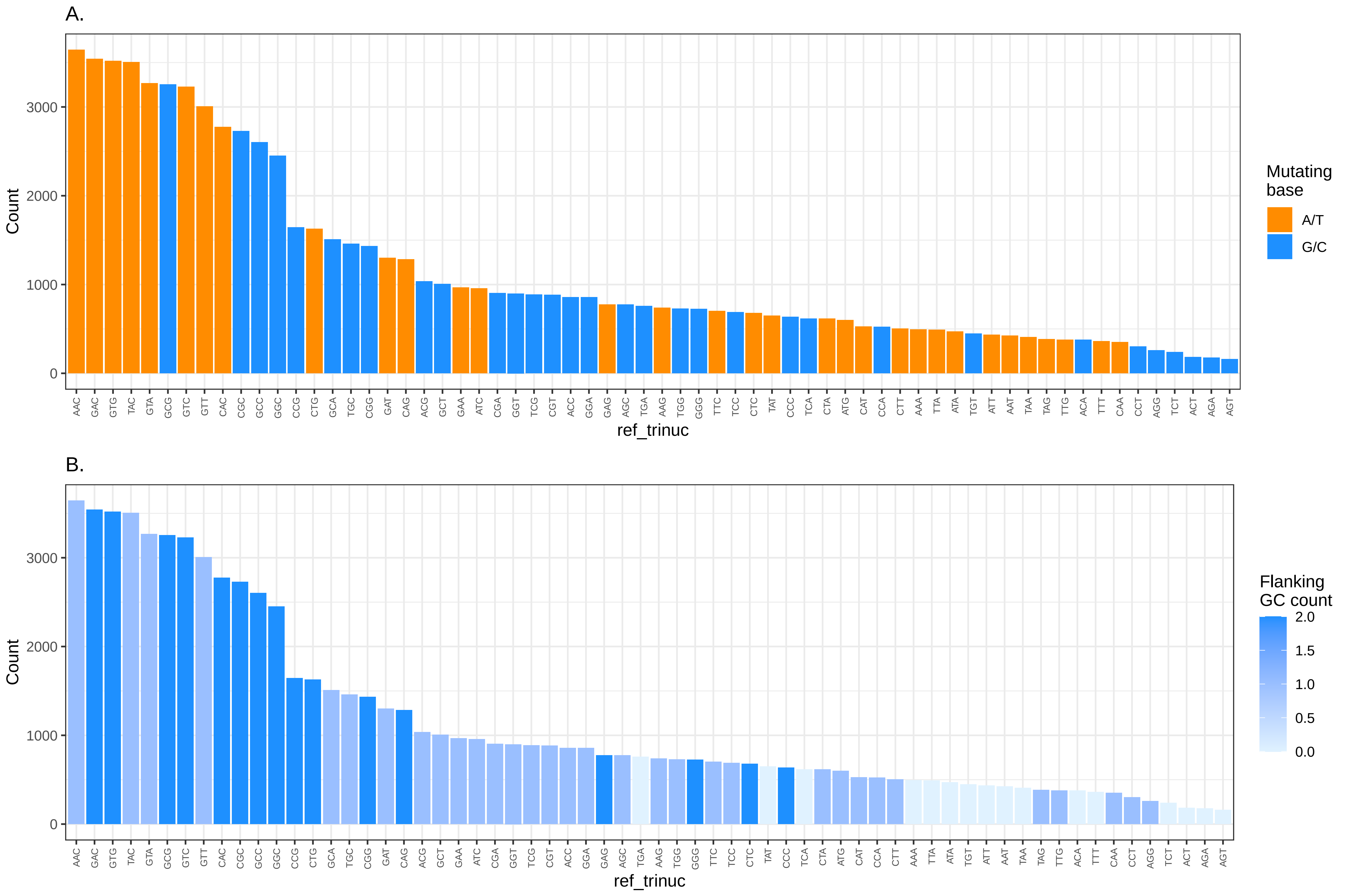
A.
Count
ref_trinuc
Mutating base
A/T
G/C
B.
Count
ref_trinuc
Flanking GC count
2.0
1.5
1.0
0.5
0.0
0
1000
2000
3000
AAC GAC GTG TAC GTA GCG GTC GTT CAC CGC GCC GGC CCG CTG GCA TGC CGG GAT CAG ACG GCT GAA ATC CGA GGT TCG CGT ACC GGA GAG AGC TGA AAG TGG GGG TTC TCC CTC TAT CCC TCA CTA ATG CAT CCA CTT AAA TTA ATA TGT ATT AAT TAA TAG TTG ACA TTT CAA CCT AGG TCT ACT AGA AGT
