# Supplementary material for: The importance of genes does not predict their mutation rate
Source: PLoS Biol. 2026 Nov 12;24(11):e3004089. doi: 10.1371/journal.pbio.3004089 (PMC13759209; doi:10.1371/journal.pbio.3004089)
Supplement: Supplementary file 18 [file pbio.3004089.s018.pdf]

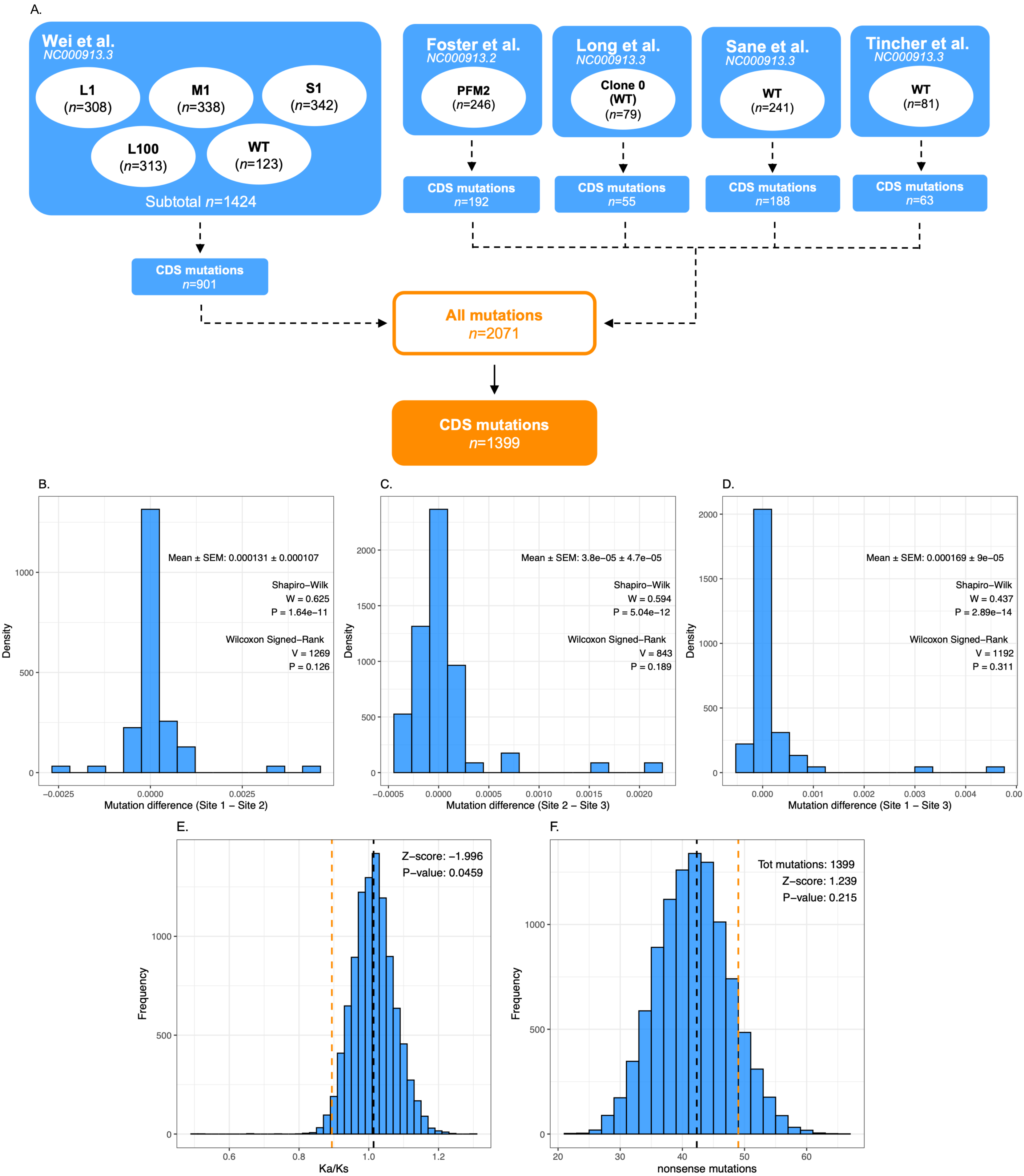
A.
Wei et al.
NC000913.3
L1 (n=308)
M1 (n=338)
S1 (n=342)
L100 (n=313)
WT (n=123)
Subtotal n=1424
CDS mutations n=901
Foster et al.
NC000913.2
PFM2 (n=246)
CDS mutations n=192
Long et al.
NC000913.3
Clone 0 (WT) (n=79)
CDS mutations n=55
Sane et al.
NC000913.3
WT (n=241)
CDS mutations n=188
Tincher et al.
NC000913.3
WT (n=81)
CDS mutations n=63
All mutations n=2071
CDS mutations n=1399
B.
Mean ± SEM: 0.000131 ± 0.000107
Shapiro–Wilk
W = 0.625
P = 1.64e–11
Wilcoxon Signed–Rank
V = 1269
P = 0.126
Density
Mutation difference (Site 1 – Site 2)
C.
Mean ± SEM: 3.8e–05 ± 4.7e–05
Shapiro–Wilk
W = 0.594
P = 5.04e–12
Wilcoxon Signed–Rank
V = 843
P = 0.189
Density
Mutation difference (Site 2 – Site 3)
D.
Mean ± SEM: 0.000169 ± 9e–05
Shapiro–Wilk
W = 0.437
P = 2.89e–14
Wilcoxon Signed–Rank
V = 1192
P = 0.311
Density
Mutation difference (Site 1 – Site 3)
E.
Z–score: –1.996
P–value: 0.0459
Frequency
Ka/Ks
F.
Tot mutations: 1399
Z–score: 1.239
P–value: 0.215
Frequency
nonsense mutations
