# Supplementary material for: The importance of genes does not predict their mutation rate
Source: PLoS Biol. 2026 Nov 12;24(11):e3004089. doi: 10.1371/journal.pbio.3004089 (PMC13759209; doi:10.1371/journal.pbio.3004089)
Supplement: Supplementary file 20 [file pbio.3004089.s020.pdf]

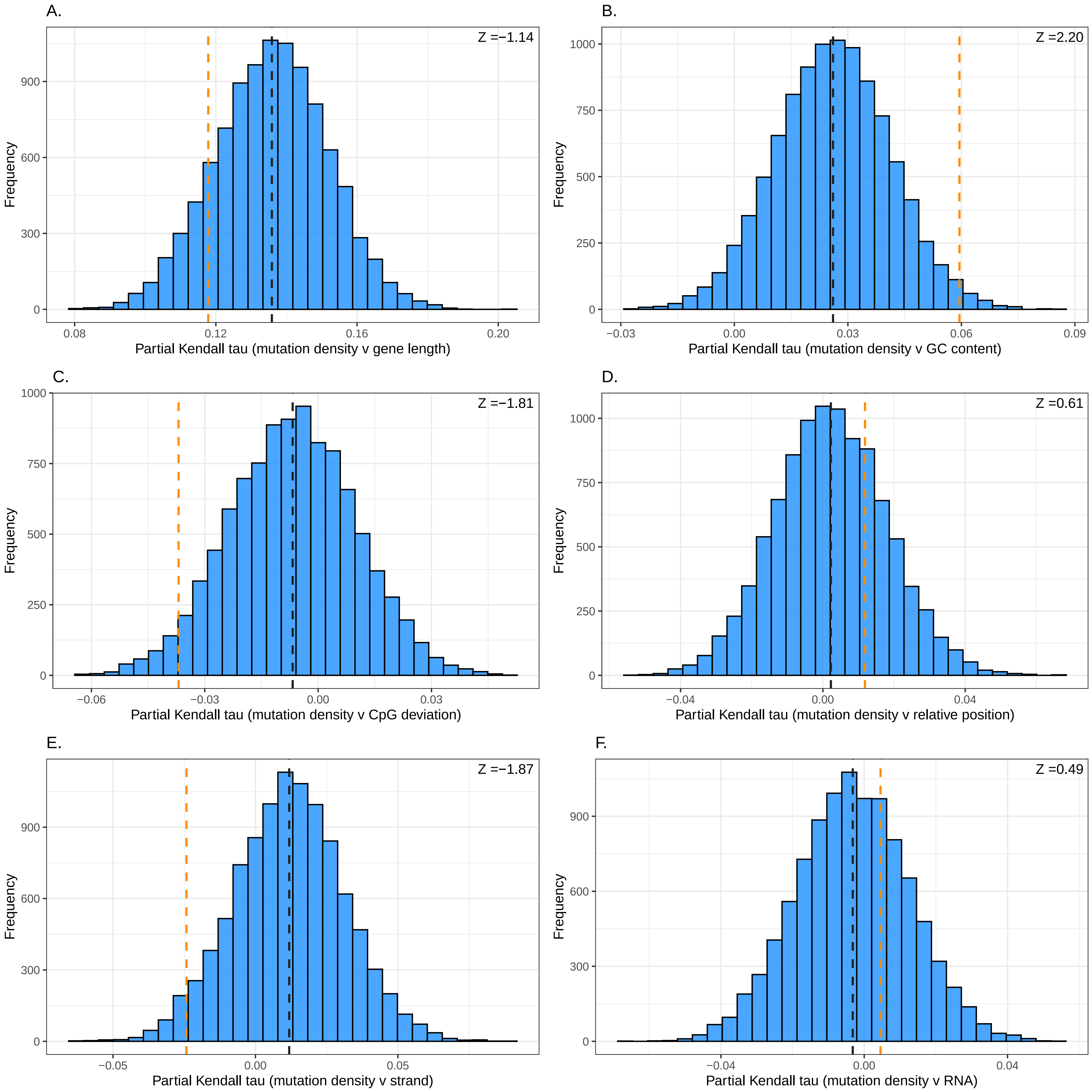

A.
Z =−1.14
Frequency
0
300
600
900
0.08
0.12
0.16
0.20
Partial Kendall tau (mutation density v gene length)
B.
Z =2.20
Frequency
0
250
500
750
1000
−0.03
0.00
0.03
0.06
0.09
Partial Kendall tau (mutation density v GC content)
C.
Z =−1.81
Frequency
0
250
500
750
1000
−0.06
−0.03
0.00
0.03
Partial Kendall tau (mutation density v CpG deviation)
D.
Z =0.61
Frequency
0
250
500
750
1000
−0.04
0.00
0.04
Partial Kendall tau (mutation density v relative position)
E.
Z =−1.87
Frequency
0
300
600
900
−0.05
0.00
0.05
Partial Kendall tau (mutation density v strand)
F.
Z =0.49
Frequency
0
300
600
900
−0.04
0.00
0.04
Partial Kendall tau (mutation density v RNA)
