# Supplementary material for: The importance of genes does not predict their mutation rate
Source: PLoS Biol. 2026 Nov 12;24(11):e3004089. doi: 10.1371/journal.pbio.3004089 (PMC13759209; doi:10.1371/journal.pbio.3004089)
Supplement: Supplementary file 21 [file pbio.3004089.s021.pdf]

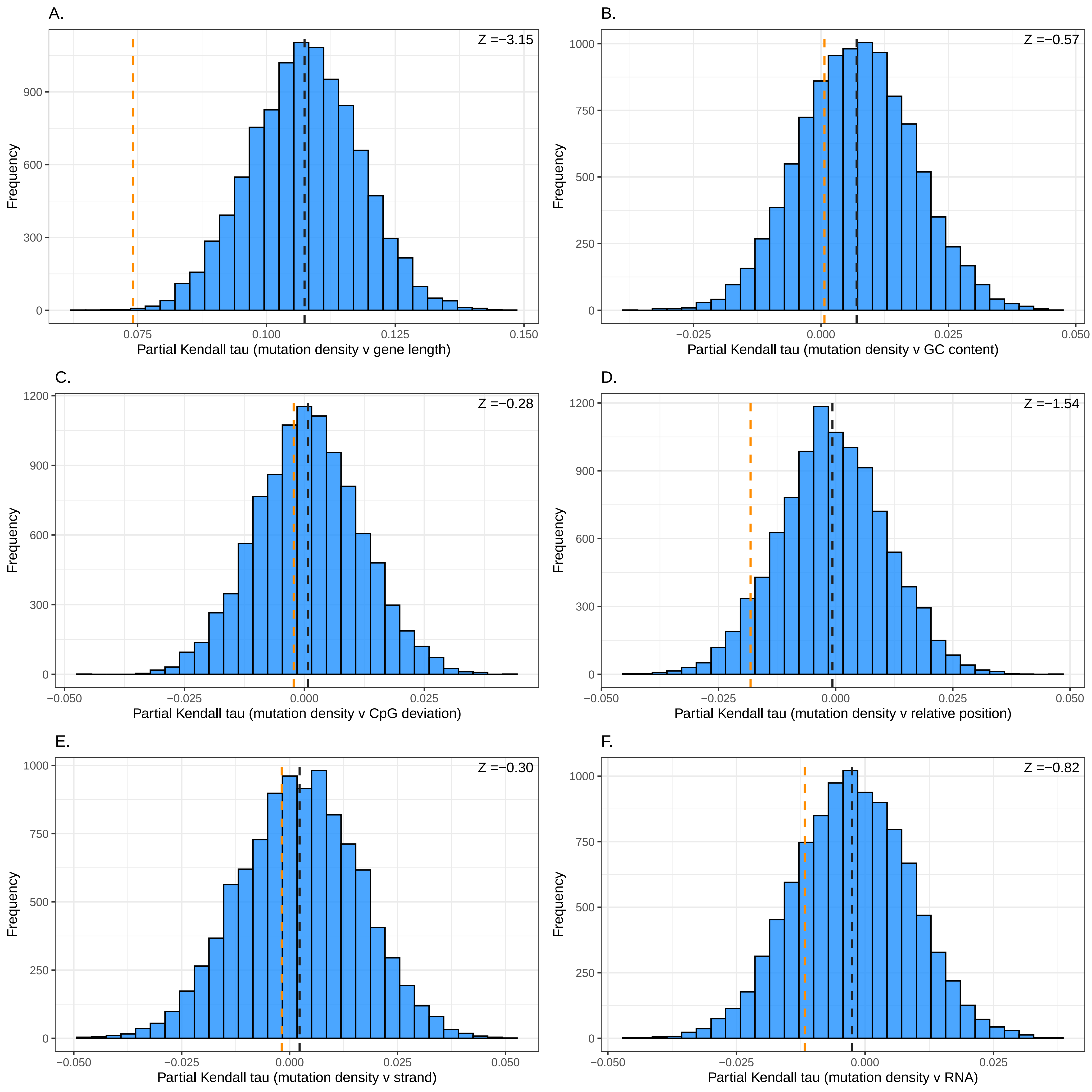

A.
Z =−3.15
Frequency
0
300
600
900
0.075
0.100
0.125
0.150
Partial Kendall tau (mutation density v gene length)
B.
Z =−0.57
Frequency
0
250
500
750
1000
−0.025
0.000
0.025
0.050
Partial Kendall tau (mutation density v GC content)
C.
Z =−0.28
Frequency
0
300
600
900
1200
−0.050
−0.025
0.000
0.025
Partial Kendall tau (mutation density v CpG deviation)
D.
Z =−1.54
Frequency
0
300
600
900
1200
−0.050
−0.025
0.000
0.025
0.050
Partial Kendall tau (mutation density v relative position)
E.
Z =−0.30
Frequency
0
250
500
750
1000
−0.050
−0.025
0.000
0.025
0.050
Partial Kendall tau (mutation density v strand)
F.
Z =−0.82
Frequency
0
250
500
750
1000
−0.050
−0.025
0.000
0.025
Partial Kendall tau (mutation density v RNA)
