# Supplementary material for: The importance of genes does not predict their mutation rate
Source: PLoS Biol. 2026 Nov 12;24(11):e3004089. doi: 10.1371/journal.pbio.3004089 (PMC13759209; doi:10.1371/journal.pbio.3004089)
Supplement: Supplementary file 22 [file pbio.3004089.s022.pdf]

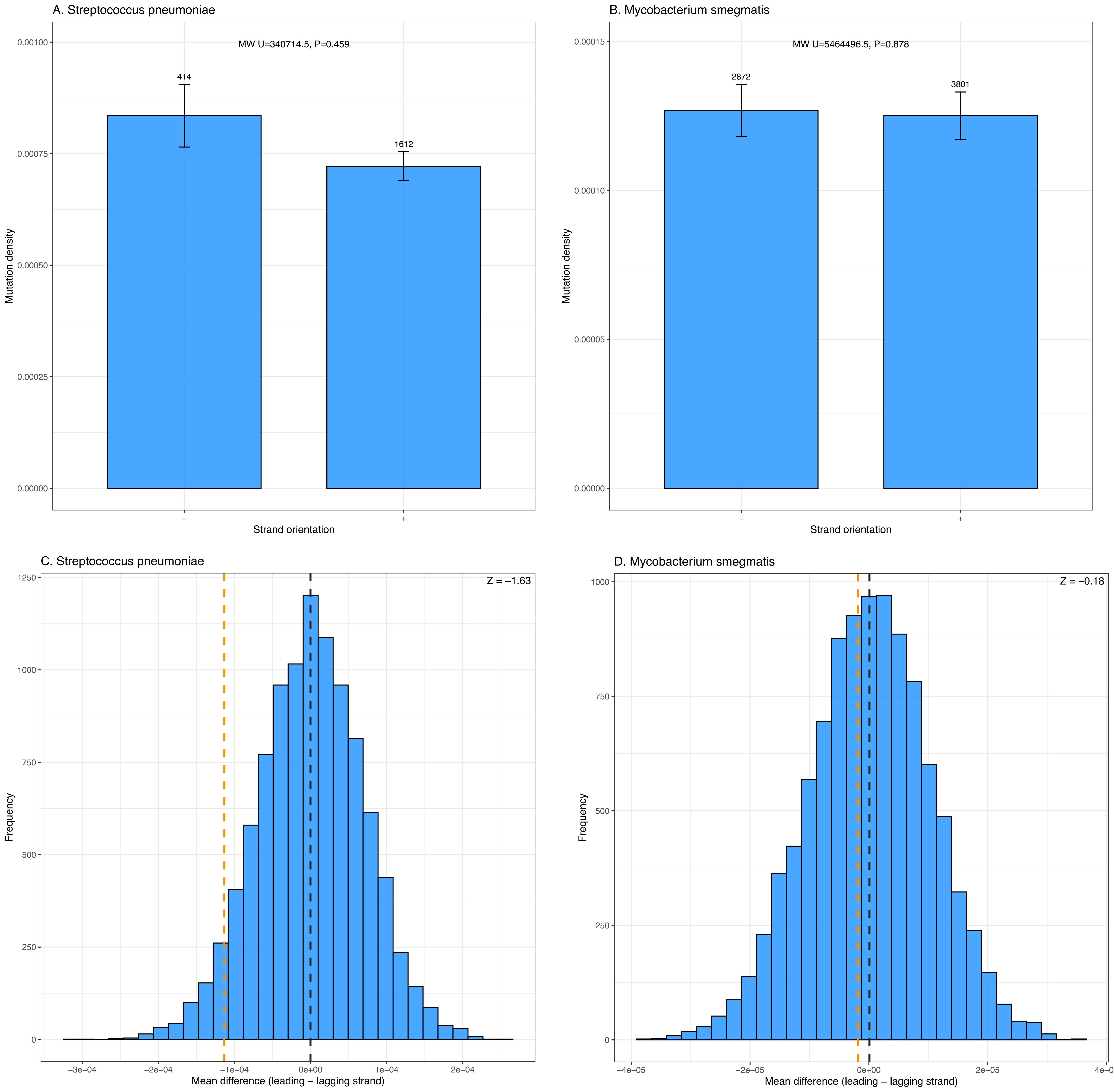

A. Streptococcus pneumoniae
MW U=340714.5, P=0.459
414
1612
Mutation density
0.00100
0.00075
0.00050
0.00025
0.00000
–
+
Strand orientation
B. Mycobacterium smegmatis
MW U=5464496.5, P=0.878
2872
3801
Mutation density
0.00015
0.00010
0.00005
0.00000
–
+
Strand orientation
C. Streptococcus pneumoniae
Z = –1.63
Frequency
1250
1000
750
500
250
0
–3e–04
–2e–04
–1e–04
0e+00
1e–04
2e–04
Mean difference (leading – lagging strand)
D. Mycobacterium smegmatis
Z = –0.18
Frequency
1000
750
500
250
0
–4e–05
–2e–05
0e+00
2e–05
4e–05
Mean difference (leading – lagging strand)
