# Supplementary material for: The importance of genes does not predict their mutation rate
Source: PLoS Biol. 2026 Nov 12;24(11):e3004089. doi: 10.1371/journal.pbio.3004089 (PMC13759209; doi:10.1371/journal.pbio.3004089)
Supplement: Supplementary file 23 [file pbio.3004089.s023.pdf]

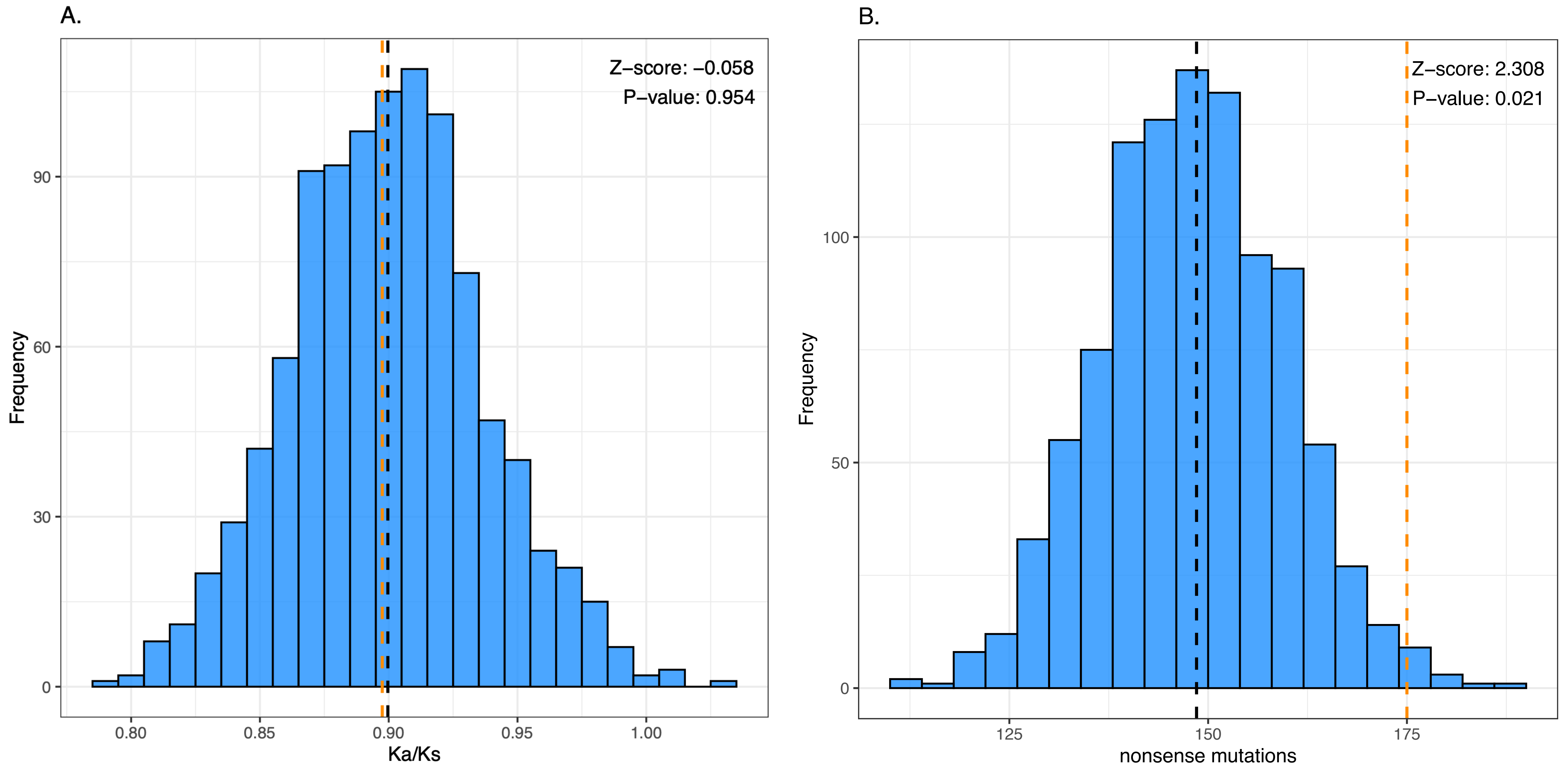
A.
Z−score: −0.058
P−value: 0.954
Frequency
90
60
30
0
0.80
0.85
0.90
0.95
1.00
Ka/Ks
B.
Z−score: 2.308
P−value: 0.021
Frequency
100
50
0
125
150
175
nonsense mutations
