# Supplementary material for: The importance of genes does not predict their mutation rate
Source: PLoS Biol. 2026 Nov 12;24(11):e3004089. doi: 10.1371/journal.pbio.3004089 (PMC13759209; doi:10.1371/journal.pbio.3004089)
Supplement: Supplementary file 24 [file pbio.3004089.s024.pdf]

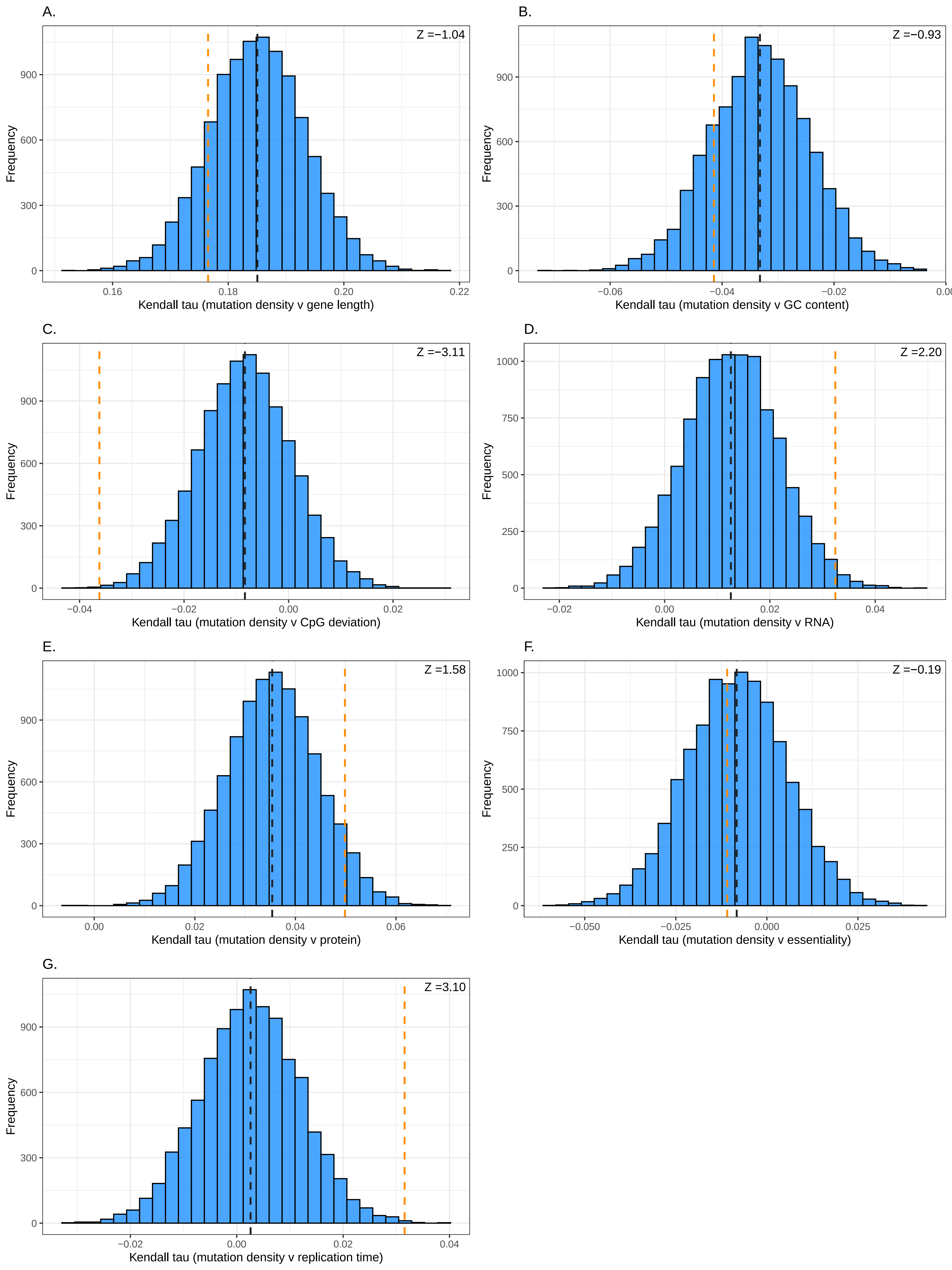
A.
Z =−1.04
Frequency
0
300
600
900
0.16
0.18
0.20
0.22
Kendall tau (mutation density v gene length)
B.
Z =−0.93
Frequency
0
300
600
900
−0.06
−0.04
−0.02
Kendall tau (mutation density v GC content)
C.
Z =−3.11
Frequency
0
300
600
900
−0.04
−0.02
0.00
0.02
Kendall tau (mutation density v CpG deviation)
D.
Z =2.20
Frequency
0
250
500
750
1000
−0.02
0.00
0.02
0.04
Kendall tau (mutation density v RNA)
E.
Z =1.58
Frequency
0
300
600
900
0.00
0.02
0.04
0.06
Kendall tau (mutation density v protein)
F.
Z =−0.19
Frequency
0
250
500
750
1000
−0.050
−0.025
0.000
0.025
Kendall tau (mutation density v essentiality)
G.
Z =3.10
Frequency
0
300
600
900
−0.02
0.00
0.02
0.04
Kendall tau (mutation density v replication time)
