# Supplementary material for: The importance of genes does not predict their mutation rate
Source: PLoS Biol. 2026 Nov 12;24(11):e3004089. doi: 10.1371/journal.pbio.3004089 (PMC13759209; doi:10.1371/journal.pbio.3004089)
Supplement: Supplementary file 26 [file pbio.3004089.s026.pdf]

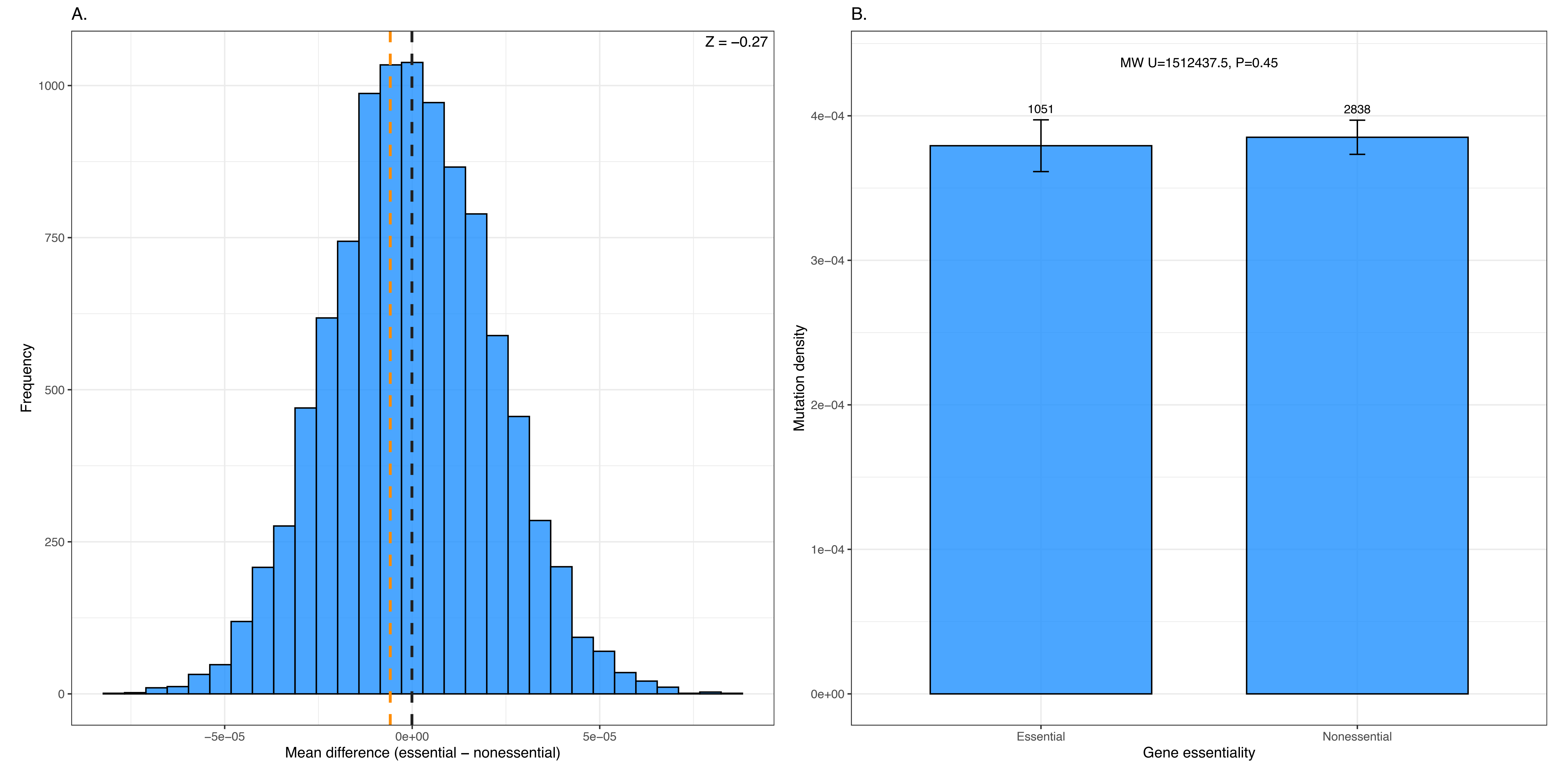
A.
Z = −0.27
Frequency
1000
750
500
250
0
−5e−05
0e+00
5e−05
Mean difference (essential − nonessential)
B.
MW U=1512437.5, P=0.45
1051
2838
Mutation density
4e−04
3e−04
2e−04
1e−04
0e+00
Essential
Nonessential
Gene essentiality
