# Supplementary material for: The importance of genes does not predict their mutation rate
Source: PLoS Biol. 2026 Nov 12;24(11):e3004089. doi: 10.1371/journal.pbio.3004089 (PMC13759209; doi:10.1371/journal.pbio.3004089)
Supplement: Supplementary file 27 [file pbio.3004089.s027.pdf]

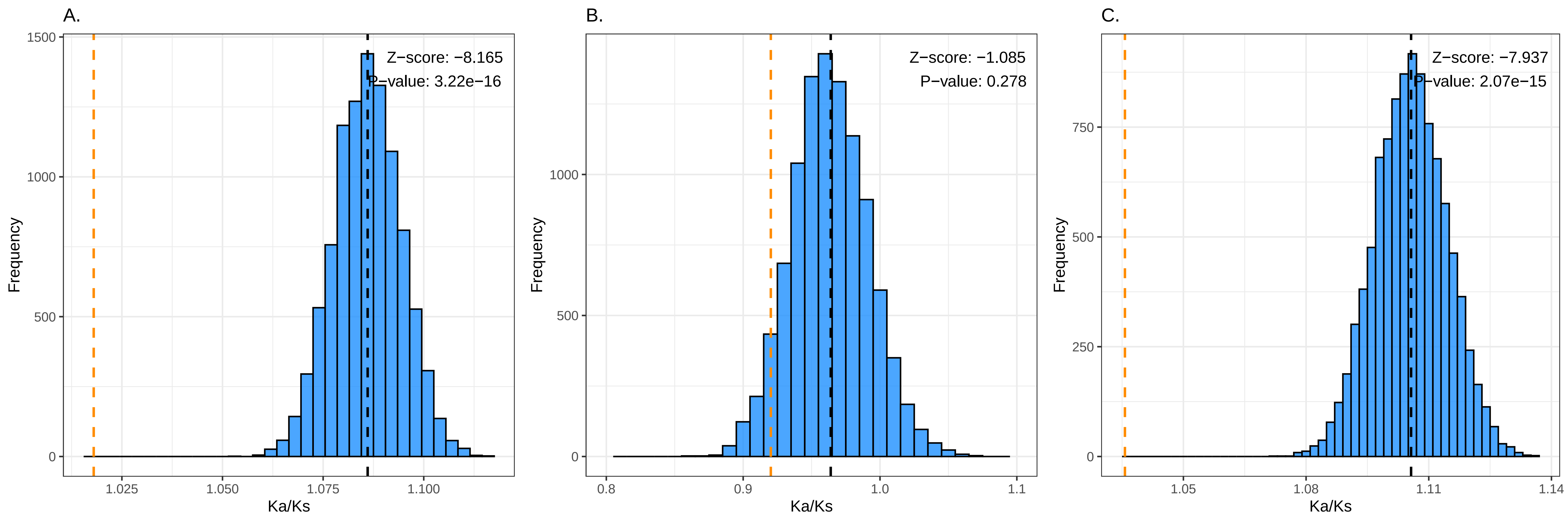
A.
Z−score: −8.165
P−value: 3.22e−16
Frequency
Ka/Ks
0
500
1000
1500
1.025
1.050
1.075
1.100
B.
Z−score: −1.085
P−value: 0.278
Frequency
Ka/Ks
0
500
1000
0.8
0.9
1.0
1.1
C.
Z−score: −7.937
P−value: 2.07e−15
Frequency
Ka/Ks
0
250
500
750
1.05
1.08
1.11
1.14
