# Supplementary material for: The importance of genes does not predict their mutation rate
Source: PLoS Biol. 2026 Nov 12;24(11):e3004089. doi: 10.1371/journal.pbio.3004089 (PMC13759209; doi:10.1371/journal.pbio.3004089)
Supplement: Supplementary file 28 [file pbio.3004089.s057.pdf]

I am satisfied that the authors have sufficiently addressed the issues raised. This includes substantial new content. Nonetheless I am concerned that the way some of this new content is presented is less than fully clear.

Issues in text order:

Text 1 'expression'/'essentiality' 'predicts' does not give the sign of the effect, which seems important (and is given elsewhere in the same column e.g. 'essential genes have lower mutation density')

that permitted a 3-6 thousand generations
confusing wording

"We find transcript abundance to again be negatively correlated with mutational density prior to partial correlation. It is weakly significant on partial correlation if gene length is removed as a parameter but not when it isn't (Table S4)."
Table S4 has -0.07 **** for full correlation and -0.03* for partial correlation between mut_density and LB_transcript. I see nothing in the legend about removing gene length as a parameter and I don't see a non-significant value when gene length is considered (or is 'not when it isn't' meant to mean non-weakly, i.e. strongly significant; this is potentially ambiguous). Please clarify

where only very few genes have any mutations, we approach what in regression would be a flat line
This seems unintuitive – the relationship in question is mutational density against gene length – when one gene has a mutation and everything else has none, that seems maximally non-flat, even if there's no strong expectation for what would happen in a regression (it might average out at flat, but won't that depend on the distribution of gene lengths, so that might not be true?). Do clarify.

While we cannot guarantee an absence of selection in each data set, the sample as a whole is tolerable, although Ka/Ks is low (Figure S18).
If degree of selection is the axis of tolerability (?), what evidence is being used here, apart from Ka/Ks, to put this data above some threshold level? Please be more specific.

In no case out of 10,000 simulations […] hence P <0.001
True but confusing – for a 2 tailed test I might have interpreted that as P≤0.0005. Not giving a P value, although perhaps quoting a threshold from the sampling (e.g. '95% were less than an absolute correlation of x') would give a clearer intuition for how big the observed correlation really is.

the Foster knock out data
Foster uses knockouts of MMR genes, but this phrase is being used (I think) explicitly to refer to the non-MMR gene knockouts from their study. This is clear in some places but not in others – where it's unclear, it's confusing – please clarify throughout.

Results are unaffected by considering expression data from LB growth conditions alone, except that transcript abundance correlates significantly with mutation density upon full but not partial correlation analysis, as also seen when employing the fuller mutation data set (Table S18).

A table should be referenced before the final phrase referring to TS18. I'd have thought Table S4 for LB data, except that there, the transcript-mutation density correlation is significant for both full and partial correlations.

the strand hypothesis

Do be explicit what this is.

the transcript level effect, while remaining negative, is non-significant if we employ the expression data from LB media under Z scored partial correlation

This seems to be contradicted by Table S4 which gives the -0.032 partial correlation 1-star significance.

the transcript level effect is seen if using data from LB conditions (although not on simulation)

Is 'simulation' referring to the Z-test? Could do with being clearer and referencing the display item(s) this is based in.

Text S2

This seems a helpful addition to the piece, but introducing both the methods and results that it represents in the Discussion seems out of place and much less helpful.

the resulting models have almost no accuracy

I'm not sure it's accuracy that's lacking with the low $R^2$, it's explanatory power

In *E. coli*, if protein level is ever significant

Presumably this is referring to the one model (no. 4 in Text S2) where the LB_protein effect has P= 0.013 (?). Would be good to be explicit.
