# Supplementary material for: The importance of genes does not predict their mutation rate
Source: PLoS Biol. 2026 Nov 12;24(11):e3004089. doi: 10.1371/journal.pbio.3004089 (PMC13759209; doi:10.1371/journal.pbio.3004089)
Supplement: Supplementary file 29 [file pbio.3004089.s053.docx]

**Statistical basis of prior analysis**

| **variable** | **claim** | **method** | **ref** |
| --- | --- | --- | --- |
| Mutation density assayed as synonymous diversity | Expression predicts mutational density (higher expression lower mutation) | Wilcoxon test | Martincorena I, Seshasayee ASN, Luscombe NM. Evidence of non-random mutation rates suggests an evolutionary risk management strategy. Nature. 2012;485(7396):95-8. |
| “ | Essentiality predicts mutation density (more essential lower rate) | Fisher’s exact test – proportion of genes essential v mutation density bins | “ |
| Mutation per bp | Essential genes have lower mutation density | Chi squared – we think comparing mutation count to that expected given total amount of sequence in each class | Monroe JG, Srikant T, Carbonell-Bejerano P, Becker C, Lensink M, Exposito-Alonso M, et al. Mutation bias reflects natural selection in Arabidopsis thaliana. Nature. 2022;602(7895):101- |
| Evolutionary rate per bp | Leading and lagging strand genes have different rates (higher on lagging) | Z test | Paul S, Million-Weaver S, Chattopadhyay S, Sokurenko E, Merrikh H. Accelerated gene  evolution through replication-transcription conflicts. Nature. 2013;495(7442):512-+. doi:  10.1038/nature11989. |
| Substitution rate per 4-fold site deviation from mean | Genes for essential processes occur in mutational cold spots | GO analysis of genes in low Z clusters | Chuang JH, Li H. Functional bias and spatial organization of genes in mutational hot and  cold regions in the human genome. Plos Biology. 2004;2(2):e29. |
| Mutation rate per site | Highly expressed genes mutate more | Spearman rank | Chen XZ, Zhang JZ. No gene-specific optimization of mutation rate in Escherichia coli. Molecular Biology and Evolution. 2013;30(7):1559-62. |
| “ | More conserved genes are more mutable | Spearman rank | “ |
| “ | Essential and non-essential genes have the same mutation rate | Mann-Whitney U test | “ |
| Mutational rate per bp | dN/dS is not positively correlated with the mutation rate | Linear rank correlation and multiple linear regression | Liu HX, Zhang JZ. Is the mutation rate lower in genomic regions of stronger selective constraints? Molecular Biology and Evolution. 2022;39(8). |
| Mutational rate per CDS per generation | No association between mutation and gene orientation relative to replication or transcript abundance | Multiple linear regression with gene length as a predictor variable and Monte Carlo simulations | Schroeder Jeremy W, Hirst William G, Szewczyk Gabriella A, Simmons Lyle A. 2016. The effect of local sequence context on mutational bias of genes encoded on the leading and lagging strands. Current Biology 26:692-697. |
| Base changes per CDS | No effect of transcription on mutation | Counts per gene no different from null in high and low expressed genes | Lee H, Popodi E, Tang H, Foster PL. 2012. Rate and molecular spectrum of spontaneous mutations in the bacterium Escherichia coli as determined by whole-genome sequencing. Proceedings of the National Academy of Sciences 109:E2774-E2783. |
| Base changes per CDS for MMR-deficient lines | No evidence that high or low transcription affects mutation rates | Counts per gene no different from null | “ |
| Base changes per CDS (null correcting for gene size) | No evidence that high transcription rates, transcription direction, or DNA strand influence mutation | Chi squared with Yates correction if any number was ≤5 with expectations dependent on gene length | Foster PL, Lee H, Popodi E, Townes JP, Tang H. 2015. Determinants of spontaneous mutation in the bacterium Escherichia coli as revealed by whole-genome sequencing. Proc Natl Acad Sci U S A 112:E5990-5999. |
| Mutation per CDS for MMR-deficient lines | No effect of RNA-seq expression levels on mutation | Pearson correlation and Spearman rank | Foster PL, Niccum BA, Lee H. 2021. DNA replication-transcription conflicts do not significantly contribute to spontaneous mutations due to replication errors in Escherichia coli. Mbio 12:e0250321. |
| Mutation per CDS for MMR-deficient lines | No effect of strand orientation on mutation | Student’s two-tailed t-test and Mann-Whitney test with Benjamini-Hochberg correction | “ |
| Intronic substitution rate per bp | Mutations tend to occur at highly expressed sites | Spearman rank correlation, partial correlation and multiple linear regression | Park C, Qian W, Zhang J. 2012. Genomic evidence for elevated mutation rates in highly expressed genes. Embo Reports 13:1123-1129. |
