# Supplementary material for: The importance of genes does not predict their mutation rate
Source: PLoS Biol. 2026 Nov 12;24(11):e3004089. doi: 10.1371/journal.pbio.3004089 (PMC13759209; doi:10.1371/journal.pbio.3004089)
Supplement: Supplementary file 30 [file pbio.3004089.s054.docx]

**Adaptive model summaries**

***E. coli***

1. ***E. coli -* Full expression data (log transformed)**

2. ***E. coli -* Full expression data (untransformed)**

3. ***E. coli -* LB expression data (log transformed)**

4. ***E. coli -* LB expression data (untransformed)**

***S. cerevisiae***

1. ***S. cerevisiae -* Full expression data (log transformed)**

2. ***S. cerevisiae -* Full expression data (untransformed)**

**Full model summaries**

***E. coli***

1. ***E. coli -* Full expression data (log transformed)**

2. ***E. coli -* Full expression data (untransformed)**

3. ***E. coli -* LB expression data (log transformed)**

4. ***E. coli -* LB expression data (untransformed)**

***S. cerevisiae***

1. ***S. cerevisiae -* Full expression data (log transformed)**

2. ***S. cerevisiae -* Full expression data (untransformed)**
