# Supplementary material for: The importance of genes does not predict their mutation rate
Source: PLoS Biol. 2026 Nov 12;24(11):e3004089. doi: 10.1371/journal.pbio.3004089 (PMC13759209; doi:10.1371/journal.pbio.3004089)
Supplement: Supplementary file 31 [file pbio.3004089.s058.docx]

REVIEWERS' COMMENTS:


Reviewer #1:

In their manuscript entitled "No evidence that genic importance predicts mutation rate variation ", Sofia Radrizzani1 and Laurence Hurst reanalyse mutation accumulation experiment to invalidate recent calls suggesting that local variation of mutation rate may be subject to selection.

This theme is important, because the idea that the control of mutation rate may be to some extant smarter than random noise has been always a tempting. On the one hand, most microbiologists that have no clue about evolution love the idea that mutation rate is controlled to create variation just when needed. For instance stress induced mutagenesis is necessarily a strategy to find mutations to resist to stress. Over the last decade, another type of scientists have tackled the question from the perspective of essential genes, this time having a lower mutation rate than the rest. While these types of selection in mutation rate is possible, it is extremely weak, and there are usually way stronger forces at play to explain the observed patterns (we debated that already in 2004 (Tenaillon et al Trends in microbiology 2004) .

**Response:** While we are not focused on the stress-mutation debate as in the 2004 paper, we think this provides a broader context that we hope will make the paper that bit more appealing to microbiologists. We thus write:

*“There have been two important related debates concerning the non-random nature of mutation. On the one hand are issues as to whether, especially in a microbiological context, the association between stress and mutation is adaptive [7]. The second, and the focus of the present analysis, is the broader possibility that variation in mutation rate between genes in a genome is the result of selection (e.g. [1-3]).”*

Here, the authors engage in this debate on the ground of the statistical analysis and type of data used to do these inference. After years of analysis of weird mutation reporters that created complex patterns, the gold strandard has become to study genomes of mutation accumulation. But due to the lack of mutations, scientists have used mutator lineage to make some of their derivations. Here the authors dig further un teh statistical baises inherent to the analysis of MA and contrast mutator and non mutator lineages. They show that in E. coli many hypothesised drivers of mutation rate are not relevant, exept expression at RNA level.

Overall the paper is very interesting, may be a bit too technical at some moments, which may make a class of the targeted readers (the microbiologist) less prone to get the message.

Here are some comments:

In the introduction, i think, it would be worth mentioning the intensity of selection applied to a control of mutation rate. TO mention that though a local control is not impossible, it remains tricky for natural selection to act on a local gene, unless the mechanisms affect a large panel of genes.

**Response:** We very much agree with this point and thought that we had addressed it writing:

*“While adaptive models can be questioned on the basis of whether selection can ever be efficient enough to modify mutation rates on a limited sample of genes [7, 18],…”*

We have now additionally added mention of the same problem to the discussion.

Page 4: We are unaware of any analysis attempting to control for all putative variables.
on the control of essentiality of genes and their impact of mutations found in these genes we found an effect while controlling for multiple factors in Vigué and Tenaillon (PNAS 2023) Where we showed that essentiality play a role in the purge of deleterious mutations more than expression over short time scales.

**Response:** This paper very nicely supports the difficulty that we mentioned in claiming that essential genes have a lower mutation rate, namely that such genes are also likely to eliminate mutations earlier and hence just appear to have a low rate – we now cite it in this context.

RNA seq median zero is not a great names for tables and discussions.

**Response:** We agree it is not – now changed

Relative to the RNAseq data, it could be worth knowing the overall types of environments sampled. Are there for instance a large number of stress conditions.

**Response:** While the source files for the RNASeq didn’t mention the experimental conditions, we agree this would be of interest to the reader and so now provide a supplement in which we extract from the SRA archive the study title. We present this alone with the projectID, the bioproject id and the experiment reference. Of 3376 studies 152 mention the word “stress “in their titles. We also provide the webcrawler for the data extraction.

We note however that growth conditions can affect which genes are expressed and so now repeat the analyses using the RNASeq data from LB conditions for which the original negative correlation between expression and mutation was made. We find that results are unaffected.

In the text the impact of RNA or protein abundances have different sign effect on gene length, this is not intuitive to me given they have the largest positive correlation.

**Response:** We agree that this might appear odd. But the effects with respect to mutational density are weak and we would expect that “fragile” effects might sometimes change direction because they are close to r=0. Indeed, when we restrict the protein data to experiments done under LB conditions, the protein effect goes away.

I liked the control for gene length. Could a small simulation model tell when this type of artefact matters. I expect taht the signal should disappear when the number of mutation is large enough. That would be a valuable add on for people analysing this type of data in the future.

**Response:** Thank you for this comment. We have performed simulations in which we randomly allocate N mutations to E. coli’s genes and then calculate tau between mutational density (number of mutations per base pair of gene) against gene size. The underlying distribution should show no correlation as mutation positions are selected at random. Nonetheless, reinforcing our prior finding, positive correlations are observed these being strong when N is relatively small. With N in 1000-5000 domain tau in the region 0.15 is observed. The artefact is, we also show, stronger for the more commonly applied statistic, Spearman’s rho.

To me the main result of the E. coli part is that RNA level is associated to low mutation rate. As the author mention this is likely a combination of mfd coupled to MMR that results in excellent repair. The point is that, this type of observation need to be better debated to me to counteract the selectionist agenda. Indeed, we could here just use the argument that natural selection has favoured a repair mechanism that is more actif on highly expressed genes and indeed rather than just correcting for the mutagenic effect of transcription results in an even lower mutation rate. Moreover as the mechanism is generic and not gene specific, the selection for such mechanism is more possible. Furthermore as we published ( Feugeas et al MBE 2015) in a quite tough paper, (but the discussion is worth reading as we anchor on the same papers), under stress conditions MMR and mfd expression decline, which lead to what we think is this time a positive correlation between expression and
mutagenesis, but only for genes expressed under stress.

**Response:** This is indeed a very interesting result and stimulated us to reappraise the correlations seen in the MA lines grown under LB conditions (i.e. no stress) using expression data just from benign LB conditions. We recover the same result as before.

This is purely mechanistic, and while i think selection to lower mutation rate overall exists, the bacterial mutation rate is extremely low, the selection to increase it to find the best adaptive mutation is very unlikely at play here. But nevertheless if one look from a distance this interplay between expression of MMR, mfd and their super impact according to conditions could easily be fully interpreted in a selectionist agenda. This one would be more credible as only broad interactions would be at play and have genome wide effect upon which selection is more likely to play (for the lower rate, i am still not convinced that there is any recurrent engouh conditions where mutation is teh solution to stress to promote a signature of mutation rate that is favoring high mutation rate under stress). I think that the discussion should adress in a bit more detail these scenarios in saying how they match or not. The fact that
the focus is on essentiality or protein abundances is to me moving away from this simple observations. For decades, molecular evolution could not distinguish between highly expressed and essential, (though as i mentioned we found a signal, having 80000 genomes and looking at very short time scale), so saying that essentiality is not popping up as a driver of gene importance may not be the strongest point. I think the last part of the discussion about the effect size may be better than saying a rejection of the hypothesis.

**Response:** We agree more discussion is helpful. We certainly agree that a simple model whereby more transcription means more repair works in E. coli and can be configured as a selectionist explanation. As multiple genes would be affected by such a system one can imagine selection more effectively operating. We now clarify that we think it most important to discriminate between two questions: how can we account for differences in mutation rates between coding and non-coding sequences (not the focus of our analysis) and how to account for variation between CDS in mutation rates. It is this second question that is much more contentious as the strength of selection associated with tweaking a gene’s mutation rate up or down is miniscule, while tweaking downwards all CDS is potentially quite strong selection.

We think we also agree that interpreting the low mutation rate of more transcribed genes as being adaptive is problematic. Beyond the strength of selection argument, there are three issues with such an interpretation. First is why we don’t see an effect at the protein level. This suggests that, were this the result of selection it isn’t moderated by its effects on the most common products. Second, why do we not see the same effects in yeast? In many regards this is the most important objection as both species have efficient selection. We thus rather agree that these weak correlations more likely reflect taxa specific mutational proclivities than something crafted by selection. Third, the effect sizes are very small. We agree that had we selected just the one result (higher transcription -> lower mutation rate) we could have published a high profile paper claiming selection operating on mutation rates (this was Martincorena’s claim). But as the other referees note, the core result is really not a simple yes/no rejection but rather more nuanced – if there is an effect there, it is both of very small effect size and not consistent. It certainly doesn’t challenge any understanding of the evolutionary process. We have adjusted the abstract and discussion to reflect this.

The part comparing the different patterns in MMR, is very informative, i think people underestimate the type of substractions we have to do when a system is lacking and what does its absence means for the mechanisms at play in replication and repair. Reminding this loud and clear is more than necessary.

**Response:** Thank you. This we also think is a helpful contribution to the debate

The yeast data nicely confort the overall pattern. I am not as aware for yeast, but what is the mechanistic hytpotehes for the late expression gene high mutation rate and what type of genes are epxressed in these conditions.

**Response:** As regards mechanisms we thought we covered this saying “ *A diversity of possible mechanisms has been suggested including a switch in the repair machinery [32, 37, 38], nucleotide exhaustion [39] (cf. [40]), or altered methylation [31].”*

As for which genes are involved, the table in the supplement suggests a weak effect whereby more highly expressed genes are expressed earlier in the cell cycle.

Overall, i think the author have pointed out important limits to the mutation rate selectionist hypothesis. Papers supporting these hypothesis are always welcome in fancy journals because adaptationist hypotheses are always more attractive than noise. Paper that debunk these data are less sexy by definition: more technical and negative; but it is key to publish them in high impact journal such as PLoS Biology to give support to thorough science.

Olivier Tenaillon (I always sign my reviews)

**Response:** Thank you for your thoughtful and helpful critique

Reviewer #2:

This is an impressively thorough dissection of potential correlates of mutation in the two key model microbes where the range of published data is sufficient to do so. It needs to be thorough, because the authors attempt to demonstrate a negative - a lack of a role for gene importance in among-gene variation in mutation. This is, a priori, a hard thing to do and at one level impossible. This means that in many ways the most robust part of this study (that should perhaps get more prominence) is their final discussion point - that even if there is an effect of gene importance on mutation rate, that effect must be very small relative to other effects.

**Response:** This point is very well taken and have reworded to clarify that the big issue is that effects sizes are so small – and inconsistent in direction – that selection operating on the between-gene variation in mutation rate seems next to irrelevant (and hence don’t meaningfully challenge an orthodoxy). We have further examined alternative means to determine the effect size of the predictors and find these to be miniscule.

Among secondary findings, the biasing effects of using mutator strains seems particularly worthwhile (and perhaps deserves further discussion in the context of inter-organism evolution of DNA repair). Nonetheless, the dissection of correlations is the core and clearly focused against adaptationist hypotheses of mutation rate
variation. Such hypotheses are remarkably pervasive and tenacious, making this assessment of evidence used to support such hypotheses important, worthwhile and very appropriate to the journal.

**Response:** Thank you for the support. As you note we appraise not just the results but also the methods which motivates some of our choice of methods too – which we will get onto….

The approach taken is a somewhat idiosyncratic mixture of sequence randomisation approaches that are both powerful and elegant, with (partial) correlation approaches that are neither of those two things. The large number of distribution plots and correlation tables arising are not the most user friendly (particularly in interaction with the journal's bitty approach to supplementary material), but are at least a consistent interface for the reader.

Issues arising which roughly fall into more and less important

More important:

Better justification of the (partial) correlation approach would be helpful - it feels like a linear modelling approach, perhaps with a binomial response of mutation/not, accounting for interactions among multiple explanatory variables (or even a machine learning approach), would have been both easier to grasp and more powerful. It would bring in assumptions about (lack of) error in explanatory variables, which would undoubtedly be an issue for the expression data (only), but error there (particularly the potential for different amounts for RNA and protein data) is an issue for the logic as it is (and something the authors address).

**Response:** Please notice that our core results are not those derived from the correlations and partial correlations as these we established to be biased. We used that approach in the first instance as it both matched what had been done before and seemed to suit the data (distributions of many variables are ugly). The core results are the Monte Carlo Z tests, as we emphasise in the abstract. We would strongly defend them as, unlike any glm methods, these uniquely permit us to control for the bias in mutability of different trinucleotides. And note that this is a profound effect – there is over an order of magnitude difference in the mutability per occurrence of a trinucleotide between the most and least mutable (see e.g. fig 5). Given this, we think a simulation approach to derive null expectations is much to be preferred compared to off the shelf methods.

Further, part of the motivation of this paper is to try and determine why results have been so mixed and confusing. As we show use of MMR data seems to have caused problems. But might the statistical approach matter? All approaches prior to ours have not used count data but, for obvious reasons, used counts per bp to remove a trivial predictor of counts (longer genes have more mutations). They also typically use non-parametric statistics as the predictor variables are not nicely distributed: gene expression data typically has lots of zeros and a very long tail. They typically use Mann Whitney U test for pairwise comparisons or Spearman correlation, these being much less sensitive to the form of the distribution. We provide a summary of prior methods. On realizing that non-parametric approaches have been the preferred method and then seeing that these indeed have issues, we thought it useful to incorporate this as part of the critique. Indeed, in this context, to come close to attempting a replication of results using modern gold standard data, it seems to us beneficial to accord with the models of analysis provided by prior adaptive claims and rebuttals (none of which use count data).

We would go further and suggest that our approach using trinucleotide controlled simulations is a strong approach. Monte Carlo simulations like this make almost no assumptions (unlike parametric approaches) and we employ tau simply as a null measure – the actual result is Z by tau. In many fields such simulations are considered the gold standard model of analysis. We could here have employed any non-parametric correlation approach, but tau is still probably the best. We now indeed show this using rho as the null variable: Z by rho gives the same results. We are happy to defend this approach as it uniquely considers the local sequence context of mutations, accords with gold standard that is Monte Carlo simulation and directly relates to prior approaches. As requested by the referee we now motivate this all much more fully in the introduction as no doubt others too might be curious about our choices.

This being said, we have implemented either Poisson or negative binomial regression (depending on whether overdispersion is or is not seen) and report these results. They support our central claim that the putative adaptative parameters explain almost no variation while well described effects, such as replication timing in yeast, are robustly recovered.

We explain a bit more issues with application of negative binomial below in response to referee 3.

As is made clear in the first sentence of the discussion, there are two adaptive conjectures leading to opposite expectations for 'important' genes. To be a fully convincing refutation of such selectionist ideas it does feel that the authors would need to take more seriously the possibility that both things are going on at the same time, leading to their observed lack of clear correlations of mutation density with 'important' genes. That could mean more complex/comprehensive models or greater clarity over/dissection of 'important', but at least some discussion would be helpful.

**Response:** We apologize for seeding confusion: the two conjectures are not opposed but we can see why our writing might have given that impression. To see why they aren’t opposed, consider the Ka/Ks v mutation density analysis. The advocates of the “high mutation rate for rapid adaption genes”, suggest that selection should favour higher mutation rates for proteins that have a high rate of evolution (high between species Ka/Ks), while the highly expressed/essential gene arguments argue for lower rates of mutation for genes that are under stronger purifying selection (hence slow evolving, low Ka/Ks). Rather than being in opposition, they are just two sides of the same coin, both predicting that between-species Ka/Ks should be positively correlated with mutational density – which it isn’t. However, we see we have been confusing as only the latter do we regard as more “important”. But this is of course a problem with what “important” ever means. A fast evolving immune protein could be every bit as important as a slow evolving key protein essential for cell cycle for example, but classically (and as we employ the term) only the latter is deemed “important”). We have rewritten in the hope of avoiding such ambiguities.

The finding that a rank-based statistic can get messed up by lots of repeated values (in this case zero mutation counts) doesn't feel very novel, but perhaps deserves the given highlighting in this context. It would also seem to add weight to the currently un-discussed possibility of using a GLM where lots of zeros in a Poisson or Binomial model are very much normal.

**Response:** We hoped we addressed the possibility of using count data above (see also below results for negative binomial models that we now add). We were slightly surprised by how big an artifact the correlation approach generated, not least because Kendall’s tau is supposed to be resilient to such effects. Spearman’s handles ties in a much less robust way but has been the method of choice. We now perform simulations – following the suggestion of referee 1 – to see how both Kendall and Spearman show artefacts as a function of the number of mutations. Spearman’s is indeed more affected. We hope we have addressed the issue of using count data above.

The response variable here is 'mutation density', a phrase which is used pretty much everywhere except the early parts of the Methods, where we need to know exactly what it is and how extracted from the data described.

**Response:** The referee is correct in calling us up for this. We now define mutational density in the abstract and on first mention in the introduction. We were trying to avoid the term rate as many assume that such a measure has a temporal dimension which is not relevant here. However, rate is also used without a temporal dimension, just to add to the confusion. We now write:

*“To address the central problem of whether genic importance predicts mutation rate we start by considering the number of mutations in each gene normalised by gene size, giving a per base measure (as done by all prior analyses: S Text 1). This metric we call mutational density* *so as to avoid any possible confusion with some usages of the term mutation rate as the later may imply a temporal dimension (note the above usages of the term mutation rate are equivalent to our mutational density having no temporal dimension)”*

More generally it would be helpful to connect it precisely to (different) definitions of mutation rate, which is what is invoked in the title.

**Response:** As it happens none of the prior papers had a temporal dimension and all employed what we call mutational density, but still called it rate. We have just found in the past that referees don’t like us talking about a rate when there is no temporal dimension (though tax rates, for example, are not per unit time either). We hope the above clarifies i.e. if two genes have the same mutational density – the parameter we measure- they will also have the same rate if that means mutations per base pair or if it means mutations per base pair per unit time/generation.

Probably related to this, different numbers of generations presumably occurred in the different MA studies (and perhaps within them?), so saying explicitly how this was dealt with and/or what effect that has on the possibility of interpreting the findings seems important.

**Response:** As we aren’t looking at per generation rates no direct correction is needed. Where correction is needed is in comparing mutations called in the .2 and the .3 versions of the genomes as their parameters are very slightly different. We had mentioned how we dealt with this – by taking weighted means. See methods.

Statistical power and (non-) significance are important here, but the first section of the Results asking about evidence for selection in the MA data used, seemed the least effective at dealing with them.

**Response:** We agree – which is why we did the others. The first test, however, is unique in using all the mutations.

Specifically there is a clear hypothesis that, if there's selection, mutation by position would be "3 > 1 > 2", yet we are never told what the best estimate of the observed order is (we should be, even if there are only 6 options and no statistical significance!). We only have what feels like a very under-powered test (multiple instances of a paired Wilcoxon test) coming out as non-significant.

**Response:** We agree that this is an underpowered test largely because N=64 no matter how much data we have. It’s unique advantage is that it employs all mutations in CDS. However, we think the referee may have slightly misconstrued the test, owing to our imprecise writing. We don’t seek to have one value for the rate of mutations at sites 1,2, and 3 thereby generating a rank order. This would confuse differences in mutability by position with differences in mutability because of nucleotide content differences at the three sites (which are inevitable). Hence, we generate a 64 element vector for each site representing the mutation rate per central base of each possible trinucleotide. We then perform trinucleotide matched comparisons between the 64 elements at each pair of sites. We now add to explain:

*“…If, by contrast, we are recovering mutations in an unbiased manner, we expect all sites to have comparable mutation rates (1≈2≈3).*

*There is, however, a complexity namely that if the three sites differ in their nucleotide content and nucleotides are differently mutable (both of which are true), then average rates of mutation at the three sites may differ but not because of the codon location per se but because of the difference in nucleotide content. To overcome this problem, we consider the trinucleotide context of all mutations and consider, for each site, the mutation rate of the central base the trinucleotide centred on the given codon position, per occurrence of that trinucleotide at that position across all CDS. We can then perform a paired test considering the differences between the 64 trinucleotides at different sites. Rather than asking about a single absolute rate at each of the three sites we thus instead consider the distribution of the differences between the two paired 64 element vectors.“*

Although we probably shouldn’t, given the forms of the distributions and their failing normality tests, given the request from referee 3, we now also report the mean and sem of the differences. We hope this addresses this concern.

This questionable approach is coupled with a lack of engagement with others' work that does find evidence of selection in mutation accumulation - I'm thinking of these papers, though there may be others:

Mahilkar, A., Raj, N., Kemkar, S. & Saini, S. (2022) Selection in a growing colony biases results of mutation accumulation experiments. Scientific reports 12, 15470. 10.1038/s41598-022-19928-5
Wahl, L. M. & Agashe, D. (2022) Selection bias in mutation accumulation. Evolution 10.1111/evo.14430
These issues should be addressed more directly.

**Response:** We don’t think it fair to say that we didn’t engage with the prior literature. Prior analyses have employed a single nucleotide version of our second test (Ka/Ks) to ascertain whether purifying selection is discernible – Foster does this, as do Monroe et al for example. We were however remiss not to cite them in this context. This being said, we think our tests 1 and 3 are novel.

The key question for us is whether we see evidence that we are missing a good number of mutations owing to negative selection which would make tests of whether essential genes have a low mutation rate uninterpretable. In different contexts you might want to go further. For example, if you want an estimate of absolute per generation rates of mutation then some sort of correction may be required (e.g. <https://doi.org/10.1038/381694a0>) even if the Ka/Ks is not significantly different from 1. Notwithstanding that when such corrections are done the adjustment is commonly small (e.g. <https://doi.org/10.1098/rstb.2009.0287>), this is not our question as we are just considering relative densities, not absolute rates. Similarly, we are not trying to estimate the DFE or the rate of positive selection. Indeed, in our context we aren’t worried about positive selection (as suggested by the stop codon analysis) as this is the opposite of mutational purging.

The papers provided by the referee are relevant to our study in so much as they motivate the need to test for selection operating in MA lines. We now cite them in that context. Beyond that, they are not especially relevant to our study. The first of these models the possibility that there may be positive selection operating on MA lines. The second shows very nicely that MA data may skew DFE estimation. But we are not attempting to estimate the DFE and want only to know whether purifying selection may have caused us to miss enough mutations which could then give a false positive signal of lower mutation rates in essential genes. The tests suggest that we are not missing many deleterious mutations (we also see that essential genes have the same rate as non-essential genes so the opportunity for misinterpretation is also gone).

Less important

What is and isn't a 'good' correlation could usefully be more consistent - there is one paragraph where two different correlations, each of 0.3 are referred to very differently. This does correspond to the visual impression of the correlations (S17A seems a clear overall relationship, 5B does not), but just being more explicit about that might help.

**Response:** We agree that what we wrote looked inconsistent. The interpretation of the differences in tau values is however complicated by differences in sample size of mutations. The Foster WT sample for example is only ~200 mutations while the MMR set runs into tens of thousands. Two similar correlations where one has a much reduced sample size are hard to properly compare. To obviate this problem and provide “fair” comparisons, we now just consider three classes of mutation (deltaMMR, WT and Foster’s nonMMR KOs) and simulate to match sample sizes. We have thus assembled a pure wild-type mutation collection of around 1400 mutations. We compare the profile in that set to that seen in the deltaMMR set and the nonMMR KO set. This at least controls for the sample size variation that existed before for the various WT sets. As can be seen, the Foster KO data correlate well (tau =0.56, rho =0.75) with the WT data while the MMR KO data do not. We perform simulations to show that they are indeed different allowing for differences in the number of mutations in the y axis variable. We hope this makes the presentation of these results fairer, more consistent and more easily interpretable.

The orthogonal regression lines in the figures don't seem to add much, beyond hinting at the idea that these data aren't all that linear, and that allowing variation on both axes doesn't get round that issue or the issue that outliers can have undue influence on regression lines. A 1:1 line (where it intersects the region) would seem more helpful.

**Response:** We think it is the very strangeness of the orthogonal regression line in 5B that is notable. We now comment on the distortion of the line in 5B. The MMR rates are much higher so a 1:1 line would not be appropriate.

Ref 99 is used for duplex sequencing, but isn't about that - should it perhaps be this?: Bhawsinghka, N., Burkholder, A. & Schaaper, R. M. (2023) Detection of DNA replication errors and 8-oxo-dGTP-mediated mutations in E. coli by Duplex DNA Sequencing. DNA repair 123, 103462. 10.1016/j.dnarep.2023.103462

**Response:** We are much appreciative that the referee spotted this error

The findings of very different characteristics of the data from the two labs is intriguing - could the authors speculate about any methodological differences between the studies (different media leading to different Ne??)

**Response:** We suspected it might be a difference of the time span of the experiment, however this doesn’t seem to be the case. We now write:

*“We do not know why there is apparent difference between the Wei and Foster data sets. One possibility is that if an MA line is run for longer the opportunity for selection might be higher. However, the Foster data comes from experiments that permitted a 3-6 thousand generations, while the Wei data comes from MA lines of about 1500 generations.”*

It seems a little wrong to introduce novel findings about two new species in the discussion. Could this be added to the end of the results?

**Response:** We agree and have moved.

Probably published since this was submitted, but this paper seems to be another reanalysis of much of the same data, although addressing different questions, so some assessment/discussion might be appropriate: Jago, M. J. et al. (2026) Extended sequence context shapes mutational bias in Escherichia coli. Proceedings of the National Academy of Sciences 123

**Response:** We indeed noticed this paper since ours was submitted. However it seems mostly to be concerned with large numbers of mutations from repair knockouts which as we show seem to be different. Where it is a little concerning is that it suggests the possibility of much longer range effects beyond the trinucleotide context. We have added this as a caveat.

It's somewhat confusing to have Ka/Ks methods given their own section, but then also appearing with a much more cursory description referring to method details elsewhere (ref 64) - if these are different sets of data, the relationship should be clearer.

**Response:** We aren’t sure but think there may be a confusion here. We use Ka/Ks to test for selection on the spontaneous mutations (start of results) and also look at Ka/Ks from between-species comparisons this naturally showing strong evidence of purifying selection. These values were precomputed and given in ref 64. We clarify this in the methods and add a note that the MA line and between-species comparisons are different.

Particularly in the abstract but potentially elsewhere too, using 'replication' and 'replicatory' in adjacent sentences to apply to completely different things is confusing. Do rephrase.

**Response:** We have rephrased to clarify

Not sure if it's a typo or my mis-reading, but 'explain less than 0.5% of the variation…R2 = 0.049' seems wrong.

**Response:** It was a typo – missing a zero after the decimal for the R^2^


Reviewer #3:

Comments on Radrizzani and Hurst "No evidence that genic importance predicts mutation rate variation"

In this interesting manuscript the authors reconsider the question of whether the mutation rate in genes is affected by the importance of the gene using data from several mutation accumulation experiments. They focus primarily on E.coli since this is one of the species for which claims of adaptive mutation rate variation have been made, and for which there is a lot of data. They show that there is little evidence of natural selection acting upon the accumulated mutations. They then demonstrate that there no evidence that more important genes have lower (or higher ) mutation rates when various confounding variables are taken into account.

**Response:** Not just when confounds are taken into account – we see no evidence with or without covariate control.

While I think these are interesting results, their statistical approach is not appropriate and they find that the method is biased with respect to gene length. This does not surprise me - a rank correlation is going to struggle with this type of data.

**Response:** We didn’t motivate the choice of test well enough, which hopefully we now do. It may well not surprise the referee but, in part, the paper is attempting to dissect the possible problems with the results that have been previously claimed and prior claims were based on partial and non partial non-parametric correlation analysis. They in fact tended to use Spearman rank to predict mutations per bp of CDS. As we predicted, and now show, Spearman rho is more affected than tau.

The solution is to use a general linear model with a Poisson distributed error. This is a standard method for dealing with count data, and if they find that their error is overdispersed then they can use negative binomial regression. This approach has lots of benefits - it is a well-established method with well understood statistical properties, fixed effects (such as leading/lagging strand) are easily accommodated and critically they can get standard errors on the estimates so they can establish whether they actually have the power to determine whether there are effects. The effect of gene length will be treated properly (I think gene length needs to included in the model as the log of gene length because in Poisson regression
the log of the mean of the mean of the Poisson is modelled as a linear combination of the independent factors in the model).

**Response:** We certainly agree that there are alternative approaches that have some advantages. But they also have disadvantages. As we responded to referee 2, we think a major advantage of our simulation approach is that it uniquely deals with between-gene variation in trinucleotide abundance, the mutability of different trinucleotides being such a profound effect – over an order of magnitude difference between the most and least mutable.

Nonetheless, motivated by the comments of the referee we did apply count based statistics, cognisant that they don’t control for trinucleotide effects. First, we applied a simple poisson model with all our variables and log(gene length) as a predictor. We then calculated the dispersion ratio as square of the Pearson residual fit divided by the residual degrees of freedom. For the E. coli data this was high, indicating overdispersion and the model failed “dispersiontest” from the AER package (p=0.0003). Hence a negative binomial is preferable (on raw counts the variance is about twice the mean too). On fitting the negative binomial we then in turn asked whether a zero excess model is needed (check_zeroinflation from R package performance), using simulated residuals – we find zero excess is not needed. For the negative binomial models we see no evidence that the log(gene size) estimate is different from one, suggesting that a doubling in size gives a doubling in mutation count, so set this instead as an offset variable.

After this is where we met with a few issues, the results being somewhat sensitive to implementation. First, both protein level and RNA level are, as usual, really ugly distributions – lots of zeros and a long high tail. We can do the usual common transforms for expression data, either log2(level +1) or log10(level +1). Below we give the log10, but log2 is similar. We then ran two models without interaction terms, one with the log10 (level +1), one with the raw data. Unfortunately, core results change a little depending on how the data is handled. Notably, if logged, the expression parameters are not significant, if not logged, RNA level is on the edge of significance and has changed sign from the unlogged case. Naturally, with our rank based approach this cannot happen.

We also consider two gene expression data sets, one the full set and one specific to LB medium, these two correlating well (~0.7). We find on Z analysis of the partials for the LB data set that no variable is predictive. With the LB data in negative binomial analysis the protein effect is weakly significant if not logged but with a positive sign (logged it is not significant but negative) this implying more protein giving more mutation, the oppositive of the adaptive models.

In a comparable analysis of the yeast data we recover the replication time effect no matter how we handle the data.

While this seems rather chaotic, there are a few things we think are useful take homes from this, namely, a) the direction of any expression effect is highly fragile with the negative binomial approach giving only a positive correlation, if ever significant, contra the adaptive models, b) more importantly, if we calculate Nagelkerke’s pseudo R2 for a model with strand, essentiality and protein level, and take the average across implementations (expression data, logged or unlogged) it is just 0.002. For the yeast data (essentiality and protein level) it is smaller still. This we think is in many regards the real take home story – if there are any effects, they are so miniscule as to be biologically irrelevant. By contrast, robust effects like the yeast replication timing effects are seen no matter how we handle the data. We now add a few paragraphs in the conclusions where we address these issues. We also provide interactive R scripts for users to explore the model options (we haven’t here discussed the full interaction model for example).

So, the analysis should be redone using this approach; this will simplify the manuscript eliminating lots of unnecessary simulation and analysis. One could even compare the results for the MMR-deficient and wild-type strains by including a fixed effect and interaction terms. redoing the manuscript using this technique will not take long.

**Response:** to reiterate, while we very much appreciate the referee’s point that a glm approach using count data has a solid theoretical basis, our simulation approach has many advantages in the current context, in particular that it allows for the high variation between trinucleotides in mutation rates.

It is unclear why they focussed on the data from Foster and Wei, particularly given that the Foster data comprises data from strains with other repair genes that have been knocked out.

**Response:** We did not make it clear that we wanted to use samples large enough to perform meaningful tests of selection on (see also below). There are only two really good sized sets of mutation data from individual experiments.

As regards usage of Foster KO data, we were following the prior precedent set by Foster et al (2015) who retained all lines that had the same mutation rate and spectra as WT. We don’t think we emphasised that the spectra they also showed to be the same, not just rate, as we now confirm. Naturally, there comes the obvious objection that her KO lines may have misled us, hence the analysis of the smaller sample of WT sensu strictu which confirms that they did not. Given the prior evidence and this confirmatory test, we think the usage of the alternative (non MMR) KO lines is easily defended. We now expand the “pure” WT lines to include three smaller WT data sets (beyond the Wei and Foster WTs).

They show the mutational profiles of these KO strains are correlated to that of the wild-type strains but the correlations are not particularly strong and only slightly stronger than the correlation to the MMR strains which they have shown give different results in their main analysis to the WT strains. Are they as strong as we might expect?

They could address this with simulations.

**Response:** As noted to referee 2, it isn’t trivial to compare between analyses when the number of WT mutations differs between data sets considerably. To overcome this problem, we now just employ the largest WT data set possible (see below) and perform simulations to ask whether MMR and non MMR correlations with WT are different. In the comparisons (WT v Foster KO and WT v MMR), the first has a correlation of 0.56 that latter 0.3 on Kendall tau (the former is 0.75 in Spearman rho which I hope the referee agrees is particularly strong!) As suggested, we show by simulation that the two are different, allowing for sample size differences between MMR and Foster KO.

Whether the WT v Foster KO correlation alone is as “strong as we might expect” is rather harder to know how to appraise. Where would we obtain a null value from? We can subsample from the WT data with replacement picking the same number of mutations as seen in the Foster KO set, determine the trinucleotide densities for the subsample and correlate against the observed WT. Repeating this 10000 times we can then ask what the mean tau would be if the WT data were correlated against itself but with matching sample sizes as in the observed correlation. This is not like the above test and instead is a bootstrap that should not be used to test for significance as we are just correlating something against itself but allowing for sample size differences. In the actual correlation tau is 0.56, the mean in these simulations is 0.64. This we suggest may be regarded as a conservative mean upper bound on what a perfect correlation would be as here we are correlating WT against itself, just allowing for sample size differences. The observed 0.56 is very close to the upper bound that we take as reassurance that the Foster mutational profile indeed very closely resembles that of the mass WT data.

Right at the end of their results section they compare the pattern in the Foster data to other large datasets from Sane et al. and Long et al.; why weren't these data used throughout?

**Response:** We aren’t sure where the referee got the idea that the Sane and Long data sets are “large” – our current set being much larger. As noted above to exclude selection it is best to test each experiment and include only good ones. These other sets are too small for meaningful testing. Per trinucleotide mutation rates per site are, for example, almost meaningless as so many trinucleotides have zero mutations, there being more trinucleotides than there are mutations per site.

The published Sane et al data, however, includes only some of their WT mutations. Following the referee’s suggestion and hoping to massively increase the sample size, we requested the journal to make the full Sane et al data set available (it isn’t public). We are grateful to both the journal and Sane et al for providing this. However, while what was originally published had a few tens of mutations this set only has 188 CDS WT mutations, so doesn’t greatly increase the sample size. Likewise, the WT data set from the Long et al had only a few tens of WT CDS mutations. A further data set that we now add (Tincher et al) is similarly small. We now however provide this larger WT only dataset and use it to a) compare with MMR and non-MMR KO strains and b) check original claims for resilience. Given that the Foster data (just the non MMR KOs) had both the same rate and same spectrum (claimed in the Foster et al 2015 paper) that motivated them to include them as wild type mutations, and given that we see strong concordance between them and WT mutations, we think our approach is both defendable and robust.

Inegative would be inclined to use these two datasets along with the non-KO lines of Foster and Wei to generate a larger dataset that does not include KO strains.

**Response:** We are slightly confused here as this is what we did and show that the conclusions are not affected – this is the point of the subsection “Results are not contingent on inclusion of non-MMR modified knockout lines”. We repeat this analysis using our new 1399 WT “pure” mutation set and find the same results. Or does the referee mean to make “pure” WT the focal set? However, if we look at this pure WT data alone (en mass) the Ka/Ks is just significant suggesting purifying selection may be an issue. By contrast, the Foster data has Ka/Ks very close to 1. We hope the referee concurs that it would be unwise then to employ this for the focal analysis.

Minor comments:

1. in the abstract replication is used in two contexts - replication of DNA and replication of an experimental result; this is confusing and should be resolved.

**Response:** Thank you – we hadn’t spotted this source of potential confusion – now hopefully resolved

2. they test whether there is selection on new mutations at non-synonymous sites by comparing the rates of mutation at the three codon positions in each of the tri-nucleotide contexts. This strikes me as being a weak test since some of the tri-nucleotide contexts will have relatively few entries. It would be interesting to see the mean difference and its standard error so we can judge whether this test has power.

**Response:** As the distributions aren’t normal, we shouldn’t really report mean and sem – but as it is requested, we have added them with necessary caveats.

3. "Simulant" should be "simulated" throughout.

**Response:** corrected

In summary, this is an interesting manuscript, but they need to do the analysis correctly. If the results remain unchanged then this would be a good manuscript for PB to publish.

**Response:** We hope that we have convinced you that at the very least our simulation approach is defendable and has numerous advantages over using raw count data. Moreover, we think the two approaches are rather complementary, both reporting that the “adaptive” parameters are near irrelevant.
