# Supplementary material for: The importance of genes does not predict their mutation rate
Source: PLoS Biol. 2026 Nov 12;24(11):e3004089. doi: 10.1371/journal.pbio.3004089 (PMC13759209; doi:10.1371/journal.pbio.3004089)
Supplement: Supplementary file 32 [file pbio.3004089.s059.docx]

**Response to referees:**

Reviewer #1:

I think the authors have decently responded to my comments. So i see no more trouble.

**Response:** Thank you


Reviewer #2:
[see review form attachment for fully formatted version]

I am satisfied that the authors have sufficiently addressed the issues raised. This includes substantial new content.

**Response:** Thank you

Nonetheless I am concerned that the way some of this new content is presented is less than fully clear.

Issues in text order:

Text 1 ‘expression’/’essentiality’ ‘predicts’ does not give the sign of the effect, which seems important (and is given elsewhere in the same column e.g. ‘essential genes have lower mutation density’)

**Response:** All notes in Text 1 now provide the direction of effects

"that permitted a 3-6 thousand generations"

confusing wording

**Response:** Changed to : “between 3 and 6 thousand generations”

“We find transcript abundance to again be negatively correlated with mutational density prior to partial correlation. It is weakly significant on partial correlation if gene length is removed as a parameter but not when it isn’t (Table S4).”

**Response:** Sorry this was misleading. It is now corrected to:

“It is weakly significant on partial correlation if gene length is permitted as a parameter (Table S4A) and when it isn’t (Table S4B).”

And we have modified the Table S4 file accordingly.

Table S4 has -0.07 **** for full correlation and -0.03* for partial correlation between mut_density and LB_transcript. I see nothing in the legend about removing gene length as a parameter and I don’t see a non-significant value when gene length is considered (or is ‘not when it isn’t’ meant to mean non-weakly, i.e. strongly significant; this is potentially ambiguous). Please clarify

**Response:** We have added the missing table to S4

"where only very few genes have any mutations, we approach what in regression would be a flat line"

This seems unintuitive – the relationship in question is mutational density against gene length – when one gene has a mutation and everything else has none, that seems maximally non-flat, even if there’s no strong expectation for what would happen in a regression (it might average out at flat, but won’t that depend on the distribution of gene lengths, so that might not be true?). Do clarify.

**Response:** We clarify: “(at the limit all genes have the same mutational density of zero)”

"While we cannot guarantee an absence of selection in each data set, the sample as a whole is tolerable, although Ka/Ks is low (Figure S18)."

If degree of selection is the axis of tolerability (?), what evidence is being used here, apart from Ka/Ks, to put this data above some threshold level? Please be more specific.

**Response:** We have reworded to clarify: it passes the by-site tests and the stop codon test and is borderline for Ka/Ks

"In no case out of 10,000 simulations [...] hence P <0.001"

True but confusing – for a 2 tailed test I might have interpreted that as P≤0.0005. Not giving a P value, although perhaps quoting a threshold from the sampling (e.g. ‘95% were less than an absolute correlation of x’) would give a clearer intuition for how big the observed correlation really is.

**Response:** We are here following the unbiased estimator of P, i.e. n/m , n being the number of observations greater than or equal to observed and m the number of simulations. We now cite:

Ewens WJ. On estimating P values by the Monte Carlo method. Am J Hum Genet. 2003 Feb;72(2):496-8. doi: 10.1086/346174.

who shows this to be the formula for the unbiased estimation of P. As there is an a priori expectation, one tailed is appropriate.

"the Foster knock out data"

Foster uses knockouts of MMR genes, but this phrase is being used (I think) explicitly to refer to the non-MMR gene knockouts from their study. This is clear in some places but not in others – where it’s unclear, it’s confusing – please clarify throughout.

**Response:** We now define these in the introduction as non-MMR KO and refer to them as such throughout.

"Results are unaffected by considering expression data from LB growth conditions alone, except that transcript abundance correlates significantly with mutation density upon full but not partial correlation analysis, as also seen when employing the fuller mutation data set (Table S18)."

A table should be referenced before the final phrase referring to TS18. I’d have thought Table S4 for LB data, except that there, the transcript-mutation density correlation is significant for both full and partial correlations.

**Response:** We agree this wasn’t clear enough. We now write:

“Results are unaffected by considering expression data from LB growth conditions alone (Table S18), except that transcript abundance correlates significantly with mutation density upon full but not partial correlation analysis.”

"the strand hypothesis"

Do be explicit what this is.

**Response:** We now write “to test the hypothesis that genes on the lagging strand have a higher mutation rate”.

"the transcript level effect, while remaining negative, is non-significant if we employ the expression data from LB media under Z scored partial correlation"

This seems to be contradicted by Table S4 which gives the -0.032 partial correlation 1-star significance.

**Response:** S4 doesn’t present the Z score results. These are in S7 (all mutations) and S18 (pure WT). We now write:

“First, the transcript level effect, while remaining negative, is non-significant if we employ the expression data from LB media under Z scored partial correlation (Table S7, Table S18). “

"the transcript level effect is seen if using data from LB conditions (although not on simulation)"

Is ‘simulation’ referring to the Z-test? Could do with being clearer and referencing the display item(s) this is based in.

**Response:** it is referring to the Z tests as above and we now clarify to point to the relevant tables

"Text S2"

This seems a helpful addition to the piece, but introducing both the methods and results that it represents in the Discussion seems out of place and much less helpful.

**Response:** we are hesitant to include it is the main results if only because the results seem so fragile. Where they sit in the discussion adds to the conclusions we wish to draw but not in any definitive manner.

"the resulting models have almost no accuracy"

I’m not sure it’s accuracy that’s lacking with the low R2, it’s explanatory power

**Response:** pseudo R^2 applied to binomial (or related) regression can’t be interpreted the same as R^2 for a linear repression model. Explanatory power seems then the wrong term as that could also be applied the r^2. The phrase often used is “[a measure of how well the model fits the data](https://medium.datadriveninvestor.com/understanding-nagelkerke-r²-pseudo-r²-d972ed9a00c4)”. For the pseudo-R^2 we have seen application of the term “accuracy” which accords with the above and which is our preference too.

"In E. coli, if protein level is ever significant"

Presumably this is referring to the one model (no. 4 in Text S2) where the LB_protein effect has P= 0.013 (?). Would be good to be explicit.
**Response:** we now point to S2.4

- I’m ok with the idea of their adding in the glms as an adjunct to the partial correlation analysis. I’ve suppressed strong urges to quibble with the authors’ defence of their approaches (e.g. in the rebuttal only, that ‘unlike any glm methods, these uniquely permit us to control for the bias in mutability of different trinucleotides’) because I don't believe that's what really what matters here. So I haven't gone back to say that, whether or not they use a Monte Carlo Z test to assess the significance of their correlations, it’s the effectiveness or otherwise of the underlying approach to disentangle multiple interacting effects in the presence of noise that concerns me and that’s where a glm approach is better. Partly, that's because their argument seems fair that others have claimed effects using nonparametric approaches and if effects were substantial, one might reasonably have expected them to be apparent even if partial correlations are underpowered.

I’m more worried now that overall it’s become a ±massive paper and therefore needs to be very clear, which isn’t uniformly the case. I haven't ranked my issues, but the most problematic ones seem to be presenting new analyses in the Discussion and that it seems likely the new text was not written using the submitted version of Table S4.

**Response:** we agree the paper can appear unwieldy, but we hope the referee agrees that if we wish to claim that certain very prominent claims don’t hold up, we must not leave ourselves open to claims of cherry picking data and we need to dissect prior methods. The Table S4 issue seems mostly a confusion between correlation results and Z score simulation based results. Pointing to the correct tables will remedy that.

Reviewer #3:

Comments on Radrizzani and Hurst "No evidence that genic importance predicts mutation rate variation"

Having read the replies to my comments and the revised manuscript I am satisfied with the revisions. I have no further comments.

Congratulations on a thorough and interesting piece of work.

**Response:** Thank you
