# Supplementary material for: The importance of genes does not predict their mutation rate
Source: PLoS Biol. 2026 Nov 12;24(11):e3004089. doi: 10.1371/journal.pbio.3004089 (PMC13759209; doi:10.1371/journal.pbio.3004089)
Supplement: Peer review history

# Editor report: Decision Letter 0

**DOI:** 10.1371/journal.pbio.3004089.r001

**Authors:** Roland Roberts

20 May 2026

Dear Laurence,

Thank you for submitting your revised manuscript entitled "No evidence that genic importance predicts mutation rate variation " for consideration as a Research Article by PLOS Biology.

As previously discussed, I'm writing to let you know that we would like to send your submission out for external peer review.

However, before we can send your manuscript to reviewers, we need you to complete your submission by providing the metadata that is required for full assessment. To this end, please login to Editorial Manager where you will find the paper in the 'Submissions Needing Revisions' folder on your homepage. Please click 'Revise Submission' from the Action Links and complete all additional questions in the submission questionnaire.

Once your full submission is complete, your paper will undergo a series of checks in preparation for peer review. After your manuscript has passed the checks it will be sent out for review. To provide the metadata for your submission, please Login to Editorial Manager (https://www.editorialmanager.com/pbiology) within two working days, i.e. by May 22 2026 11:59PM.

If your manuscript has been previously peer-reviewed at another journal, PLOS Biology is willing to work with those reviews in order to avoid re-starting the process. Submission of the previous reviews is entirely optional and our ability to use them effectively will depend on the willingness of the previous journal to confirm the content of the reports and share the reviewer identities. Please note that we reserve the right to invite additional reviewers if we consider that additional/independent reviewers are needed, although we aim to avoid this as far as possible. In our experience, working with previous reviews does save time.

If you would like us to consider previous reviewer reports, please edit your cover letter to let us know and include the name of the journal where the work was previously considered and the manuscript ID it was given. In addition, please upload a response to the reviews as a 'Prior Peer Review' file type, which should include the reports in full and a point-by-point reply detailing how you have or plan to address the reviewers' concerns.

During the process of completing your manuscript submission, you will be invited to opt-in to posting your pre-review manuscript as a bioRxiv preprint. Visit http://journals.plos.org/plosbiology/s/preprints for full details. If you consent to posting your current manuscript as a preprint, please upload a single Preprint PDF.

Feel free to email us at plosbiology@plos.org if you have any queries relating to your submission.

Cheers,

Roli

Roland Roberts, PhD

Senior Editor

PLOS Biology

rroberts@plos.org

# Peer review: Decision Letter 1

**DOI:** 10.1371/journal.pbio.3004089.r002

**Authors:** Roland Roberts

17 Jul 2026

Dear Laurence,

Thank you for your patience while your manuscript "No evidence that genic importance predicts mutation rate variation " was peer-reviewed at PLOS Biology. It has now been evaluated by the PLOS Biology editors, an Academic Editor with relevant expertise, and by three independent reviewers.

You'll see that reviewer #1 is very positive about the study and the importance of publishing negative results prominently. Most of his concerns can be addressed textually, but I can see one point where he suggests “a small simulation model”. Reviewer #2 is broadly positive; again, most of her/his requests look textual, but there are a few minor analyses needed, plus further coverage of the literature. Reviewer #3 is positive but questions whether rank correlation is an appropriate stats approach, instead suggesting a GLM with Poisson-distributed error (s/he suggests re-doing the analysis and adding a few additional ones). S/he also questions the choice of Foster and Wei for the dataset, suggesting instead to use a non-KO subset of this, along with data from Sane et al. and Long et al.

In light of the reviews, which you will find at the end of this email, we would like to invite you to revise the work to thoroughly address the reviewers' reports. IMPORTANT: I know that we have previously discussed the issues with the data availability from Sane et al., and given the request from rev #3, I've been in touch with those authors about full availability, and have been assured that they'll be able to share it with me (and thereby you) imminently; let me know if you're interested in this.

Given the extent of revision needed, we cannot make a decision about publication until we have seen the revised manuscript and your response to the reviewers' comments. Your revised manuscript is likely to be sent for further evaluation by all or a subset of the reviewers.

In addition to these revisions, you will need to complete some formatting changes, which you will receive in a follow up email. A member of our team will be in touch with a set of requests shortly.

We expect to receive your revised manuscript within 3 months. Please email us (plosbiology@plos.org) if you have any questions or concerns, or would like to request an extension.

At this stage, your manuscript remains formally under active consideration at our journal; please notify us by email if you do not intend to submit a revision so that we may withdraw it.

**IMPORTANT - SUBMITTING YOUR REVISION**

Your revisions should address the specific points made by each reviewer. Please submit the following files along with your revised manuscript:

1. A 'Response to Reviewers' file - this should detail your responses to the editorial requests, present a point-by-point response to all of the reviewers' comments, and indicate the changes made to the manuscript.

*NOTE: In your point-by-point response to the reviewers, please provide the full context of each review. Do not selectively quote paragraphs or sentences to reply to. The entire set of reviewer comments should be present in full and each specific point should be responded to individually, point by point.

You should also cite any additional relevant literature that has been published since the original submission and mention any additional citations in your response.

2. In addition to a clean copy of the manuscript, please also upload a 'track-changes' version of your manuscript that specifies the edits made. This should be uploaded as a "Revised Article with Changes Highlighted" file type.

*Re-submission Checklist*

When you are ready to resubmit your revised manuscript, please refer to this re-submission checklist: https://plos.io/Biology_Checklist

To submit a revised version of your manuscript, please go to https://www.editorialmanager.com/pbiology/ and log in as an Author. Click the link labelled 'Submissions Needing Revision' where you will find your submission record.

Please make sure to read the following important policies and guidelines while preparing your revision:

*Published Peer Review*

Please note while forming your response, if your article is accepted, you may have the opportunity to make the peer review history publicly available. The record will include editor decision letters (with reviews) and your responses to reviewer comments. If eligible, we will contact you to opt in or out. Please see here for more details:

https://blogs.plos.org/plos/2019/05/plos-journals-now-open-for-published-peer-review/

*PLOS Data Policy*

Please note that as a condition of publication PLOS' data policy (http://journals.plos.org/plosbiology/s/data-availability) requires that you make available all data used to draw the conclusions arrived at in your manuscript. If you have not already done so, you must include any data used in your manuscript either in appropriate repositories, within the body of the manuscript, or as supporting information (N.B. this includes any numerical values that were used to generate graphs, histograms etc.). For an example see here: http://www.plosbiology.org/article/info%3Adoi%2F10.1371%2Fjournal.pbio.1001908#s5

*Blot and Gel Data Policy*

We require the original, uncropped and minimally adjusted images supporting all blot and gel results reported in an article's figures or Supporting Information files. We will require these files before a manuscript can be accepted so please prepare them now, if you have not already uploaded them. Please carefully read our guidelines for how to prepare and upload this data: https://journals.plos.org/plosbiology/s/figures#loc-blot-and-gel-reporting-requirements

*Protocols deposition*

To enhance the reproducibility of your results, we recommend that if applicable you deposit your laboratory protocols in protocols.io, where a protocol can be assigned its own identifier (DOI) such that it can be cited independently in the future. Additionally, PLOS ONE offers an option for publishing peer-reviewed Lab Protocol articles, which describe protocols hosted on protocols.io. Read more information on sharing protocols at https://plos.org/protocols?utm_medium=editorial-email&utm_source=authorletters&utm_campaign=protocols

Thank you again for your submission to our journal. We hope that our editorial process has been constructive thus far, and we welcome your feedback at any time. Please don't hesitate to contact us if you have any questions or comments.

Sincerely,

Roli

Roland Roberts, PhD

Senior Editor

PLOS Biology

rroberts@plos.org

------------------------------------

REVIEWERS' COMMENTS:

Reviewer #1:

In their manuscript entitled "No evidence that genic importance predicts mutation rate variation ", Sofia Radrizzani1 and Laurence Hurst reanalyse mutation accumulation experiment to invalidate recent calls suggesting that local variation of mutation rate may be subject to selection.

This theme is important, because the idea that the control of mutation rate may be to some extant smarter than random noise has been always a tempting. On the one hand, most microbiologists that have no clue about evolution love the idea that mutation rate is controlled to create variation just when needed. For instance stress induced mutagenesis is necessarily a strategy to find mutations to resist to stress. Over the last decade, another type of scientists have tackled the question from the perspective of essential genes, this time having a lower mutation rate than the rest. While these types of selection in mutation rate is possible, it is extremely weak, and there are usually way stronger forces at play to explain the observed patterns (we debated that already in 2004 (Tenaillon et al Trends in microbiology 2004) . Here, the authors engage in this debate on the ground of the statistical analysis and type of data used to do these inference. After years of analysis of weird mutation reporters that created complex patterns, the gold strandard has become to study genomes of mutation accumulation. But due to the lack of mutations, scientists have used mutator lineage to make some of their derivations. Here the authors dig further un teh statistical baises inherent to the analysis of MA and contrast mutator and non mutator lineages. They show that in E. coli many hypothesised drivers of mutation rate are not relevant, exept expression at RNA level.

Overall the paper is very interesting, may be a bit too technical at some moments, which may make a class of the targeted readers (the microbiologist) less prone to get the message.

Here are some comments:

In the introduction, i think, it would be worth mentioning the intensity of selection applied to a control of mutation rate. TO mention that though a local control is not impossible, it remains tricky for natural selection to act on a local gene, unless the mechanisms affect a large panel of genes.

Page 4: We are unaware of any analysis attempting to control for all putative variables.

on the control of essentiality of genes and their impact of mutations found in these genes we found an effect while controlling for multiple factors in Vigué and Tenaillon (PNAS 2023) Where we showed that essentiality play a role in the purge of deleterious mutations more than expression over short time scales.

RNA seq median zero is not a great names for tables and discussions.

Relative to the RNAseq data, it could be worth knowing the overall types of environments sampled. Are there for instance a large number of stress conditions.

In the text the impact of RNA or protein abundances have different sign effect on gene length, this is not intuitive to me given they have the largest positive correlation.

I liked the control for gene length. Could a small simulation model tell when this type of artefact matters. I expect taht the signal should disappear when the number of mutation is large enough. That would be a valuable add on for people analysing this type of data in the future.

To me the main result of the E. coli part is that RNA level is associated to low mutation rate. As the author mention this is likely a combination of mfd coupled to MMR that results in excellent repair. The point is that, this type of observation need to be better debated to me to counteract the selectionist agenda. Indeed, we could here just use the argument that natural selection has favoured a repair mechanism that is more actif on highly expressed genes and indeed rather than just correcting for the mutagenic effect of transcription results in an even lower mutation rate. Moreover as the mechanism is generic and not gene specific, the selection for such mechanism is more possible. Furthermore as we published ( Feugeas et al MBE 2015) in a quite tough paper, (but the discussion is worth reading as we anchor on the same papers), under stress conditions MMR and mfd expression decline, which lead to what we think is this time a positive correlation between expression and mutagenesis, but only for genes expressed under stress. This is purely mechanistic, and while i think selection to lower mutation rate overall exists, the bacterial mutation rate is extremely low, the selection to increase it to find the best adaptive mutation is very unlikely at play here. But nevertheless if one look from a distance this interplay between expression of MMR, mfd and their super impact according to conditions could easily be fully interpreted in a selectionist agenda. This one would be more credible as only broad interactions would be at play and have genome wide effect upon which selection is more likely to play (for the lower rate, i am still not convinced that there is any recurrent engouh conditions where mutation is teh solution to stress to promote a signature of mutation rate that is favoring high mutation rate under stress). I think that the discussion should adress in a bit more detail these scenarios in saying how they match or not. The fact that the focus is on essentiality or protein abundances is to me moving away from this simple observations. For decades, molecular evolution could not distinguish between highly expressed and essential, (though as i mentioned we found a signal, having 80000 genomes and looking at very short time scale), so saying that essentiality is not popping up as a driver of gene importance may not be the strongest point. I think the last part of the discussion about the effect size may be better than saying a rejection of the hypothesis.

The part comparing the different patterns in MMR, is very informative, i think people underestimate the type of substractions we have to do when a system is lacking and what does its absence means for the mechanisms at play in replication and repair. Reminding this loud and clear is more than necessary.

The yeast data nicely confort the overall pattern. I am not as aware for yeast, but what is the mechanistic hytpotehes for the late expression gene high mutation rate and what type of genes are epxressed in these conditions.

Overall, i think the author have pointed out important limits to the mutation rate selectionist hypothesis. Papers supporting these hypothesis are always welcome in fancy journals because adaptationist hypotheses are always more attractive than noise. Paper that debunk these data are less sexy by definition: more technical and negative; but it is key to publish them in high impact journal such as PLoS Biology to give support to thorough science.

Olivier Tenaillon (I always sign my reviews)

Reviewer #2:

This is an impressively thorough dissection of potential correlates of mutation in the two key model microbes where the range of published data is sufficient to do so. It needs to be thorough, because the authors attempt to demonstrate a negative - a lack of a role for gene importance in among-gene variation in mutation. This is, a priori, a hard thing to do and at one level impossible. This means that in many ways the most robust part of this study (that should perhaps get more prominence) is their final discussion point - that even if there is an effect of gene importance on mutation rate, that effect must be very small relative to other effects. Among secondary findings, the biasing effects of using mutator strains seems particularly worthwhile (and perhaps deserves further discussion in the context of inter-organism evolution of DNA repair). Nonetheless, the dissection of correlations is the core and clearly focused against adaptationist hypotheses of mutation rate variation. Such hypotheses are remarkably pervasive and tenacious, making this assessment of evidence used to support such hypotheses important, worthwhile and very appropriate to the journal.

The approach taken is a somewhat idiosyncratic mixture of sequence randomisation approaches that are both powerful and elegant, with (partial) correlation approaches that are neither of those two things. The large number of distribution plots and correlation tables arising are not the most user friendly (particularly in interaction with the journal's bitty approach to supplementary material), but are at least a consistent interface for the reader.

Issues arising which roughly fall into more and less important

More important:

Better justification of the (partial) correlation approach would be helpful - it feels like a linear modelling approach, perhaps with a binomial response of mutation/not, accounting for interactions among multiple explanatory variables (or even a machine learning approach), would have been both easier to grasp and more powerful. It would bring in assumptions about (lack of) error in explanatory variables, which would undoubtedly be an issue for the expression data (only), but error there (particularly the potential for different amounts for RNA and protein data) is an issue for the logic as it is (and something the authors address).

As is made clear in the first sentence of the discussion, there are two adaptive conjectures leading to opposite expectations for 'important' genes. To be a fully convincing refutation of such selectionist ideas it does feel that the authors would need to take more seriously the possibility that both things are going on at the same time, leading to their observed lack of clear correlations of mutation density with 'important' genes. That could mean more complex/comprehensive models or greater clarity over/dissection of 'important', but at least some discussion would be helpful.

The finding that a rank-based statistic can get messed up by lots of repeated values (in this case zero mutation counts) doesn't feel very novel, but perhaps deserves the given highlighting in this context. It would also seem to add weight to the currently un-discussed possibility of using a GLM where lots of zeros in a Poisson or Binomial model are very much normal.

The response variable here is 'mutation density', a phrase which is used pretty much everywhere except the early parts of the Methods, where we need to know exactly what it is and how extracted from the data described. More generally it would be helpful to connect it precisely to (different) definitions of mutation rate, which is what is invoked in the title. Probably related to this, different numbers of generations presumably occurred in the different MA studies (and perhaps within them?), so saying explicitly how this was dealt with and/or what effect that has on the possibility of interpreting the findings seems important.

Statistical power and (non-) significance are important here, but the first section of the Results asking about evidence for selection in the MA data used, seemed the least effective at dealing with them. Specifically there is a clear hypothesis that, if there's selection, mutation by position would be "3 > 1 > 2", yet we are never told what the best estimate of the observed order is (we should be, even if there are only 6 options and no statistical significance!). We only have what feels like a very under-powered test (multiple instances of a paired Wilcoxon test) coming out as non-significant.

This questionable approach is coupled with a lack of engagement with others' work that does find evidence of selection in mutation accumulation - I'm thinking of these papers, though there may be others:

Mahilkar, A., Raj, N., Kemkar, S. & Saini, S. (2022) Selection in a growing colony biases results of mutation accumulation experiments. Scientific reports 12, 15470. 10.1038/s41598-022-19928-5

Wahl, L. M. & Agashe, D. (2022) Selection bias in mutation accumulation. Evolution 10.1111/evo.14430

These issues should be addressed more directly.

Less important

What is and isn't a 'good' correlation could usefully be more consistent - there is one paragraph where two different correlations, each of 0.3 are referred to very differently. This does correspond to the visual impression of the correlations (S17A seems a clear overall relationship, 5B does not), but just being more explicit about that might help.

The orthogonal regression lines in the figures don't seem to add much, beyond hinting at the idea that these data aren't all that linear, and that allowing variation on both axes doesn't get round that issue or the issue that outliers can have undue influence on regression lines. A 1:1 line (where it intersects the region) would seem more helpful.

Ref 99 is used for duplex sequencing, but isn't about that - should it perhaps be this?: Bhawsinghka, N., Burkholder, A. & Schaaper, R. M. (2023) Detection of DNA replication errors and 8-oxo-dGTP-mediated mutations in E. coli by Duplex DNA Sequencing. DNA repair 123, 103462. 10.1016/j.dnarep.2023.103462

The findings of very different characteristics of the data from the two labs is intriguing - could the authors speculate about any methodological differences between the studies (different media leading to different Ne??)

It seems a little wrong to introduce novel findings about two new species in the discussion. Could this be added to the end of the results?

Probably published since this was submitted, but this paper seems to be another reanalysis of much of the same data, although addressing different questions, so some assessment/discussion might be appropriate: Jago, M. J. et al. (2026) Extended sequence context shapes mutational bias in Escherichia coli. Proceedings of the National Academy of Sciences 123 https://doi.org/rbht

It's somewhat confusing to have Ka/Ks methods given their own section, but then also appearing with a much more cursory description referring to method details elsewhere (ref 64) - if these are different sets of data, the relationship should be clearer.

Particularly in the abstract but potentially elsewhere too, using 'replication' and 'replicatory' in adjacent sentences to apply to completely different things is confusing. Do rephrase.

Not sure if it's a typo or my mis-reading, but 'explain less than 0.5% of the variation…R2 = 0.049' seems wrong.

Reviewer #3:

Comments on Radrizzani and Hurst "No evidence that genic importance predicts mutation rate variation"

In this interesting manuscript the authors reconsider the question of whether the mutation rate in genes is affected by the importance of the gene using data from several mutation accumulation experiments. They focus primarily on E.coli since this is one of the species for which claims of adaptive mutation rate variation have been made, and for which there is a lot of data. They show that there is little evidence of natural selection acting upon the accumulated mutations. They then demonstrate that there no evidence that more important genes have lower (or higher ) mutation rates when various confounding variables are taken into account.

While I think these are interesting results, their statistical approach is not appropriate and they find that the method is biased with respect to gene length. This does not surprise me - a rank correlation is going to struggle with this type of data. The solution is to use a general linear model with a Poisson distributed error. This is a standard method for dealing with count data, and if they find that their error is overdispersed then they can use negative binomial regression. This approach has lots of benefits - it is a well-established method with well understood statistical properties, fixed effects (such as leading/lagging strand) are easily accommodated and critically they can get standard errors on the estimates so they can establish whether they actually have the power to determine whether there are effects. The effect of gene length will be treated properly (I think gene length needs to included in the model as the log of gene length because in Poisson regression the log of the mean of the mean of the Poisson is modelled as a linear combination of the independent factors in the model). So, the analysis should be redone using this approach; this will simplify the manuscript eliminating lots of unnecessary simulation and analysis. One could even compare the results for the MMR-deficient and wild-type strains by including a fixed effect and interaction terms. redoing the manuscript using this technique will not take long.

It is unclear why they focussed on the data from Foster and Wei, particularly given that the Foster data comprises data from strains with other repair genes that have been knocked out. They show the mutational profiles of these KO strains are correlated to that of the wild-type strains but the correlations are not particularly strong and only slightly stronger than the correlation to the MMR strains which they have shown give different results in their main analysis to the WT strains. Are they as strong as we might expect? They could address this with simulations. Right at the end of their results section they compare the pattern in the Foster data to other large datasets from Sane et al. and Long et al.; why weren't these data used throughout? I would be inclined to use these two datasets along with the non-KO lines of Foster and Wei to generate a larger dataset that does not include KO strains.

Minor comments:

1.in the abstract replication is used in two contexts - replication of DNA and replication of an experimental result; this is confusing and should be resolved.

2.they test whether there is selection on new mutations at non-synonymous sites by comparing the rates of mutation at the three codon positions in each of the tri-nucleotide contexts. This strikes me as being a weak test since some of the tri-nucleotide contexts will have relatively few entries. It would be interesting to see the mean difference and its standard error so we can judge whether this test has power.

3."Simulant" should be "simulated" throughout.

In summary, this is an interesting manuscript, but they need to do the analysis correctly. If the results remain unchanged then this would be a good manuscript for PB to publish.

# Author comment: Author response to Decision Letter 2

**DOI:** 10.1371/journal.pbio.3004089.r003

8 Sep 2026

Attachment Submitted filename: responses05092026.docx

# Peer review: Decision Letter 2

**DOI:** 10.1371/journal.pbio.3004089.r004

**Authors:** Roland Roberts

8 Oct 2026

Dear Laurence,

Thank you for your patience while we considered your revised manuscript "No evidence that genic importance predicts mutation rate variation " for publication as a Research Article at PLOS Biology. This revised version of your manuscript has been evaluated by the PLOS Biology editors, the Academic Editor and the original reviewers.

Based on the reviews, we are likely to accept this manuscript for publication, provided you satisfactorily address the remaining points raised by reviewer #2 and the following data and other policy-related requests.

IMPORTANT - please attend to the following:

a) We like Titles to contain an active verb; could you possibly change yours to something more declarative like: "The importance of genes does not predict their mutation rate"?

b) Please change your Competing Interests statement to read: "I have read the journal’s policy and the authors of this manuscript have the following competing interests: LDH serves on the Editorial Board of PLOS Biology. All the other authors have declared that no competing interests exist."

c) Please address my Data Policy requests below; specifically, we need you to supply the numerical values underlying Figs 1BCDEFGHI, 2ABCDEFGH, 3ABC, 4ABCD, 5ABC, 6ABCDEFGH, and all supp Figs, either as a supplementary data file or as a permanent DOI’d deposition. I note that you already have an associated Zenodo deposition (https://doi.org/10.5281/zenodo.22256449), which seems to be very well organised and comprehensive. Please could you confirm whether the data and code in this deposition are sufficient to recreate the Figures?

d) Please cite the location of the data/code clearly in all relevant main and supplementary Figure legends, e.g. “The data and code needed to reproduce this Figure can be found in https://doi.org/10.5281/zenodo.22256449"; also update your Data Availability Statement accordingly.

As you address these items, please take this last chance to review your reference list to ensure that it is complete and correct. If you have cited papers that have been retracted, please include the rationale for doing so in the manuscript text, or remove these references and replace them with relevant current references. Any changes to the reference list should be mentioned in the cover letter that accompanies your revised manuscript.

In addition to these revisions, you may need to complete some formatting changes, which you will receive in a follow up email. A member of our team will be in touch with a set of requests shortly. If you do not receive a separate email within a few days, please assume that checks have been completed, and no additional changes are required.

We expect to receive your revised manuscript within two weeks.

To submit your revision, please go to https://www.editorialmanager.com/pbiology/ and log in as an Author. Click the link labelled 'Submissions Needing Revision' to find your submission record. Your revised submission must include the following:

- a cover letter that should detail your responses to any editorial requests, if applicable, and whether changes have been made to the reference list

- a Response to Reviewers file that provides a detailed response to the reviewers' comments (if applicable, if not applicable please do not delete your existing 'Response to Reviewers' file.)

- a track-changes file indicating any changes that you have made to the manuscript.

NOTE: If Supporting Information files are included with your article, note that these are not copyedited and will be published as they are submitted. Please ensure that these files are legible and of high quality (at least 300 dpi) in an easily accessible file format. For this reason, please be aware that any references listed in an SI file will not be indexed. For more information, see our Supporting Information guidelines:

https://journals.plos.org/plosbiology/s/supporting-information

*Published Peer Review History*

Please note that you may have the opportunity to make the peer review history publicly available. The record will include editor decision letters (with reviews) and your responses to reviewer comments. If eligible, we will contact you to opt in or out. Please see here for more details:

https://plos.org/published-peer-review-history/

*Press*

Should you, your institution's press office or the journal office choose to press release your paper, please ensure you have opted out of Early Article Posting on the submission form. We ask that you notify us as soon as possible if you or your institution is planning to press release the article.

*Protocols deposition*

To enhance the reproducibility of your results, we recommend that if applicable you deposit your laboratory protocols in protocols.io, where a protocol can be assigned its own identifier (DOI) such that it can be cited independently in the future. Additionally, PLOS ONE offers an option for publishing peer-reviewed Lab Protocol articles, which describe protocols hosted on protocols.io. Read more information on sharing protocols at https://plos.org/protocols?utm_medium=editorial-email&utm_source=authorletters&utm_campaign=protocols

Please do not hesitate to contact me should you have any questions.

Sincerely,

Roli

Roland Roberts, PhD

Senior Editor

rroberts@plos.org

PLOS Biology

------------------------------------------------------------------------

DATA POLICY:

You may be aware of the PLOS Data Policy, which requires that all data be made available without restriction: http://journals.plos.org/plosbiology/s/data-availability. For more information, please also see this editorial: http://dx.doi.org/10.1371/journal.pbio.1001797

Note that we do not require all raw data. Rather, we ask that all individual quantitative observations that underlie the data summarized in the figures and results of your paper be made available in one of the following forms:

1) Supplementary files (e.g., excel). Please ensure that all data files are uploaded as 'Supporting Information' and are invariably referred to (in the manuscript, figure legends, and the Description field when uploading your files) using the following format verbatim: S1 Data, S2 Data, etc. Multiple panels of a single or even several figures can be included as multiple sheets in one excel file that is saved using exactly the following convention: S1_Data.xlsx (using an underscore).

2) Deposition in a publicly available repository. Please also provide the accession code or a reviewer link so that we may view your data before publication.

Regardless of the method selected, please ensure that you provide the individual numerical values that underlie the summary data displayed in the following figure panels as they are essential for readers to assess your analysis and to reproduce it: Figs 1BCDEFGHI, 2ABCDEFGH, 3ABC, 4ABCD, 5ABC, 6ABCDEFGH, and all supp Figs. NOTE: the numerical data provided should include all replicates AND the way in which the plotted mean and errors were derived (it should not present only the mean/average values).

IMPORTANT: Please also ensure that figure legends in your manuscript include information on where the underlying data can be found, and ensure your supplemental data file/s has a legend.

Please ensure that your Data Statement in the submission system accurately describes where your data can be found.

------------------------------------------------------------------------

CODE POLICY

Per journal policy, if you have generated any custom code during the course of this investigation, please make it available without restrictions. Please ensure that the code is sufficiently well documented and reusable, and that your Data Statement in the Editorial Manager submission system accurately describes where your code can be found. More information on our Code Policy, what and how to share can be found here: https://journals.plos.org/plosbiology/s/code-availability

Please note that we cannot accept sole deposition of code in GitHub, as this could be changed after publication. However, you can archive this version of your publicly available GitHub code to Zenodo. Once you do this, it will generate a DOI number, which you will need to provide in the Data Accessibility Statement (you are welcome to also provide the GitHub access information). See the process for doing this here: https://docs.github.com/en/repositories/archiving-a-github-repository/referencing-and-citing-content

------------------------------------------------------------------------

DATA NOT SHOWN?

- Please note that per journal policy, we do not allow the mention of "data not shown", "personal communication", "manuscript in preparation" or other references to data that is not publicly available or contained within this manuscript. Please either remove mention of these data or provide figures presenting the results and the data underlying the figure(s).

------------------------------------------------------------------------

REVIEWERS' COMMENTS:

Reviewer #1:

I think the authors have decently responded to my comments. So i see no more trouble.

Reviewer #2:

[see review form attachment for fully formatted version]

I am satisfied that the authors have sufficiently addressed the issues raised. This includes substantial new content. Nonetheless I am concerned that the way some of this new content is presented is less than fully clear.

Issues in text order:

Text 1 ‘expression’/’essentiality’ ‘predicts’ does not give the sign of the effect, which seems important (and is given elsewhere in the same column e.g. ‘essential genes have lower mutation density’)

"that permitted a 3-6 thousand generations"

confusing wording

“We find transcript abundance to again be negatively correlated with mutational density prior to partial correlation. It is weakly significant on partial correlation if gene length is removed as a parameter but not when it isn’t (Table S4).”

Table S4 has -0.07 **** for full correlation and -0.03* for partial correlation between mut_density and LB_transcript. I see nothing in the legend about removing gene length as a parameter and I don’t see a non-significant value when gene length is considered (or is ‘not when it isn’t’ meant to mean non-weakly, i.e. strongly significant; this is potentially ambiguous). Please clarify

"where only very few genes have any mutations, we approach what in regression would be a flat line"

This seems unintuitive – the relationship in question is mutational density against gene length – when one gene has a mutation and everything else has none, that seems maximally non-flat, even if there’s no strong expectation for what would happen in a regression (it might average out at flat, but won’t that depend on the distribution of gene lengths, so that might not be true?). Do clarify.

"While we cannot guarantee an absence of selection in each data set, the sample as a whole is tolerable, although Ka/Ks is low (Figure S18)."

If degree of selection is the axis of tolerability (?), what evidence is being used here, apart from Ka/Ks, to put this data above some threshold level? Please be more specific.

"In no case out of 10,000 simulations [...] hence P <0.001"

True but confusing – for a 2 tailed test I might have interpreted that as P≤0.0005. Not giving a P value, although perhaps quoting a threshold from the sampling (e.g. ‘95% were less than an absolute correlation of x’) would give a clearer intuition for how big the observed correlation really is.

"the Foster knock out data"

Foster uses knockouts of MMR genes, but this phrase is being used (I think) explicitly to refer to the non-MMR gene knockouts from their study. This is clear in some places but not in others – where it’s unclear, it’s confusing – please clarify throughout.

"Results are unaffected by considering expression data from LB growth conditions alone, except that transcript abundance correlates significantly with mutation density upon full but not partial correlation analysis, as also seen when employing the fuller mutation data set (Table S18)."

A table should be referenced before the final phrase referring to TS18. I’d have thought Table S4 for LB data, except that there, the transcript-mutation density correlation is significant for both full and partial correlations.

"the strand hypothesis"

Do be explicit what this is.

"the transcript level effect, while remaining negative, is non-significant if we employ the expression data from LB media under Z scored partial correlation"

This seems to be contradicted by Table S4 which gives the -0.032 partial correlation 1-star significance.

"the transcript level effect is seen if using data from LB conditions (although not on simulation)"

Is ‘simulation’ referring to the Z-test? Could do with being clearer and referencing the display item(s) this is based in.

"Text S2"

This seems a helpful addition to the piece, but introducing both the methods and results that it represents in the Discussion seems out of place and much less helpful.

"the resulting models have almost no accuracy"

I’m not sure it’s accuracy that’s lacking with the low R2, it’s explanatory power

"In E. coli, if protein level is ever significant"

Presumably this is referring to the one model (no. 4 in Text S2) where the LB_protein effect has P= 0.013 (?). Would be good to be explicit.

- I’m ok with the idea of their adding in the glms as an adjunct to the partial correlation analysis. I’ve suppressed strong urges to quibble with the authors’ defence of their approaches (e.g. in the rebuttal only, that ‘unlike any glm methods, these uniquely permit us to control for the bias in mutability of different trinucleotides’) because I don't believe that's what really what matters here. So I haven't gone back to say that, whether or not they use a Monte Carlo Z test to assess the significance of their correlations, it’s the effectiveness or otherwise of the underlying approach to disentangle multiple interacting effects in the presence of noise that concerns me and that’s where a glm approach is better. Partly, that's because their argument seems fair that others have claimed effects using nonparametric approaches and if effects were substantial, one might reasonably have expected them to be apparent even if partial correlations are underpowered.

I’m more worried now that overall it’s become a ±massive paper and therefore needs to be very clear, which isn’t uniformly the case. I haven't ranked my issues, but the most problematic ones seem to be presenting new analyses in the Discussion and that it seems likely the new text was not written using the submitted version of Table S4.

Reviewer #3:

Comments on Radrizzani and Hurst "No evidence that genic importance predicts mutation rate variation"

Having read the replies to my comments and the revised manuscript I am satisfied with the revisions. I have no further comments.

Congratulations on a thorough and interesting piece of work.

Attachment Submitted filename: review2.pdf

# Author comment: Author response to Decision Letter 3

**DOI:** 10.1371/journal.pbio.3004089.r005

13 Oct 2026

Attachment Submitted filename: Response to referees2.docx

# Editor report: Decision Letter 3

**DOI:** 10.1371/journal.pbio.3004089.r006

**Authors:** Roland Roberts

16 Oct 2026

Dear Laurence,

Thank you for the submission of your revised Research Article "The importance of genes does not predict their mutation rate" for publication in PLOS Biology. On behalf of my colleagues and the Academic Editor, Deepa Agashe, I'am pleased to say that we can in principle accept your manuscript for publication, provided you address any remaining formatting and reporting issues. These will be detailed in an email you should receive within 2-3 business days from our colleagues in the journal operations team; no action is required from you until then. Please note that we will not be able to formally accept your manuscript and schedule it for publication until you have completed any requested changes.

Please take a minute to log into Editorial Manager at http://www.editorialmanager.com/pbiology/, click the "Update My Information" link at the top of the page, and update your user information to ensure an efficient production process.

PRESS: We frequently collaborate with press offices. If your institution or institutions have a press office, please notify them about your upcoming paper at this point, to enable them to help maximise its impact. If the press office is planning to promote your findings, we would be grateful if they could coordinate with biologypress@plos.org. If you have previously opted in to the early version process, we ask that you notify us immediately of any press plans so that we may opt out on your behalf.

We also ask that you take this opportunity to read our Embargo Policy regarding the discussion, promotion and media coverage of work that is yet to be published by PLOS. As your manuscript is not yet published, it is bound by the conditions of our Embargo Policy. Please be aware that this policy is in place both to ensure that any press coverage of your article is fully substantiated and to provide a direct link between such coverage and the published work. For full details of our Embargo Policy, please visit http://www.plos.org/about/media-inquiries/embargo-policy/.

Thank you again for choosing PLOS Biology for publication and supporting Open Access publishing. We look forward to publishing your study.

Sincerely,

Roli

Roland G Roberts, PhD, PhD

Senior Editor

PLOS Biology

rroberts@plos.org
